# Supplementary material for: IGFBP3 repression driven by inflammation links air pollution to placental and developmental defects
Source: EMBO Mol Med. 2026 Mar 24;18(5):1648–78. doi: 10.1038/s44321-026-00403-x (PMC13179339; doi:10.1038/s44321-026-00403-x)
Supplement: Supplementary file 1 — Appendix [file 44321_2026_403_MOESM1_ESM.pdf]

IGFBP3 Repression driven by inflammation links Air Pollution to Placental and Developmental defects

This PDF file includes:

Appendix Figures S1 to S16……………………………………………………………………………………………………2-15

Appendix Table S1 ……………………………………………………………………………………………………16-17

Appendix Figures

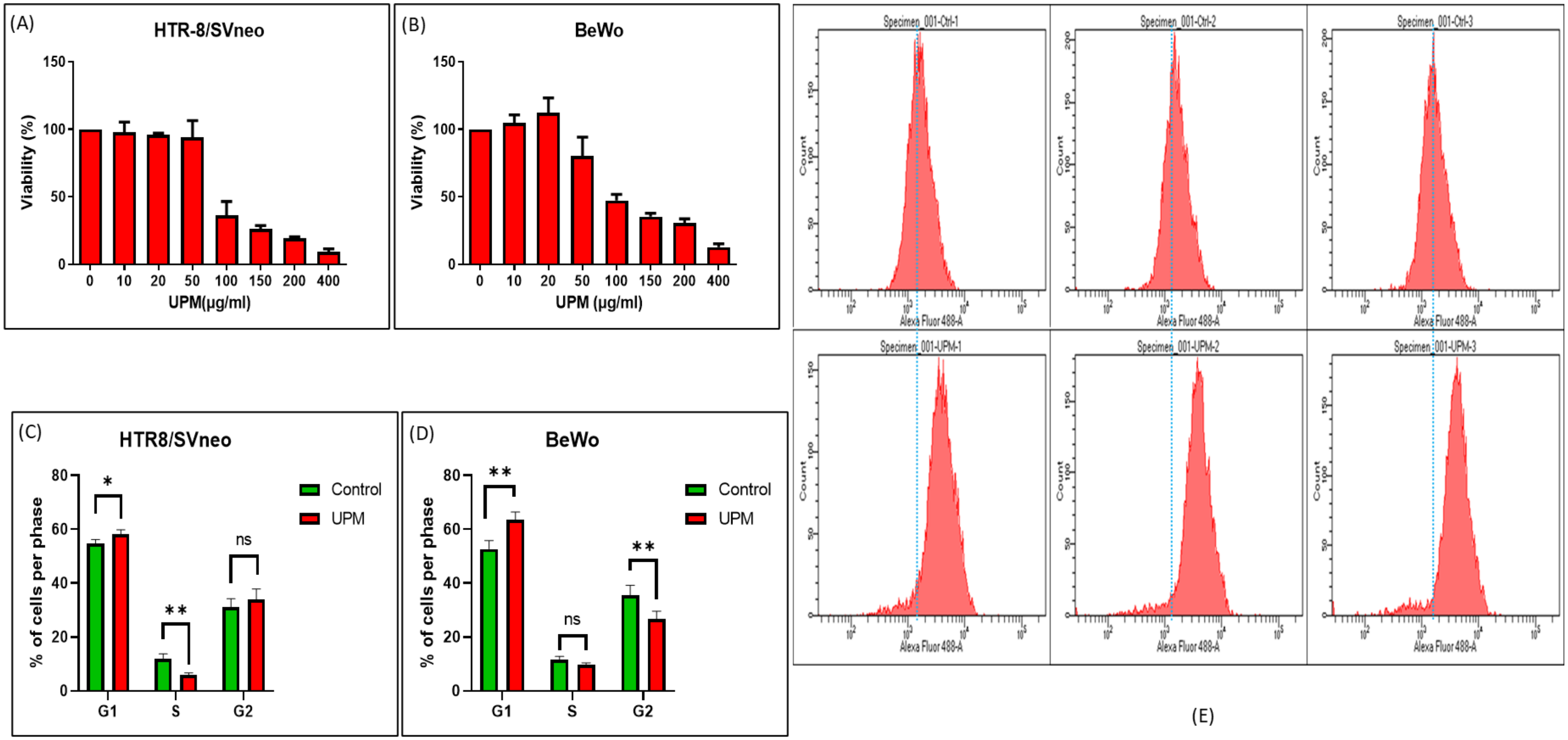

Appendix Figure S1. Effect of UPM on the trophoblast cell cycle progression(A-B), cells viability(C-D), and (E) HTR8/SVneo cells were stained with 5µM-CellROX followed by analysis using flow cytometer.

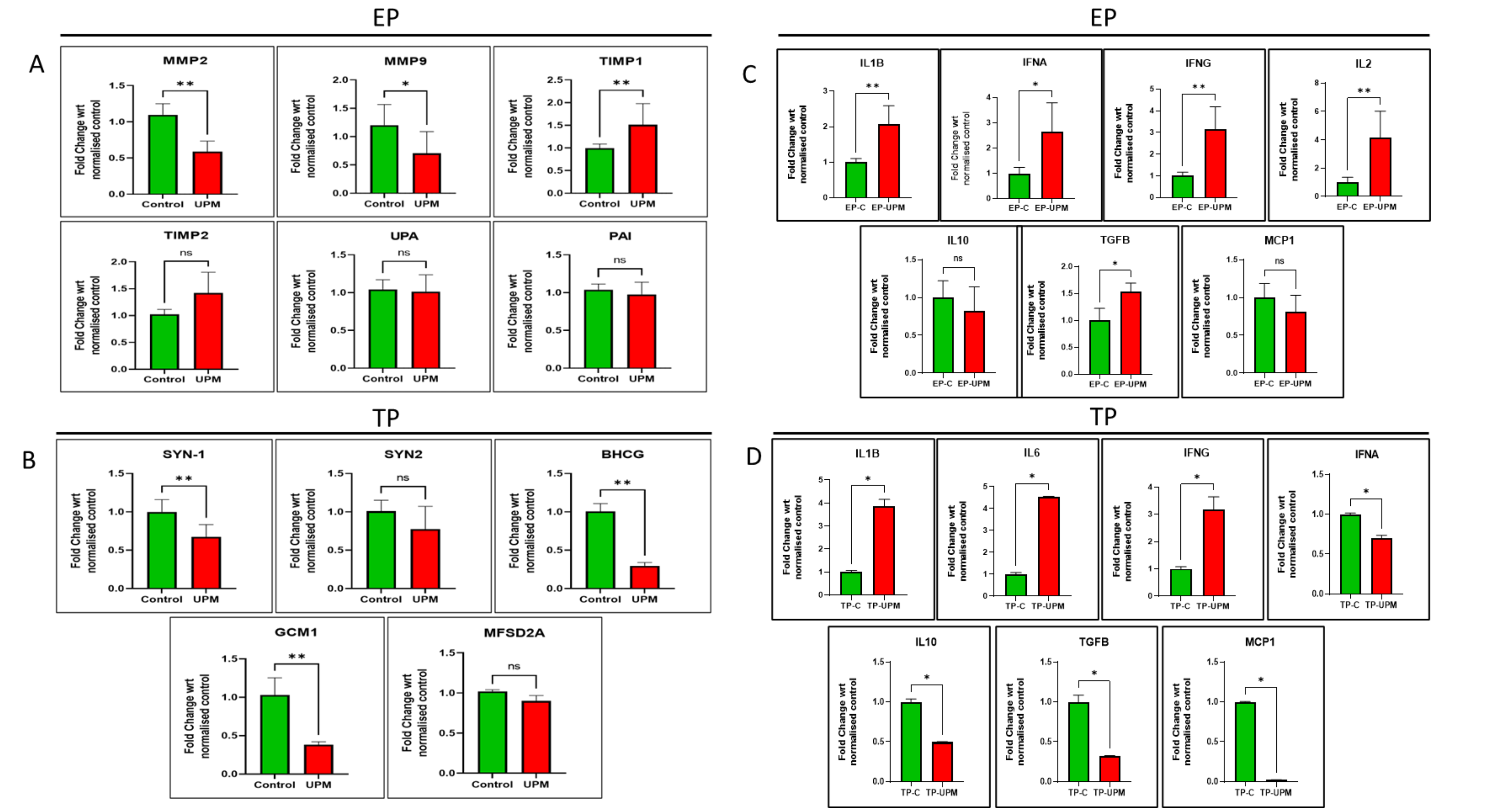

Appendix Figure S2. mRNA expression of invasion related (A), syncytialization (B), inflammatory and anti-inflammatory (C&D) genes in UPM exposed EP and TP explants. Statistical analysis was done by comparing the fold change in UPM stimulated group with respect to the unexposed group. The results were analyzed by the 2−△△CT method and data are graphically represented. All data are presented as mean ± SD. n≥5, *p<0.05; **p<0.01; ***p<0.001 ns: non-significant.

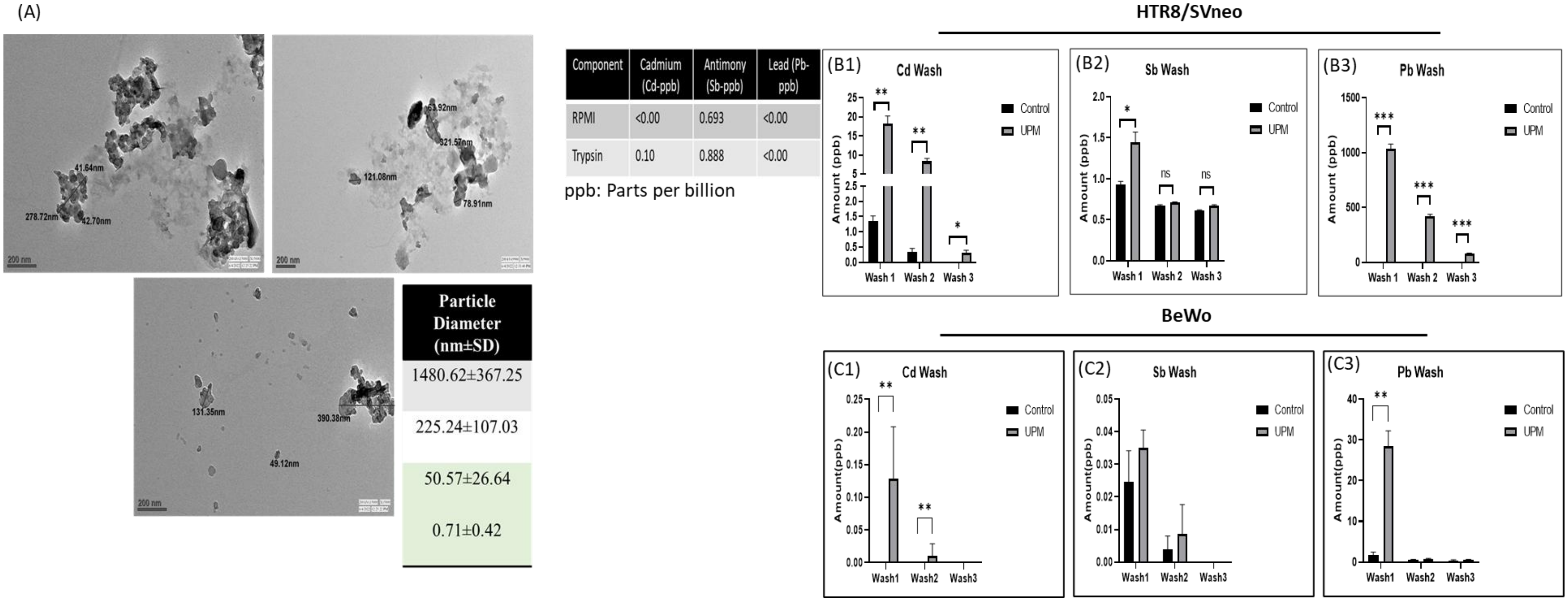

Appendix Figure S3A. ICP-MS for element analysis in UPM stimulated HTR8/SVneo and BeWo cells:
(A) TEM image(negative staining) for the UPM
(B1-C3) ICP-MS data for metals quantification in the RPMI(used for washing cells) for 3 subsequent washes. (B1-B3) Data for HTR8/SVneo and (C1-C3) Data for BeWo cells.

UPM mediated dysregulation of transcriptional landscape in trophoblast cells

❖ **Samples: UPM exposed HTR8/SVneo cells and EP explants**

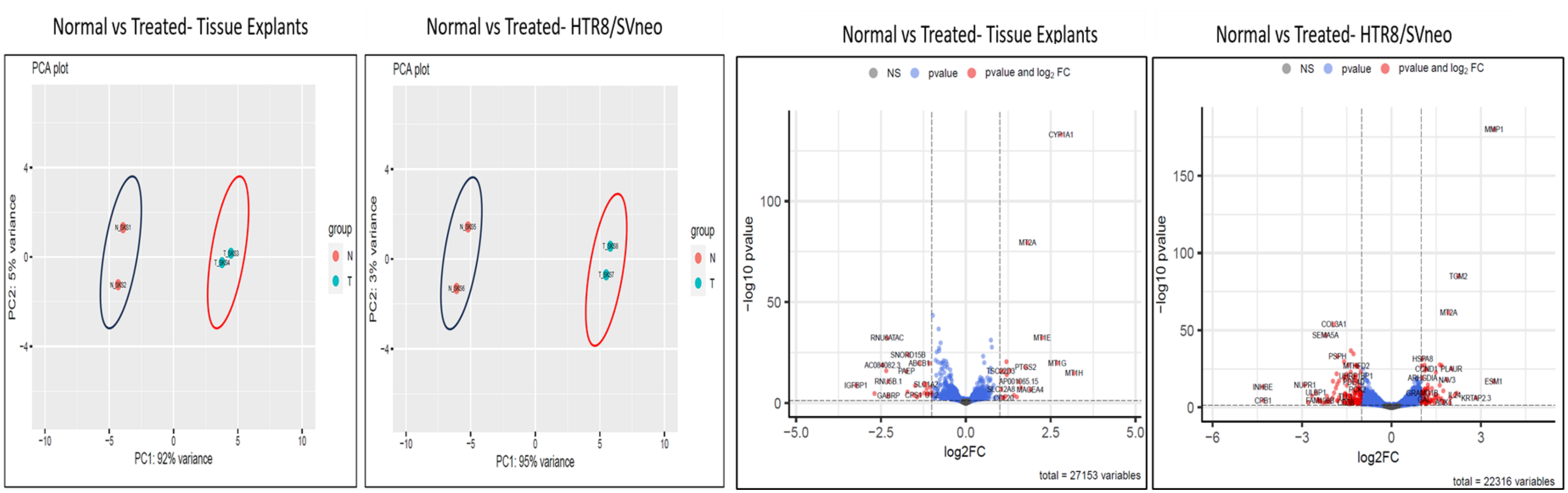

Appendix Figure S4. Left panel: PCA plot for UPM treated early placental villi explants and HTR8/SVneo cells. PCA clustering of control (red) and UPM (blue) samples. N: Control; T: UPM treated
Right Panel: Volcano plot showing the differentially expressed genes in UPM treated early placental villi explants and HTR8/SVneo cells. Horizontal line shows pvalue 0.5 threshold and vertical lines represent log2fold change cut-off of -1 and +1. (N: Control; T: UPM treated explants).

Gene	log2FoldChange	Padj
INHBE	-4.32	9.75E-12
SMIM14	-1.50	9.69E-13
CHAC1	-1.41	9.50E-16
COL5A1	-1.18	9.50E-16
EMP1	1.03	9.11E-09
CH507.513H4.5	1.62	8.89E-13
TXNRD1	1.01	8.71E-25
HSPA1B	1.69	8.59E-25
NETO1	-1.84	8.57E-20
CCND1	1.17	8.03E-23
GPX1	1.35	7.74E-11
GPT2	-1.16	7.55E-13
PCDH10	-1.55	7.43E-08
ATOH8	-1.06	7.43E-07
NUPR1	-2.90	7.41E-13
U1.6	1.24	7.21E-07
IL24	2.12	7.21E-07

Gene	log2FoldChange	Padj
IGFBP3	-1.59	7.14E-18
ARHGAP28	-1.24	7.02E-07
SLC1A4	-1.37	7.00E-21
HMGA1	1.12	6.64E-22
PLAUR	2.01	6.46E-23
PDE4D	-1.26	6.25E-15
TGM2	2.24	6.13E-82
SEPW1	1.22	5.52E-13
SMTN	1.22	5.32E-11
NAV3	1.86	5.25E-16
TMEM158	2.19	5.15E-08
DDIT3	-1.02	5.13E-07
TRIB3	-1.03	5.10E-09
HSPA8	1.05	5.09E-29
ALDH1L2	-1.86	4.84E-11
GRAMD1B	1.03	4.76E-08
LMO4	-1.20	4.56E-10
CD24	-1.02	4.42E-07

Gene	log2FoldChange	Padj
DDR2	-1.36	4.24E-34
HSP90AA1	1.11	4.22E-25
OLFML2A	-1.04	4.22E-08
PSAT1	-1.45	4.03E-25
FAM84B	-1.20	4.03E-25
COL3A1	-1.93	4.01E-51
SRM	1.03	3.85E-08
ESM1	3.42	3.66E-15
SLC7A11	-1.28	3.60E-32
LAMB3	1.09	3.49E-08
SEMA5A	-2.21	3.46E-44
SVEP1	-1.19	3.41E-08
JDP2	-1.49	3.32E-18
CBX5	-1.03	3.31E-21
SLFN5	-1.28	3.23E-09
HTRA1	-1.52	3.23E-07
STC2	-1.59	3.17E-27
PTPRQ	-1.93	3.16E-08

Gene	log2FoldChange	Padj
RRP9	1.01	3.16E-08
MYPN	1.28	3.16E-08
OLR1	-1.22	3.01E-08
TSC22D3	-1.96	2.99E-15
FOSL1	1.62	2.83E-25
GRPR	1.47	2.81E-07
FAH	1.10	2.78E-09
GARS	-1.11	2.76E-24
CEBPG	-1.20	2.70E-18
LAMA4	1.20	2.62E-10
PCK2	-1.15	2.61E-10
ARHGDIA	1.05	2.49E-17
SMAD9	-1.05	2.41E-07
SEMA3D	-1.02	2.36E-08
NCOA7	-1.15	2.31E-15
ULBP1	-2.54	2.26E-08
EDIL3	-1.10	2.26E-07
BAG3	1.09	2.23E-08

Gene	log2FoldChange	Padj
GPNMB	-1.57	2.05E-16
ADM2	-1.76	1.97E-15
HSPE1	1.09	1.97E-07
ALPK2	-1.82	1.96E-13
DDIT4	-1.57	1.93E-07
SPHK1	1.13	1.92E-10
VLDLR	-1.51	1.62E-19
MT2A	1.92	1.61E-58
ABCA5	-1.12	1.56E-10
JMJD6	1.12	1.56E-10
PBX1	-1.09	1.54E-10
NPR3	-1.05	1.52E-07
UHRF1BP1	-1.19	1.51E-18
PMAIP1	-1.18	1.43E-08
SULF1	-1.42	1.41E-06
PSPH	-1.81	1.37E-30
BCL9L	1.07	1.31E-08
SOX9	1.08	1.26E-06
F2R	-1.27	1.22E-17
MTHFD2	-1.18	1.20E-24
PIEZO2	-1.05	1.18E-12
DMD	-1.41	1.15E-06
MMP1	3.44	1.10E-176
VEGFA	-1.08	1.05E-10

Appendix Figure S5. List of Top ~100 Differentially Expressed Genes (DEGs) in HTR8/SVneo cells after UPM exposure.

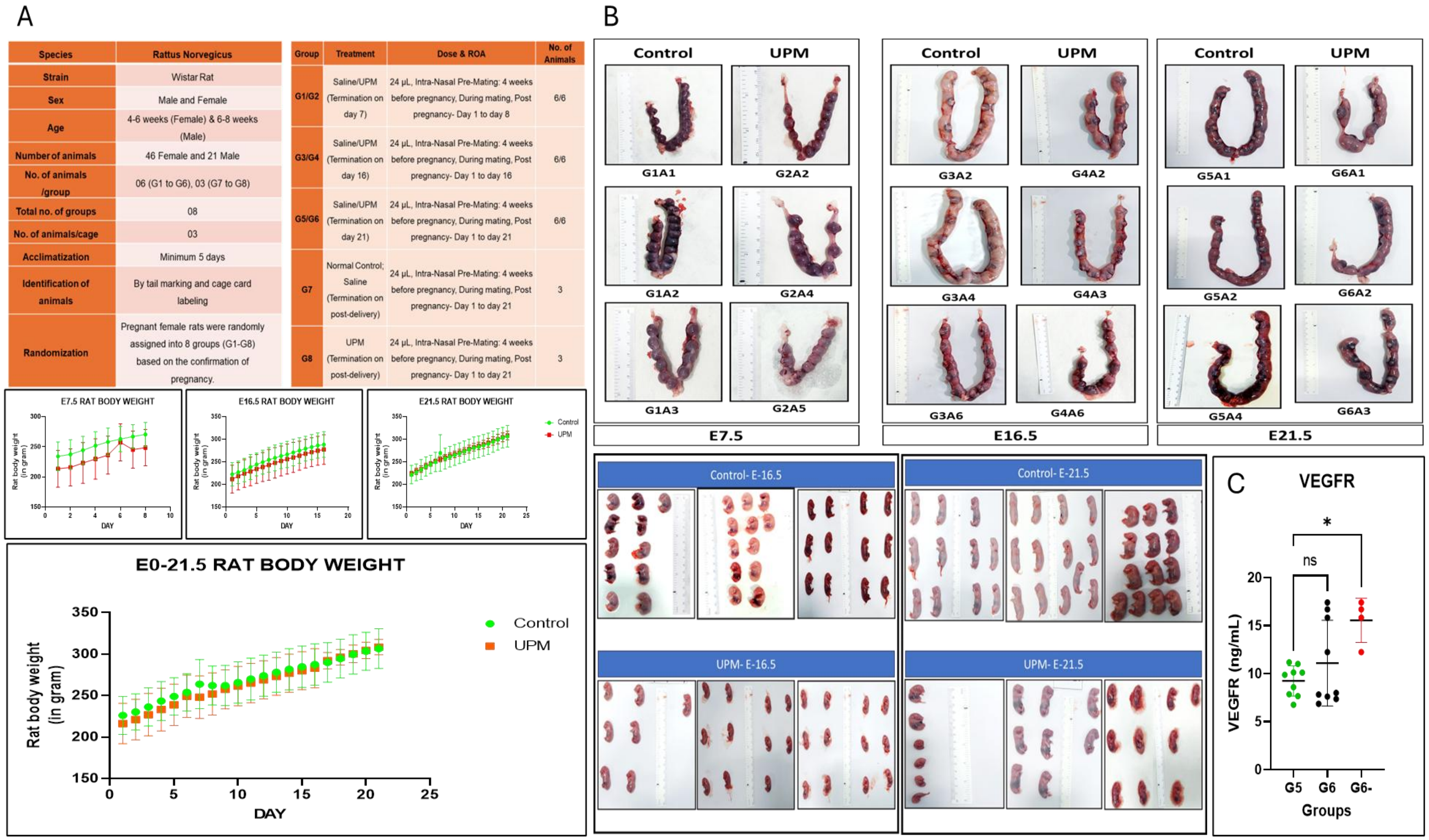

Species	Rattus Norvegicus
Strain	Wistar Rat
Sex	Male and Female
Age	4-6 weeks (Female) & 6-8 weeks (Male)
Number of animals	46 Female and 21 Male
No. of animals /group	06 (G1 to G6), 03 (G7 to G8)
Total no. of groups	08
No. of animals/cage	03
Acclimatization	Minimum 5 days
Identification of animals	By tail marking and cage card labeling
Randomization	Pregnant female rats were randomly assigned into 8 groups (G1-G8) based on the confirmation of pregnancy.

Group	Treatment	Dose & ROA	No. of Animals
G1/G2	Saline/UPM (Termination on day 7)	24 μL, Intra-Nasal Pre-Mating: 4 weeks before pregnancy, During mating, Post pregnancy- Day 1 to day 8	6/6
G3/G4	Saline/UPM (Termination on day 16)	24 μL, Intra-Nasal Pre-Mating: 4 weeks before pregnancy, During mating, Post pregnancy- Day 1 to day 16	6/6
G5/G6	Saline/UPM (Termination on day 21)	24 μL, Intra-Nasal Pre-Mating: 4 weeks before pregnancy, During mating, Post pregnancy- Day 1 to day 21	6/6
G7	Normal Control; Saline (Termination on post-delivery)	24 μL, Intra-Nasal Pre-Mating: 4 weeks before pregnancy, During mating, Post pregnancy- Day 1 to day 21	3
G8	UPM (Termination on post-delivery)	24 μL, Intra-Nasal Pre-Mating: 4 weeks before pregnancy, During mating, Post pregnancy- Day 1 to day 21	3

Appendix Figure S6: Rat experimental design and gestational outcomes following UPM exposure.

(A) Schematic representation of the experimental workflow, animal grouping, exposure paradigm, and housing conditions.

(B) Representative images of three uterine horns (top panel) and corresponding fetuses (bottom panel) collected from pregnant rats at gestational day (GD) 7.5, GD16.5, and GD21.5. Images of placental tissue and fetuses shown at GD are reused in the main manuscript Figures 4B and 4D, respectively, and are referenced here for continuity and comparative visualization across gestational stages.

(C) Quantification of soluble vascular endothelial growth factor receptor (sVEGFR) levels by ELISA in maternal serum collected at GD21.5 from control and

UPM-exposed rats (Groups G5 and G6). G6- subgroup includes data points from G6 which were higher than the control. Data are presented as individual values with group distribution.

	Exposed (764)		Non-exposed (230)	
	NBW	LBW	NBW	LBW
Age(in Years)	29.01±4.49	29.42±4.99	22.22±3.54	21.23±2.80
Gest. Age(Weeks)	38.15±1.19	36.92±1.65	38.09±1.16	36.19±1.76
Weight(K.G.)	68.40±11.49	66.28±8.95	54.83±6.99	53.22±4.54
PM2.5(μg/m3)	95.21±14.74	96.02±15.51	50	50
Baby Weight(Grams)	3015.48±330.49	2041.91±498.69	2943.4±343.48	2144.23±210.86

Appendix Figure S7. Pregnancy cohort database summarised in table.

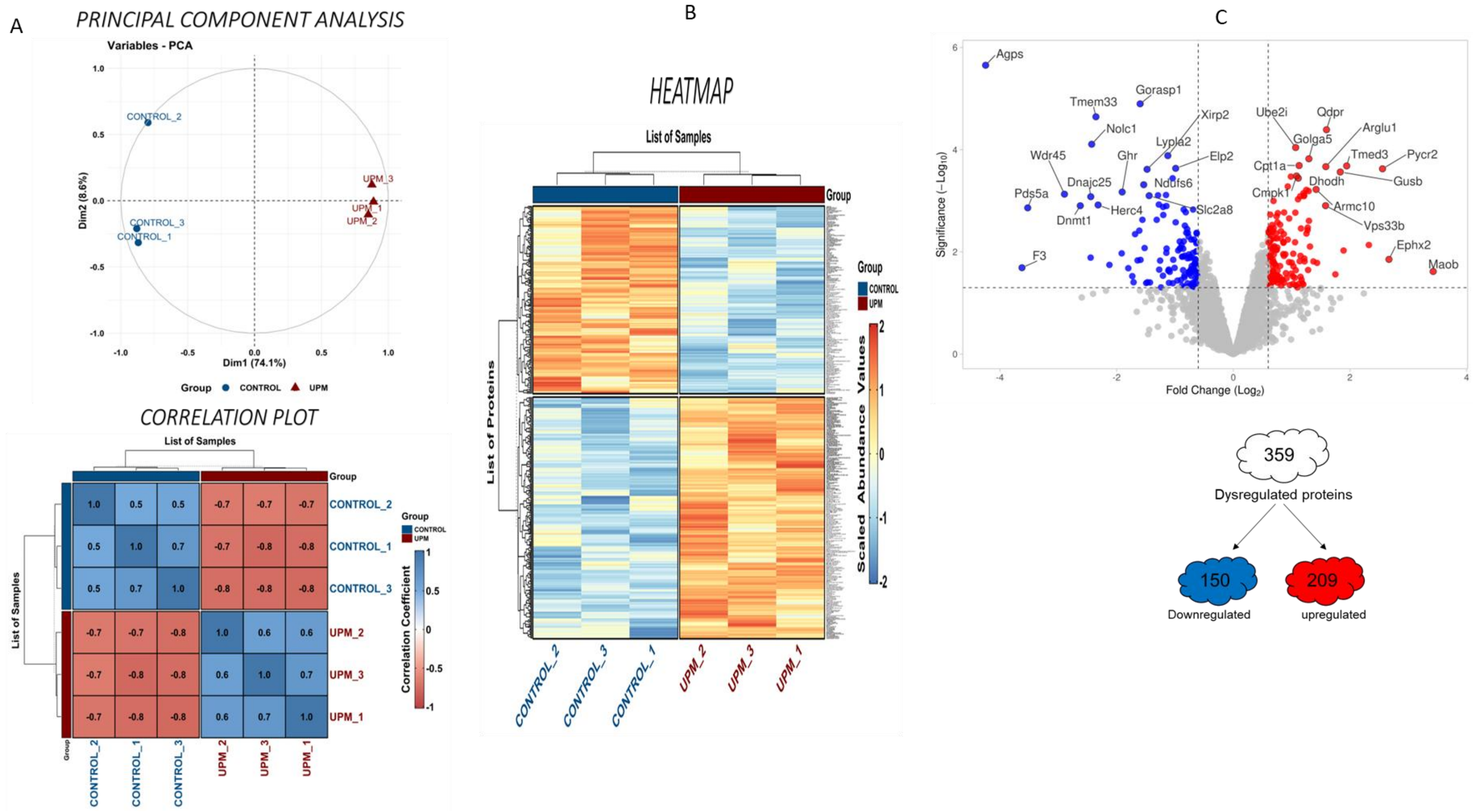

Appendix Figure S8: PCA plot (A), Heatmap (B) and volcano plot(C) for differentially expressed proteins in control vs UPM placenta at gestational Day 16.5.

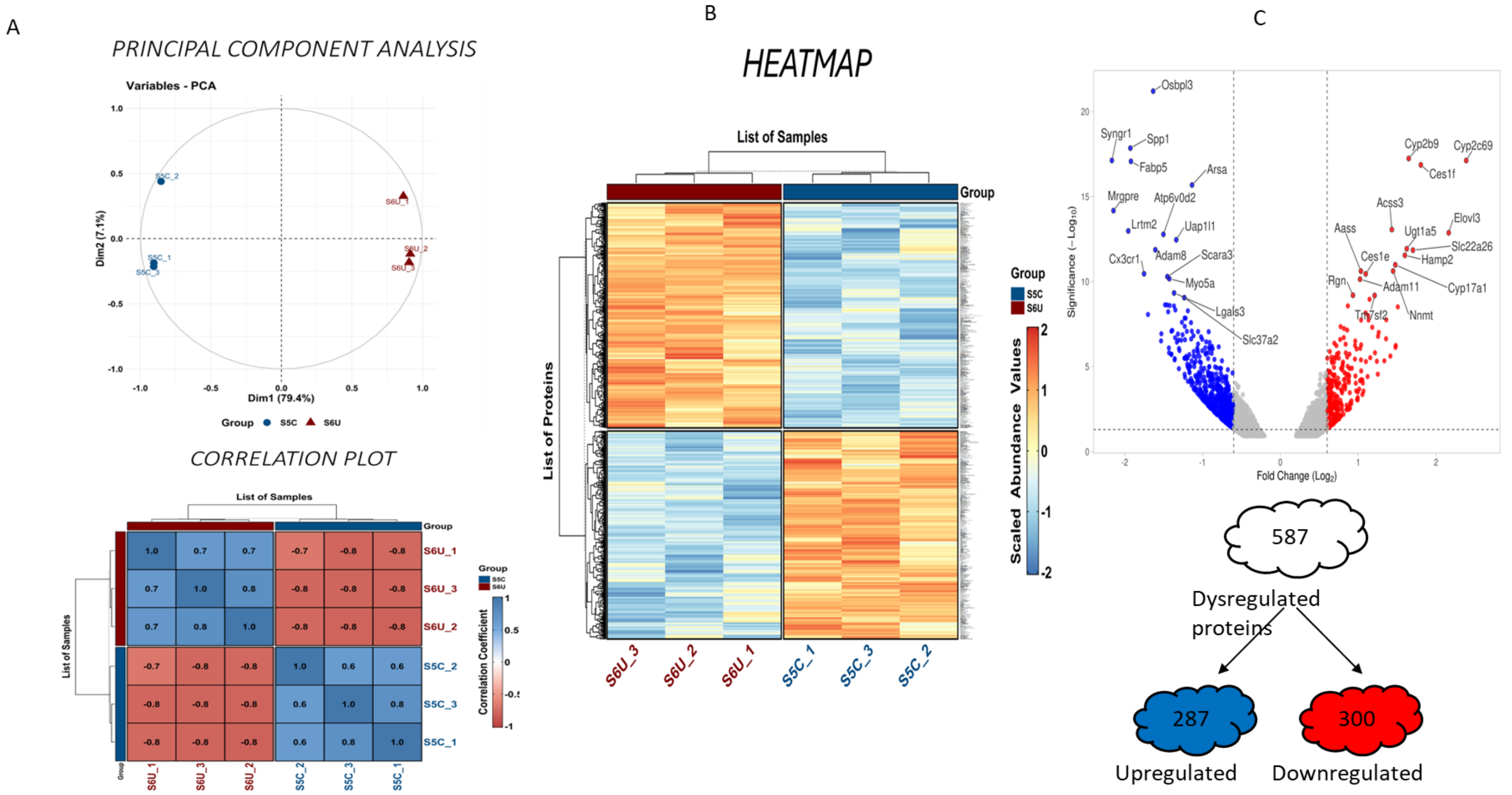

Appendix Figure S9: PCA plot (A), Heatmap (B) and volcano plot (C) for differentially expressed proteins in control vs UPM placenta at gestational Day 21.5. S5C1-S5C3: Controls; S6U1-S6U3: UPM placentas

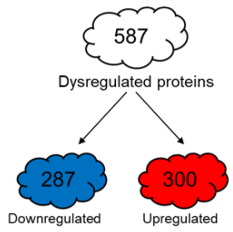

Gene	Log_2FC	Gene	Log_2FC	Gene	Log_2FC	Gene	Log_2FC	Gene	Log_2FC	Gene	Log_2FC	Gene	Log_2FC	Gene	Log_2FC	Gene	Log_2FC	Gene	Log_2FC
A1i3	-1.686	Bin1	0.960	Ctnnbl1	1.804	Fcgrt	-0.976	Hspa13	-0.804	Mrpl38	1.358	Ostc	-0.999	Prps1	0.641	Sdf4	-1.437	Strn3	1.113
A1m	-1.055	Bpnt1	0.634	Ctps2	-0.789	Fdxr	0.846	Hspa14	1.103	Mrps25	0.897	P3h1	1.025	Prss8	1.146	Sdha	0.686	Stub1	2.169
Abat	1.001	C4	-0.674	Ctsj	-1.293	Fgl1	-1.319	Hspbp1	1.294	Mrps7	1.034	Pak2	-0.854	Psmb10	0.675	Selenbp1	-0.650	Stx12	-0.602
Abhd16b	1.489	C4bpa	-1.103	Ctsq	-0.820	Fh	0.667	Hspe1	0.719	Msn	0.717	Pals1	-0.904	Psmd9	-0.674	Selenof	-0.784	Stx4	1.223
Acaa1b	1.600	C4bpb	-1.232	Cuta	-0.842	Fkbp1a	-0.838	Ier3ip1	-0.788	Mtco1	-1.677	Pard3	2.792	Psme2	-0.787	Septin5	1.214	Tagln3	-1.538
Acad9	0.618	C5	-0.642	Cyb5r1	3.395	Fkbp8	2.699	Ift56	3.548	Mtco3	0.889	Parp1	1.002	Ptdss1	0.720	Serinc1	0.616	Tanc1	-0.615
Acap2	-2.055	Ca1	-0.691	Cyb5r3	-0.945	Fmr1	1.745	Il24	1.130	Mtdh	-0.713	Pclo	3.035	Ptgfrn	-2.045	Serpina1	-0.679	Taok1	1.139
Acbd6	1.035	Cacul1	2.125	Cyp51a1	2.012	Fto	-0.604	Impa2	1.752	Mug1	-1.083	Pcyt2	0.841	Ptgr2	1.718	Serpina10	-0.878	Tax1bp3	1.452
Ace2	-3.437	Caprin1	0.902	Dad1	-0.879	Fubp1	0.613	Itpr3	1.495	Mvd	-1.210	Pdcd10	-0.733	Ptma	-1.744	Serpina3k	-1.424	Tbca	-2.322
Acot2	-1.356	Casp6	-2.421	Dcakd	-0.729	Fuca1	-0.827	Kdelr1	-1.613	Mybbp1a	0.920	Pdcl3	-1.367	Ptms	-1.814	Serpina3l	-1.475	Tecr	-0.770
Acp2	1.479	Cavin2	-2.550	Ddr1	0.880	Fundc1	-0.741	Kdelr2	-0.627	Myo1d	-0.947	Pde10a	1.000	Ptpmt1	-0.654	Serpina3n	-1.561	Tinagl1	-0.687
Actg1	-1.520	Cc2d1a	1.214	Ddx39a	0.718	G6pc3	-1.374	Khdrbs1	1.150	Myo1e	0.797	Pdia5	-1.991	Purb	1.536	Serpina6	-1.161	Tkfc	-1.254
Add2	-1.159	Cc2d1b	1.161	Dhodh	1.814	G6pdx	1.008	Kif3c	-0.701	NA	-1.084	Pdlim4	-1.015	Pus1	-0.662	Serpinb1a	-0.602	Tmed10	-0.609
Adgrf5	0.607	Ccdc127	1.602	Dlg1	-0.909	Gabarapl2	1.280	Krt15	-1.789	NA	-0.615	Pdlim7	-0.862	Pxk	-0.961	Serpind1	-0.797	Tmem165	-1.097
Afm	-0.835	Ccdc47	0.929	Dlg3	-1.603	Gale	-1.308	Krt73	0.795	NA	0.963	Pds5a	-0.936	Rab12	-0.883	Serpine1	-0.893	Tmem168	-0.860
Agrn	-1.074	Ccn2	0.611	Dnajc2	1.872	Galk2	-2.616	Krt77	1.093	NA	0.964	Pebp1	-0.737	Rab18	0.647	Serpine2	0.975	Tmod1	-1.701
Agt	-0.846	Ccnc	1.082	Dnajc5	0.610	Gatm	1.039	Lamtor1	-1.402	Naa11	0.700	Pex13	0.667	Rab1b	-0.604	Serping1	-1.543	Tmsb10	0.612
Agtrap	-1.254	Cd44	-1.033	Dnm1l	-0.638	Get1	-1.381	Lamtor3	-1.147	Naa35	1.242	Pex6	0.730	Rabep1	-3.093	Setd3	0.657	Tmt1b	-1.183
Ahsg	-0.842	Cd93	1.307	Drap1	0.990	Gfpt2	1.039	Lap3	0.622	Nampt	-0.812	Pfdn2	0.664	Rai14	-0.720	Sfxn3	0.888	Tollip	-0.750
Aifm1	0.741	Cdc5l	-0.965	Dusp7	-1.767	Ggps1	-1.729	Lcmt1	-1.103	Nasp	0.980	Pfkm	1.102	Ralbp1	1.009	Sh3bgrl3	0.608	Tomm22	1.606
Aip	1.582	Cdk1	1.649	Dut	1.876	Ggt7	0.881	Lgals5	-1.097	Ncbp2	0.680	Pgap1	-2.867	Rbp4	-0.694	Sh3gl1	0.729	Tor1a	0.709
Ak1	-1.664	Cdk2	2.639	Dync1li1	-1.057	Git2	0.809	Lifr	-0.663	Nceh1	-1.966	Pggt1b	0.950	Rcn2	-0.965	Siae	0.807	Tor3a	0.620
Akap2	0.685	Cdk7	0.675	Ech1	0.729	Glrx	-0.998	Litaf	1.297	Ncl	0.725	Pi4ka	-1.588	Rexo2	-1.345	Slc12a9	-1.461	Tpm2	0.665
Akr7a2	-1.294	Cebpb	-0.691	Echdc3	-0.690	Glud1	0.734	Lmbrd1	-1.982	Ndufa10	1.321	Pik3r4	-0.955	Rgcc	-0.772	Slc13a3	-0.669	Tpm3	0.667
Akt1	0.776	Celf1	1.171	Ednrb	-1.823	Glul	1.987	Lmnb1	0.616	Ndufa5	1.009	Pip4k2c	0.777	Rhag	1.654	Slc25a12	0.669	Tpt1	0.687
Aldh1l1	-1.386	Cep55	-1.431	Ehd2	-1.233	Glyr1	-1.120	Lonp2	1.019	Ndufv2	0.666	Pip4p1	-1.200	Rhd	-1.776	Slc25a22	-1.180	Trip13	0.884
Aldoc	-1.400	Cep57	2.393	Eif2b5	1.173	Gng5	1.065	Loxl2	-0.703	Necap1	1.056	Pkn1	1.419	Rmdn3	-1.894	Slc25a25	0.861	Ttc39c	1.386
Ambp	-1.468	Ces1d	2.446	Eif3c	0.619	Gosr2	-0.969	Lrp2	-1.404	Nfyc	-1.090	Plbd1	0.761	Rnf114	0.700	Slc30a1	0.994	Ttc5	-0.773
Ank3	-0.725	Cfi	-0.893	Eif3e	0.942	Gpd2	0.866	Lrpap1	-1.622	Nid1	-1.312	Plbd2	-1.042	Rnpep	0.830	Slc35b1	-0.965	Ttr	-0.808
Apcs	-0.923	Chchd6	0.864	Eif3g	1.140	Gpr107	0.641	Lrprc	0.654	Nid2	-0.664	Plek	-1.046	Rock1	0.770	Slc35e1	-1.368	Ubxn1	1.114
Apoa1	-1.425	Chd5	0.721	Eif4h	0.880	Grb10	-1.268	Lrrfip2	-0.824	Nmes1	1.851	Plet1	1.428	Rpl10a	0.732	Slc38a4	-2.795	Uqcrq	0.896
Apoa4	-0.760	Chmp4c	0.978	Elob	1.082	Grb2	0.758	Lxn	2.084	Nmral1	-0.821	Plg	-0.834	Rpl11	0.670	Slc39a7	-0.702	Usp4	1.119
Apob	-0.622	Chmp5	1.167	Elp1	2.739	Grik5	1.193	Lyn	0.734	Nolc1	1.054	Plrg1	0.973	Rpl12	0.624	Slc3a2	-0.951	Utp15	0.832
Apoc4	-1.543	Chrna5	1.375	Emb	-1.212	Grwd1	0.776	Lztfl1	1.206	Npl	-1.395	Pmpca	-0.895	Rpl13a	0.615	Slc41a3	-0.689	Vac14	-0.780
App	-1.497	Ciapin1	-0.957	Emc2	0.734	Gstp1	-0.643	Magoh	0.614	Npm1	0.710	Pmpcb	1.104	Rpl23a	1.607	Slc6a12	0.947	Vcpip1	0.767
Aqp1	-0.795	Cit	0.697	Emd	1.459	Gstz1	-1.980	Map1	-1.231	Nptn	1.015	Pnp	0.756	Rpl28	0.895	Slc7a5	-1.001	Vdac2	1.076
Arf2	-2.140	Clpb	2.013	Enpp3	-0.633	Gtf2a2	0.661	Map1s	-0.626	Nr0b1	2.104	Pola2	1.093	Rpl32	0.752	Slc7a8	-1.214	Vezt	0.836
Arf6	-0.758	Clu	-1.216	Entpd1	1.387	Gtf2b	-1.723	Map2k2	-1.544	Nradd	0.681	Polr1b	0.777	Rpl34	0.612	Slco2a1	-1.207	Vps25	-0.851
Arfgap2	0.686	Cnn3	-0.999	Entpd2	-1.970	Gtf2f1	1.261	Map6	-0.883	Nt5c2	0.734	Polr2e	0.853	Rpl35a	0.669	Smad2	1.604	Vps4a	1.588
Arfrp1	-0.793	Cnnm4	1.778	Epb41l1	0.976	Gusb	0.790	Mapre2	-1.058	Ntaq1	-0.723	Pomgnt1	0.780	Rpl37	1.134	Smarca4	0.825	Wbp2	0.767
Arhgef2	0.605	Cnot10	-0.740	Epcam	-0.708	H1-1	1.157	Mcm6	2.333	Nubp1	1.718	Ppat	1.070	Rpl38	-0.739	Smim7	0.920	Wdr18	0.901
Arl1	-0.716	Cnp	-1.358	Ephx1	-0.656	H1-4	0.830	Mcm9	0.839	Nucb2	-0.953	Ppef1	-1.202	Rpl6	0.787	Smpd2	-1.445	Wdr35	4.657
Arl3	0.904	Cog7	0.629	Epn1	-1.264	H1-5	0.861	Mcts1	-1.157	Nucks1	1.331	Ppm1a	-0.886	Rplp1	-1.087	Smu1	-0.660	Wdr44	1.482
Arl6ip5	-0.734	Col5a1	2.179	Epn3	-2.166	H2ac25	2.382	Memo1	0.950	Nudt2	0.711	Ppp1r2	-2.780	Rps10	0.894	Snapin	-1.036	Wdr5	-1.238
Armc9	-1.442	Coq6	0.904	Erp29	-0.785	Hdgf	0.657	Mest	-0.646	Nudt21	0.669	Ppp2cb	0.790	Rps11	0.682	Sort1	-1.240	Wdr7	0.609
Arrb1	-1.944	Coro1a	1.864	Esam	1.123	Hdgfl3	-1.366	Mfge8	-1.908	Nudt3	-0.874	Ppp2r2d	-1.212	Rps14	0.649	Spryd4	1.719	Wdr77	-1.643
Arvcf	-0.617	Cox7a2	0.862	Esd	-0.865	Herc4	-0.743	Mgat2	-0.851	Nudt5	0.663	Ppp3r1	-1.781	Rps15	1.293	Sptbn2	0.603	Yap1	-1.193
Atp1b3	-0.799	Cox7b	0.626	Exoc8	0.966	Hexa	-1.390	Mgat5	-1.115	Numb	-1.594	Ppp6c	1.804	Rps15a	1.090	Sptlc2	3.207	Ybx3	0.965
Atp2a3	2.910	Cox7c	1.066	F10	-1.353	Hint1	-0.802	Mitf	0.739	Nup107	0.651	Prdx6	-0.629	Rps19	0.771	Srgap2	-0.606	Yipf4	-1.039
Atp5if1	1.446	Cpb2	-1.109	F2	-1.429	Hmgb1	0.602	Mlycd	-0.693	Nup153	-2.787	Prkacb	-1.038	Rps6ka1	-1.677	Srsf2	1.123	Yme1l1	0.621
Atp6v1f	-0.620	Cpd	2.057	F9	-1.794	Hmgb2	0.763	Mogs	-0.661	Nup50	0.620	Prkcz	-0.712	Rras	-1.173	Srsf5	1.201	Yod1	-0.645
Avil	-2.336	Cps1	1.094	Fabp3	-0.634	Hmgcs2	-0.732	Mpc2	-1.027	Nup54	0.750	Prl3d4	-1.782	Rrs1	0.977	Ssbp1	0.802	Ythdc1	3.378
Azgp1	-1.064	Cpsf4	-0.601	Faf2	-1.470	Hnrnpdl	0.754	Mpp3	-2.037	Nup58	-1.104	Prl4a1	-1.413	Ryr1	-1.282	Ssr1	-0.794	Ywhag	0.604
B3gnt7	-1.956	Cpt2	0.681	Fah	-0.696	Hnrnpm	0.766	Mprip	-1.287	Nup62	-0.818	Prl8a4	-2.300	S100a4	1.250	Ssrp1	0.706	Ywhah	0.646
B4galt6	0.697	Crp	-1.069	Fahd2	-0.956	Hpgd	-1.029	Mrc2	-1.453	Ocm	4.004	Prl8a5	-1.305	S100a6	0.955	Ssu72	1.239	Zc3h15	0.860
Babam1	0.907	Csf1r	-1.852	Fam210a	0.715	Hpn	1.825	Mrgprg	1.469	Ogfod3	1.149	Prl8a7	-2.735	S100a9	1.102	St13	0.789	Zwint	0.617
Bcam	-1.117	Csk	1.263	Fancd2	-1.111	Hsbp1	1.396	Mrpl16	0.864	Ogt	1.253	Prnp	1.180	Scarb2	1.397	St6gal1	-1.174		
Bcat1	1.126	Csrp1	-0.916	Farp1	0.960	Hsd17b12	-0.692	Mrpl22	1.357	Orai1	-1.825	Pros1	-0.849	Scfd1	0.933	Stbd1	1.007		
Bdh1	-0.741	Cst3	-1.391	Fau	2.045	Hsdl2	-0.671	Mrpl23	-0.889	Osgep	0.875	Prpf19	1.219	Scpep1	-1.059	Stim1	-1.184		

-3.436 0 4.656

Appendix Figure S10. Heat Map for Differentially expressed proteins in control vs UPM placenta from Gestational Day 21.5

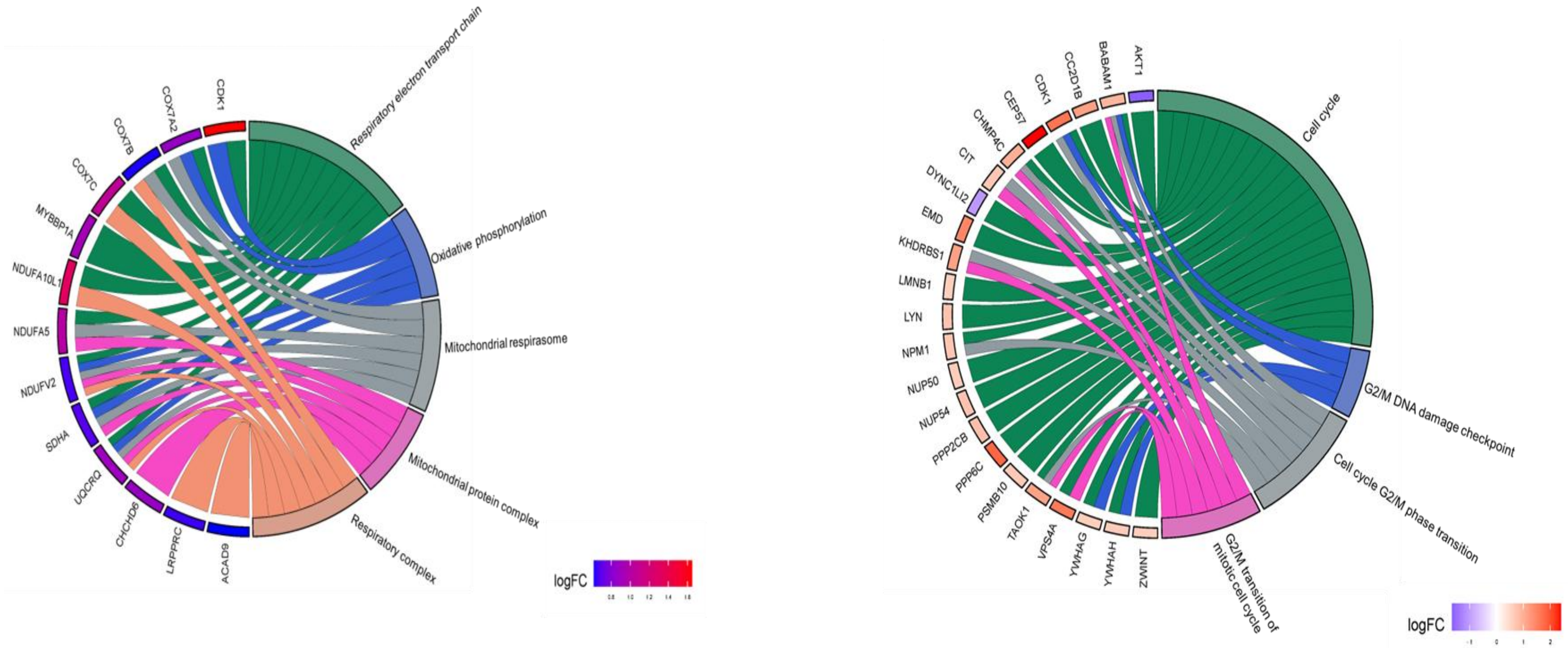

Appendix Figure S11: Chord-plot for proteins altered in various critical processes such as cell cycle and DNA repair.

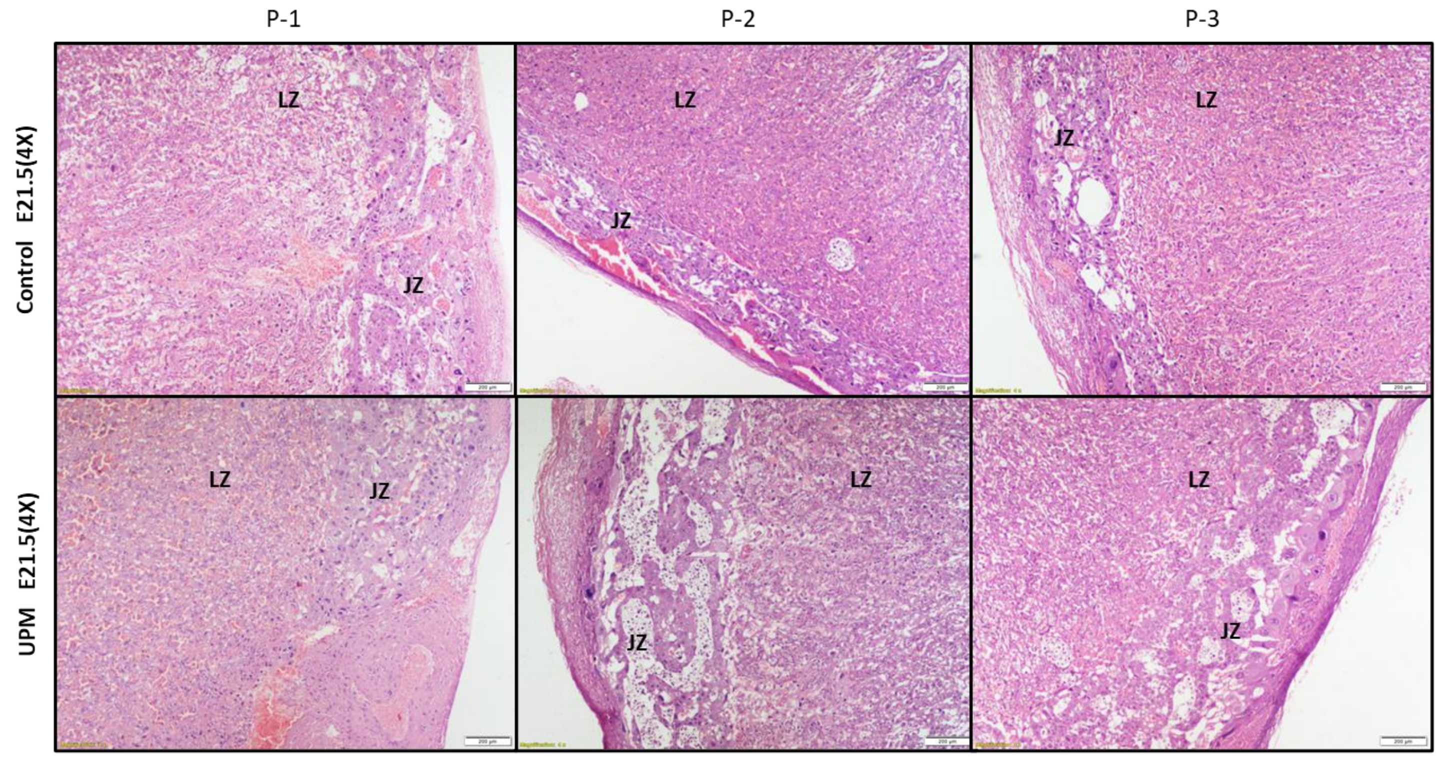

Feature	Control P1	Control P2	Control P3	UPM-Treated P1	UPM-Treated P2	UPM-Treated P3
Zonal Clarity	Clear demarcation between LZ and JZ	Clear demarcation between LZ and JZ	Clear demarcation between LZ and JZ	Zonal architecture slightly disrupted	JZ appears disorganized and less cellular	Disruption of LZ-JZ boundary
Cellular Morphology	Normal; dense nuclei; Organized	Normal; dense nuclei; Organized	Normal; dense nuclei; Organized	Cellular vacuolation; decreased nuclear staining	Swollen, nuclear pyknosis	Degenerating
Vascularization	Prominent in LZ	Prominent in LZ	Prominent in LZ	Reduced capillary density	some collapsed	Capillaries sparse and distorted; signs of ischemia
Cellular Integrity	High	High	High	Moderate; early signs of necrosis	Reduced integrity with fragmentation	Poor; areas of necrosis
Other Features	Healthy stroma	Normal glycogen cells in JZ	Minimal tissue stress	Immune cell infiltration	Junctional collapse	Hemorrhagic areas

LZ: Labyrinth zone
JZ: Junctional zone
P1-3: Placenta

Appendix Figure S12.H&E staining of E21.5 tissues

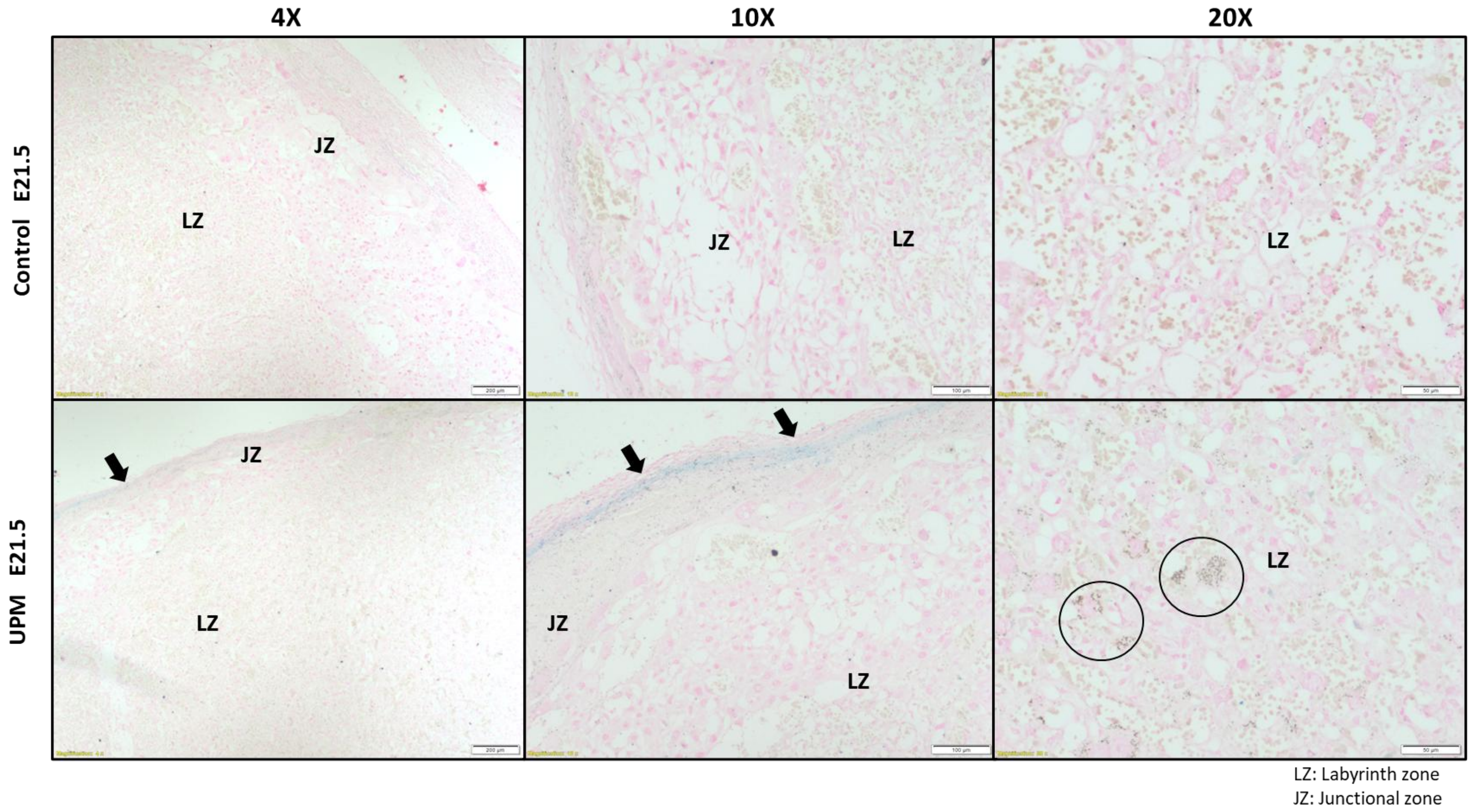

Appendix Figure S13. Iron staining of E21.5 tissues. Arrow marks showing the Prussian blue color for Iron staining in the tissue and circled boundary indicates the signs of lipofuscins.

Genus - Relative Abundance (Top 10)

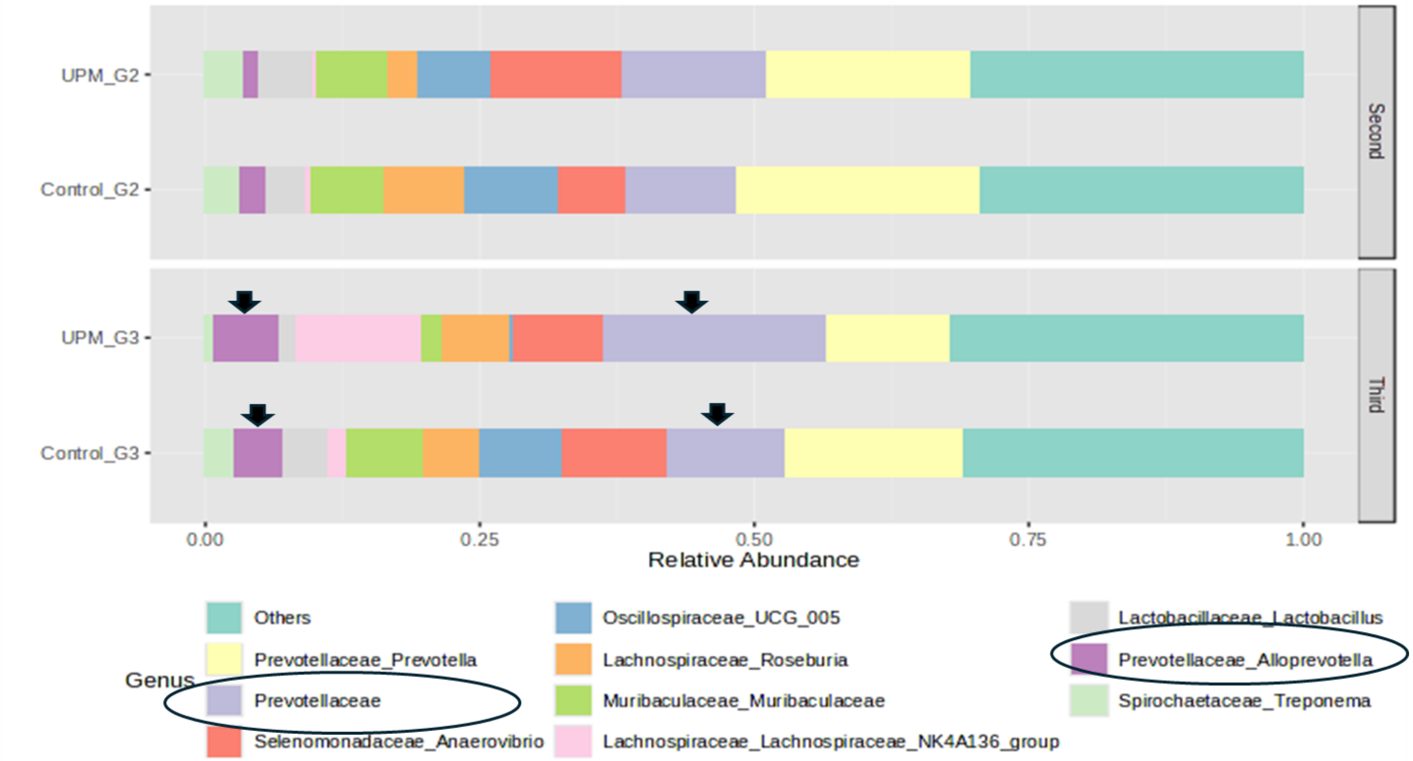

Appendix Figure S14. Microbiome analysis of stool samples from control and UPM treated dams. G2: GD16.5, G3: GD21.5

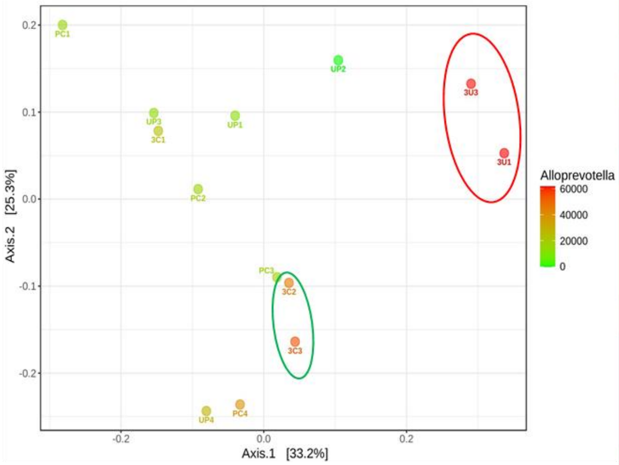

Beta Diversity

- Red Circle indicating the stool samples from UPM treated rats at Day21.5 showed high abundance for Alloprevotella

PC: Control (GD16.5)
UP: UPM treated (GD16.5)
3C: Control (GD21.5-Green Circle)
3U: UPM treated (GD21.5-Red Circle)

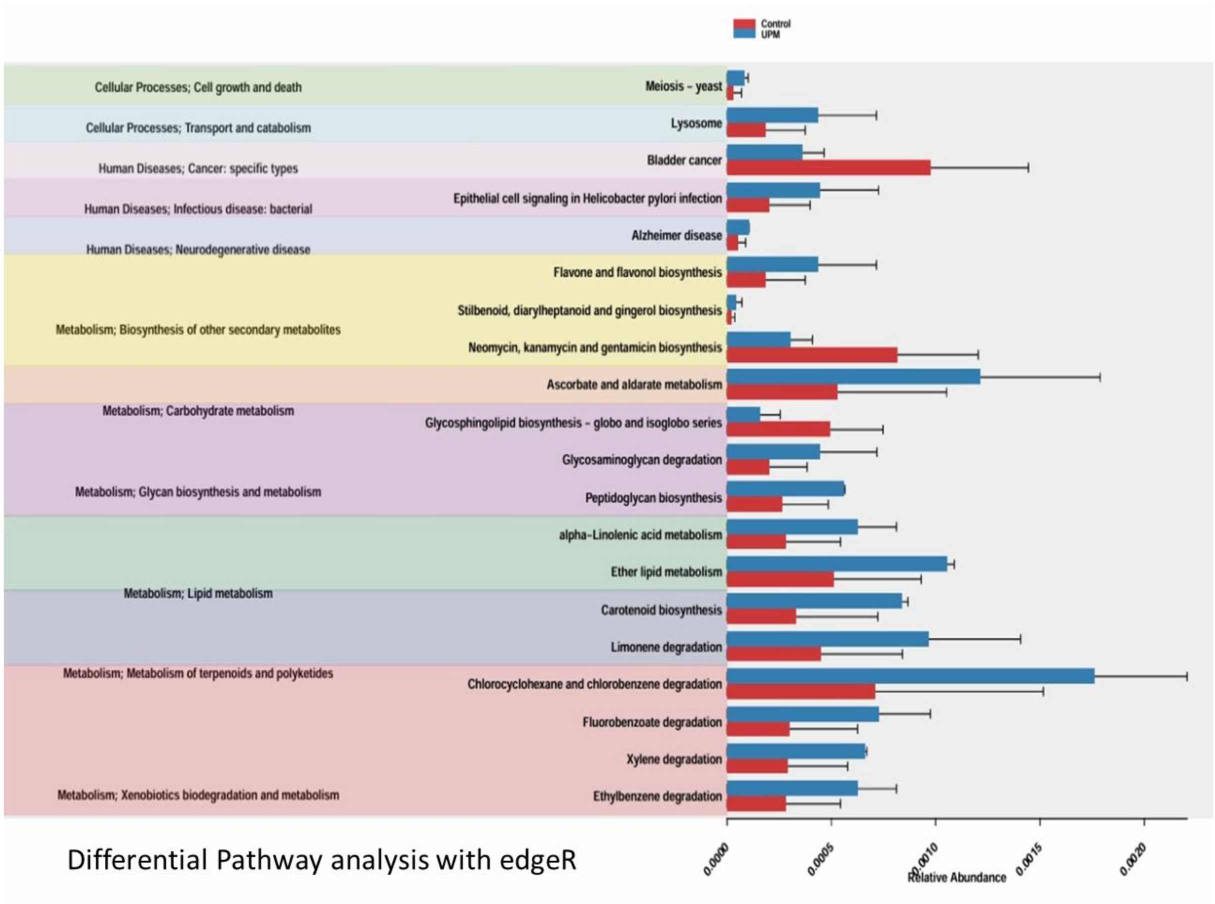

Appendix Figure S15. Microbiome analysis of stool samples from control and UPM treated dams.

GEO ID	IGFBP3	Pvalue	STATUS	STAT1	STATUS	Pvalue
GSE203346	-0.379	ns	↓	-0.232	↓	ns
GSE216484	-0.276	ns	↓	-0.050	↓	ns
GSE216484	-1.059	****	↓	0.092	↑	ns
GSE216484	-3.698	****	↓	1.199	↑	***
GSE237795	-0.436	****	↓	0.230	↑	****
GSE261095	-0.585	****	↓	0.106	↑	ns
GSE220756	-0.227	ns	↓	0.288	↑	ns
GSE220756	-0.512	ns	↓	2.272	↑	****
GSE30186	-1.032	**	↓	-0.350	↓	ns
GSE44711	-0.510	ns	↓	0.836	↑	*

A

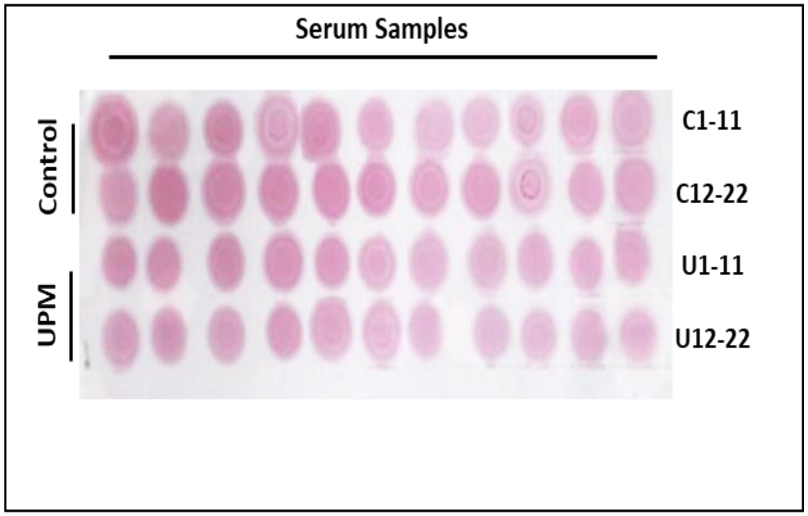

B

Appendix Figure S16. (A) Reciprocal relationship between IGFBP3 and STAT1 expression: The table presents the log_2 fold changes in IGFBP3 and STAT1 expression from various public mRNA expression datasets. The 'Status' column indicates upregulation (↑) or downregulation (↓) of each gene.
(B) Ponceau S staining to show equal loading of protein in all the spots. C1-22: Serum of 22 control Pups; U1-22: Serum of 22 UPM pups

Appendix Table S1: List of Antibodies used

Antibody Name	Source	Dilution	Molecular Weight
**MMP2**	Abclonal-Rabbit-A19080	1:1000 WB	72 & 64KDa
**MMP9**	Abclonal-Rabbit- A0289	1:1000 WB	92KDa
**TIMP1**	Abclonal-Rabbit- A1389	1:1000 WB	26KDa
**TIMP2**	Abclonal-Rabbit- A1558	1:1000 WB	22KDa
**XBP1s**	Abclonal-Rabbit- A1731	1:1000 WB<br>1:250 IF	60KDa
**NRF2**	Abcam-Rabbit- ab62352	1:1500 WB	95KDa
**IRE1A**	CST-Rabbit #3294	1:1000 WB	110KDa
**Bip**	Abclonal-Rabbit- A11366	1:1000 WB	78KDa
**NFkB**	Abcam-Rabbit- ab32536	1:1000 WB	65KDa
**Syncytin1**	ERVW-1, Immunotag-ITAB91578. Rabbit	1:1000 WB	55-59KDa
**CHOP**	CST-Mouse- #2895	1:500 WB	27KDa
**IGFBP3 (Human)**	CST-Rabbit #25864	1:1000 WB	40KDa
**IGFBP3 (Rat)**	Elabscience-Rabbit-E-AB-91410	1:1000 WB<br>1:250 IHC	40KDa
**GAPDH**	Affinity-Rabbit-AF7021	1:1500 WB	37KDa
**Anti-Rabbit-HRP**	CST-7074S	1:4000-5000 WB	Not Applicable
**Anti-Mouse-HRP**	CST-7076	1:4000-5000 WB	

**Anti-Rabbit-Alexa Fluor 594**	Invitrogen- A11029	1:750 IF	
**Anti-Mouse-Alexa Fluor 488**	Invitrogen- A11012	1:750 IF	
