# Supplementary figures and images for: IGFBP3 repression driven by inflammation links air pollution to placental and developmental defects
Source: EMBO Mol Med. 2026 Mar 24;18(5):1648–78. doi: 10.1038/s44321-026-00403-x (PMC13179339; doi:10.1038/s44321-026-00403-x)

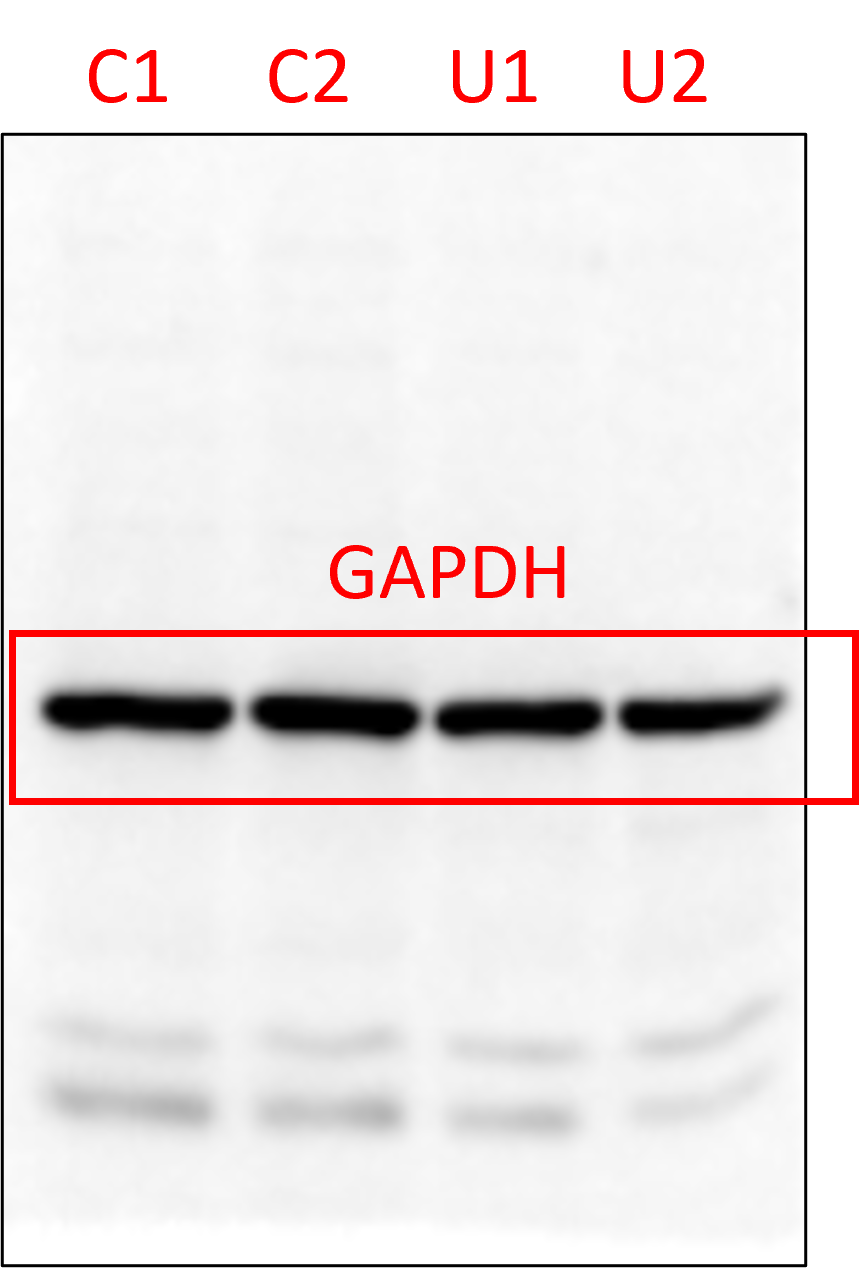

Supplement: Supplementary file 3 — Source data Fig. 1 [file 44321_2026_403_MOESM3_ESM.zip › A/Fig.1 GAPDH(mmp's blot).tif]

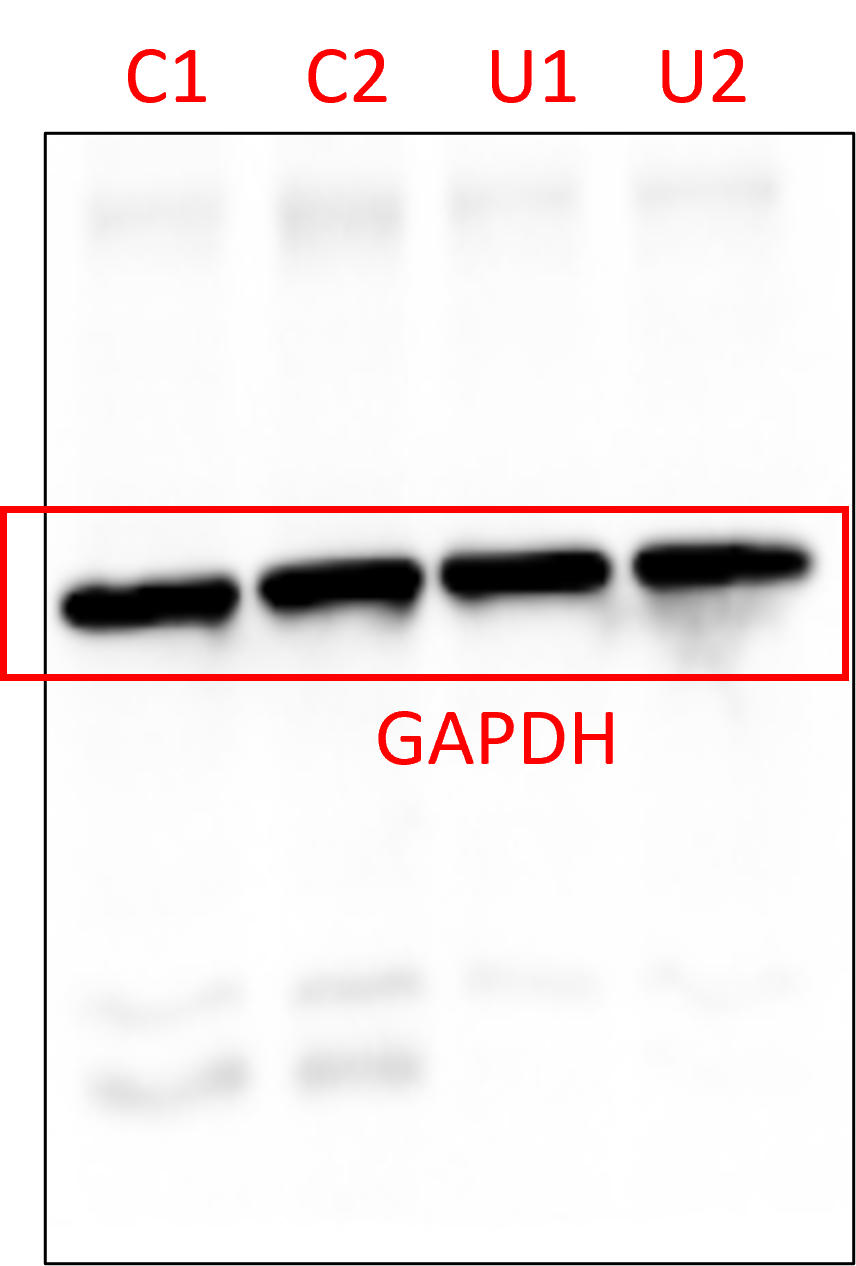

Supplement: Supplementary file 3 — Source data Fig. 1 [file 44321_2026_403_MOESM3_ESM.zip › A/Fig.1 GAPDH.tif]

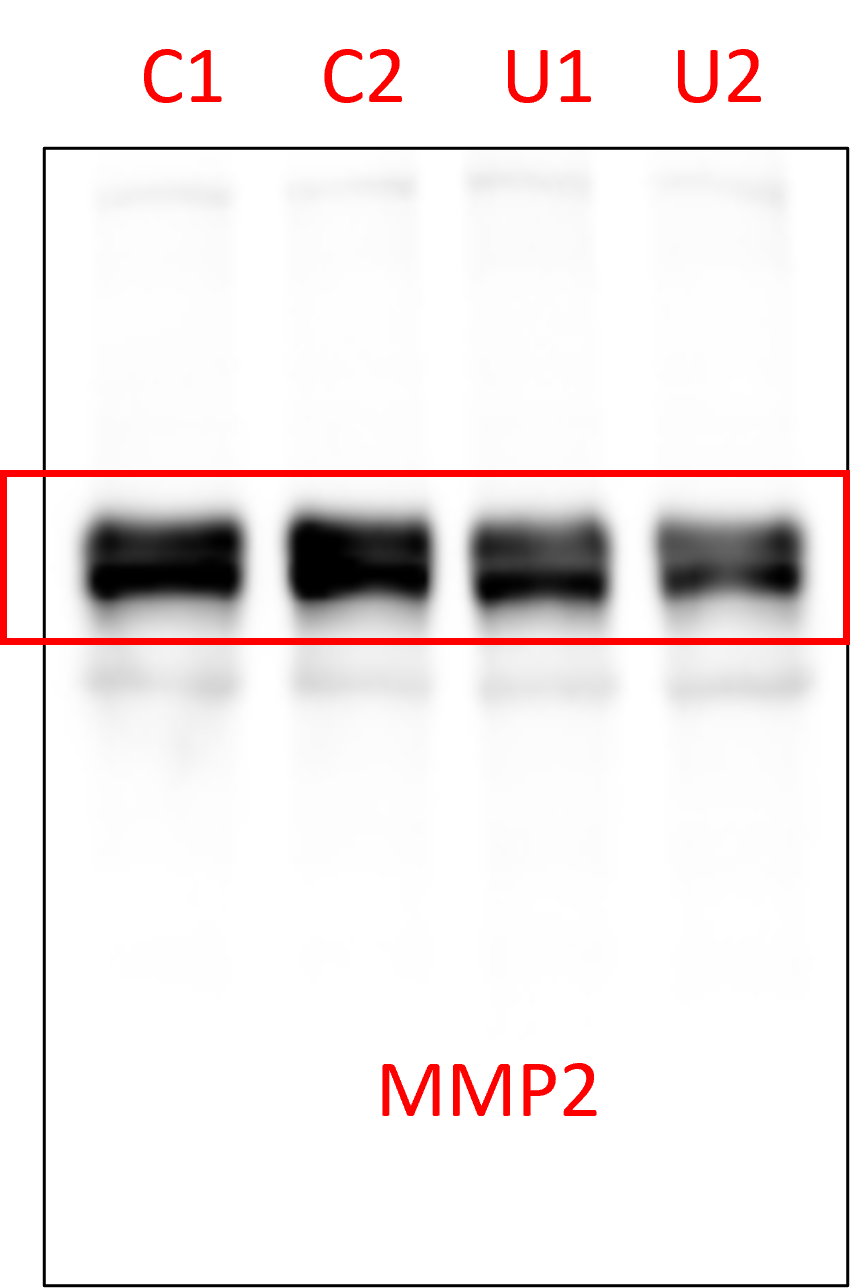

Supplement: Supplementary file 3 — Source data Fig. 1 [file 44321_2026_403_MOESM3_ESM.zip › A/Fig.1 MMP2.tif]

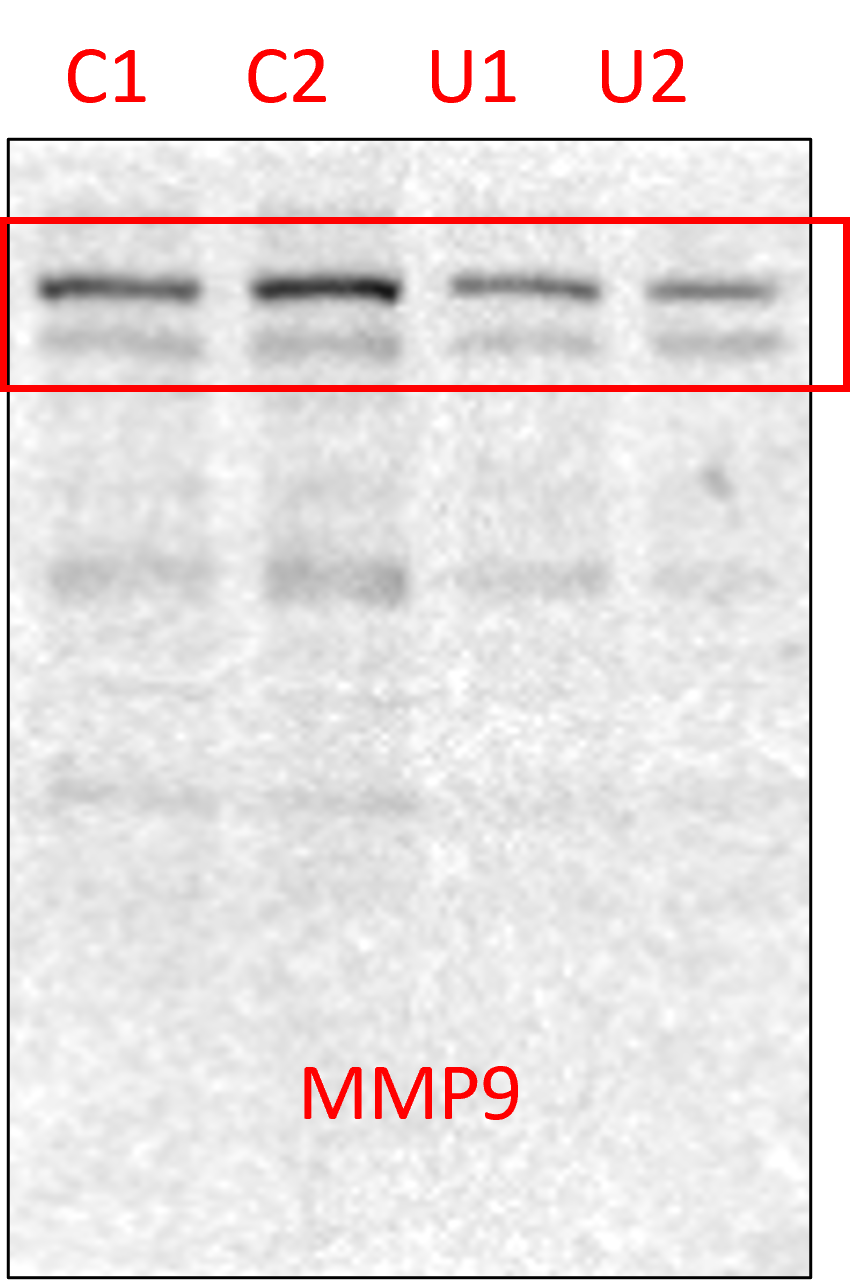

Supplement: Supplementary file 3 — Source data Fig. 1 [file 44321_2026_403_MOESM3_ESM.zip › A/Fig.1 MMP9.tif]

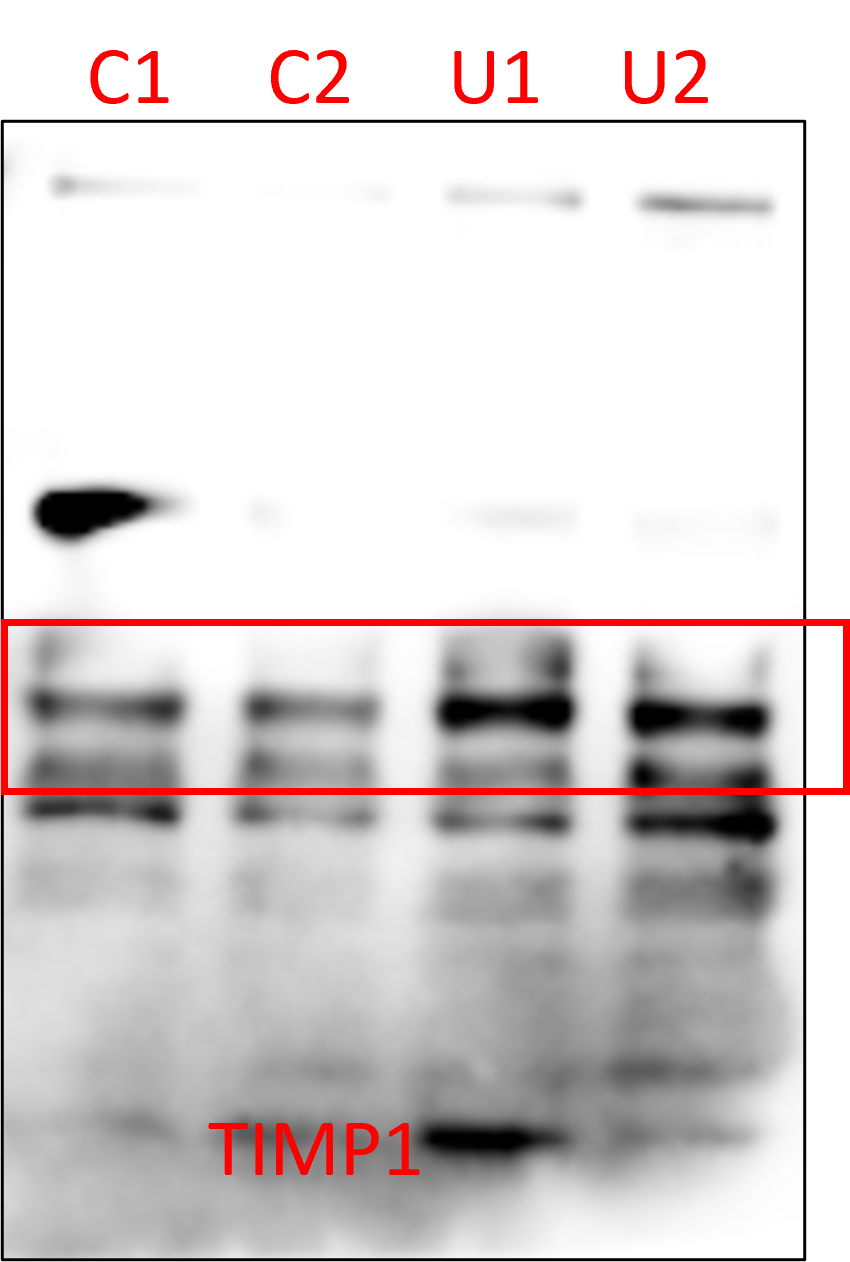

Supplement: Supplementary file 3 — Source data Fig. 1 [file 44321_2026_403_MOESM3_ESM.zip › A/Fig.1 TIMP1.tif]

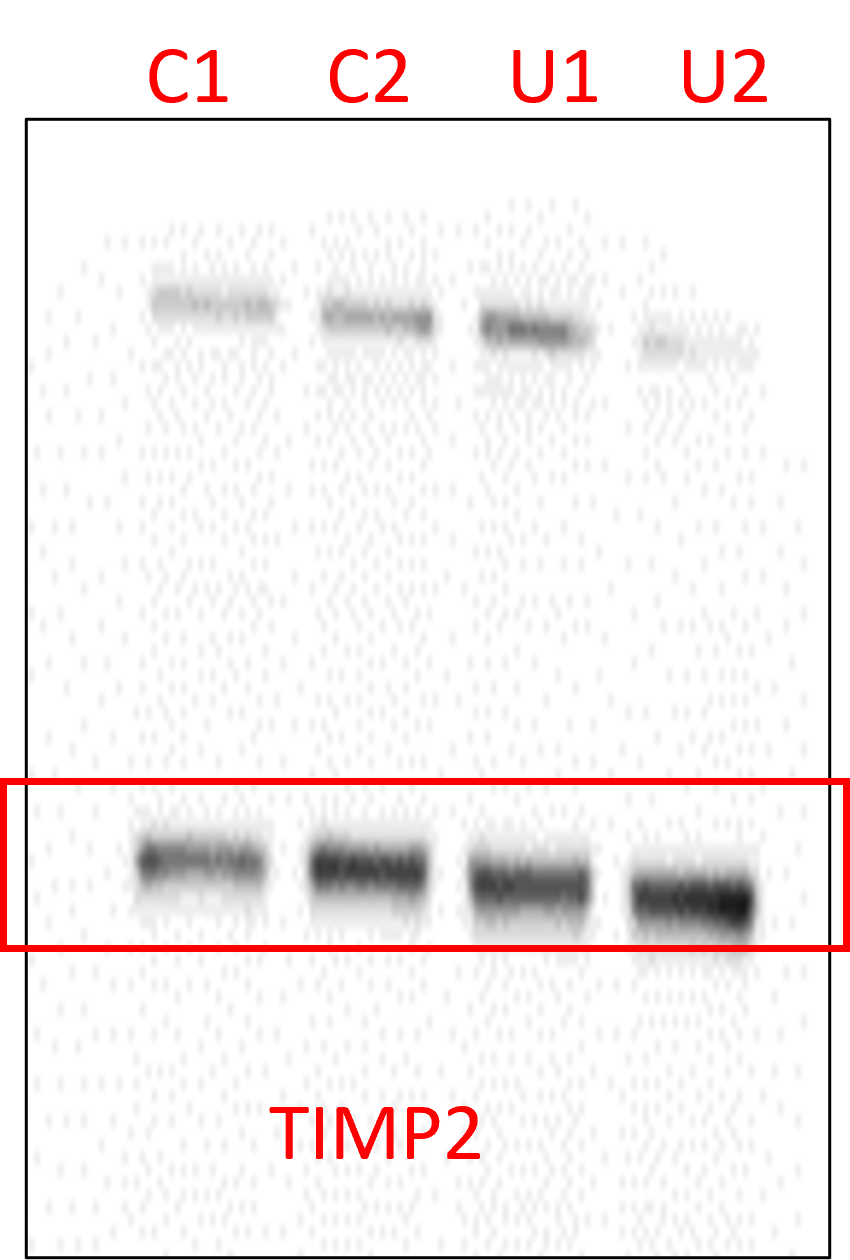

Supplement: Supplementary file 3 — Source data Fig. 1 [file 44321_2026_403_MOESM3_ESM.zip › A/Fig.1 TIMP2.tif]

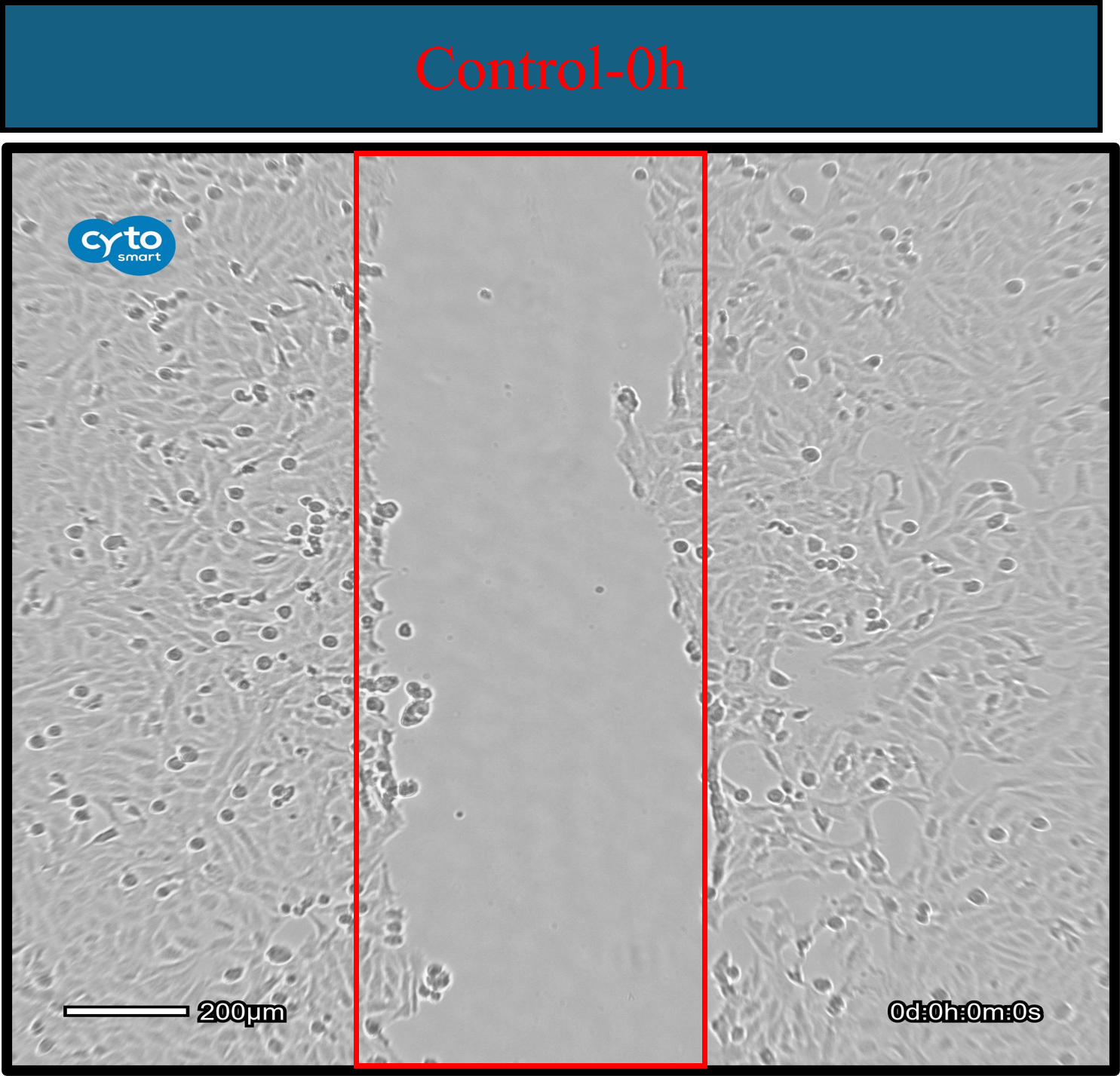

Supplement: Supplementary file 3 — Source data Fig. 1 [file 44321_2026_403_MOESM3_ESM.zip › B/ctrl-0h.tif]

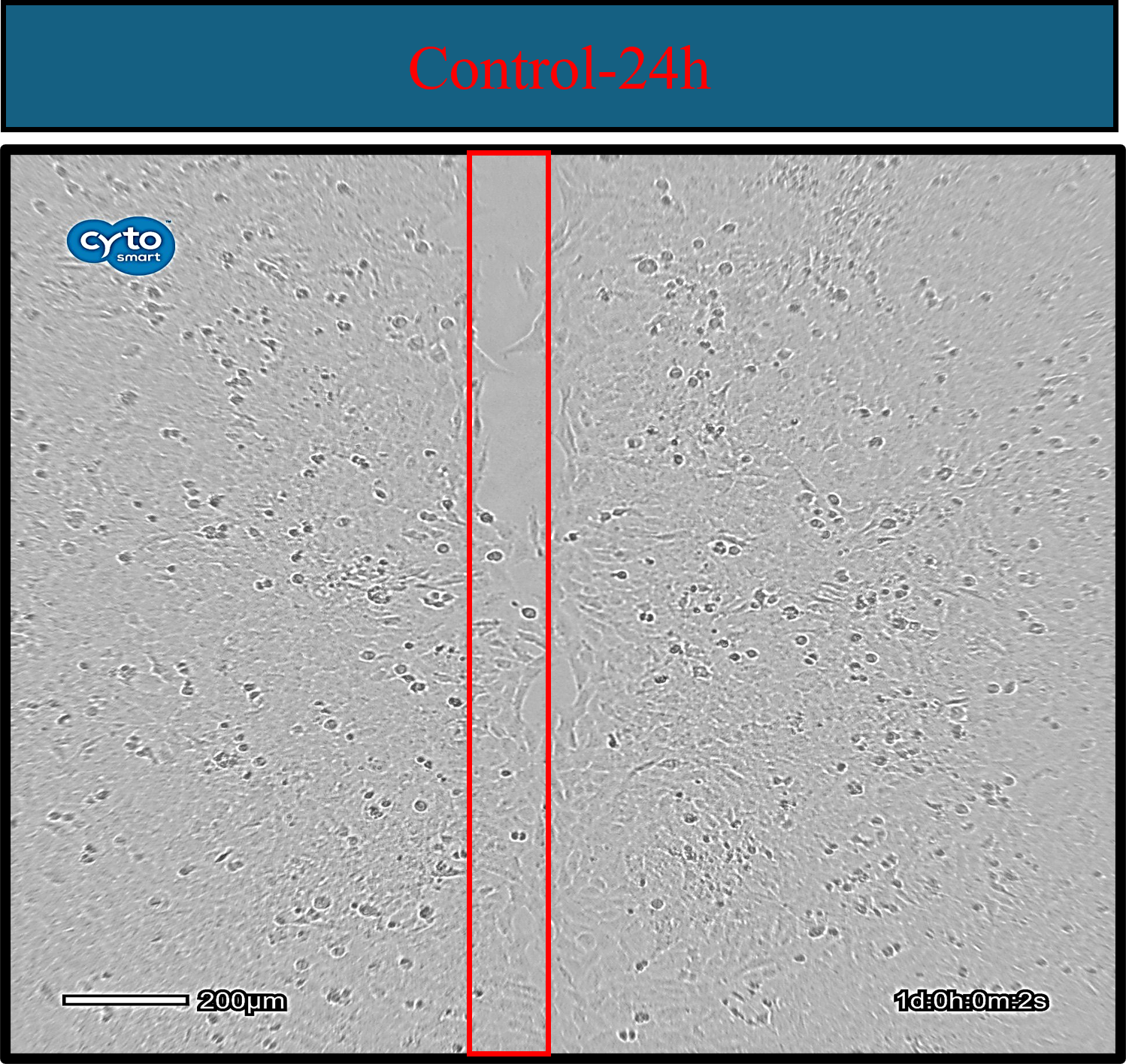

Supplement: Supplementary file 3 — Source data Fig. 1 [file 44321_2026_403_MOESM3_ESM.zip › B/ctrl-24h.tif]

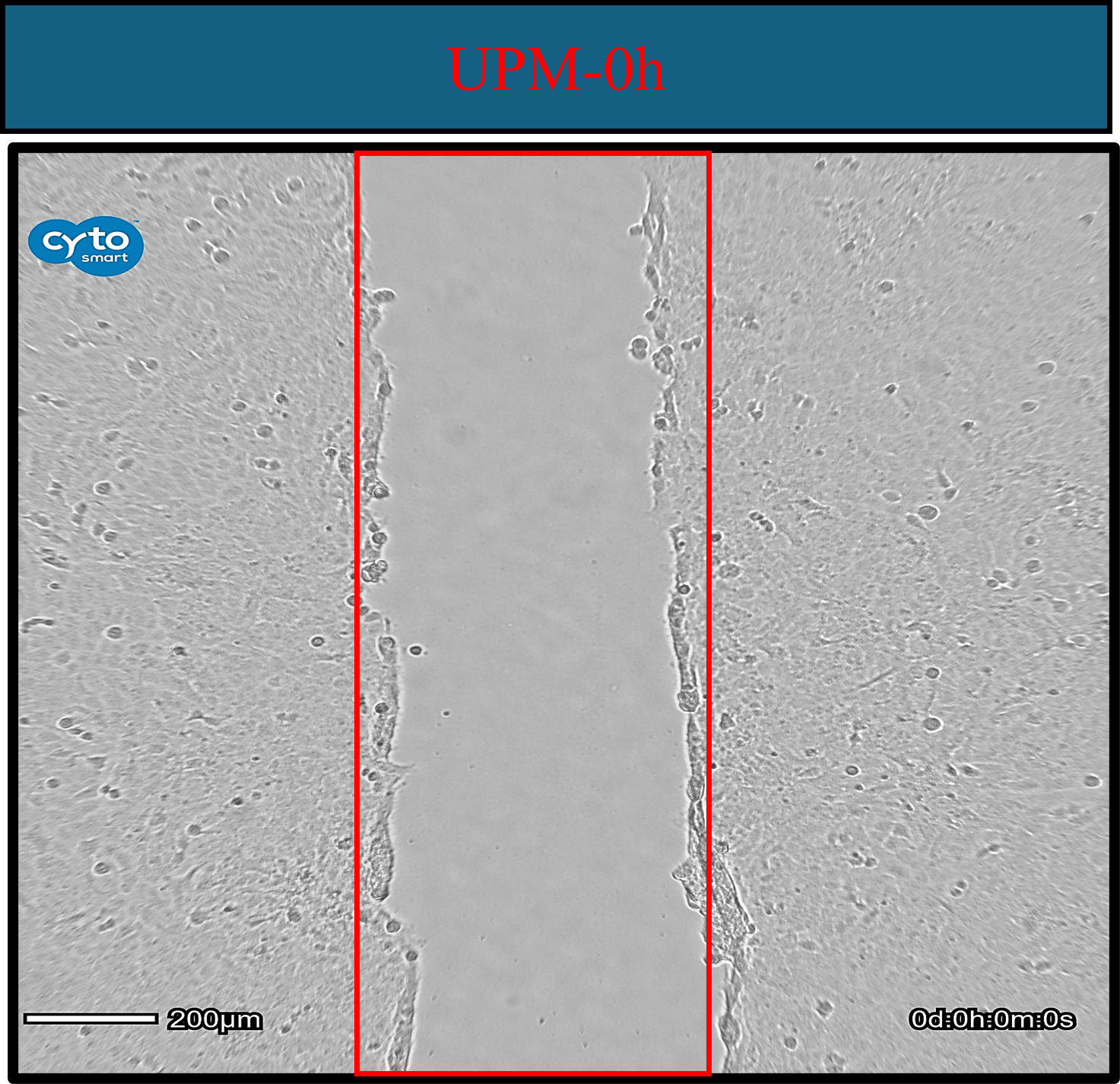

Supplement: Supplementary file 3 — Source data Fig. 1 [file 44321_2026_403_MOESM3_ESM.zip › B/UPM-0h.tif]

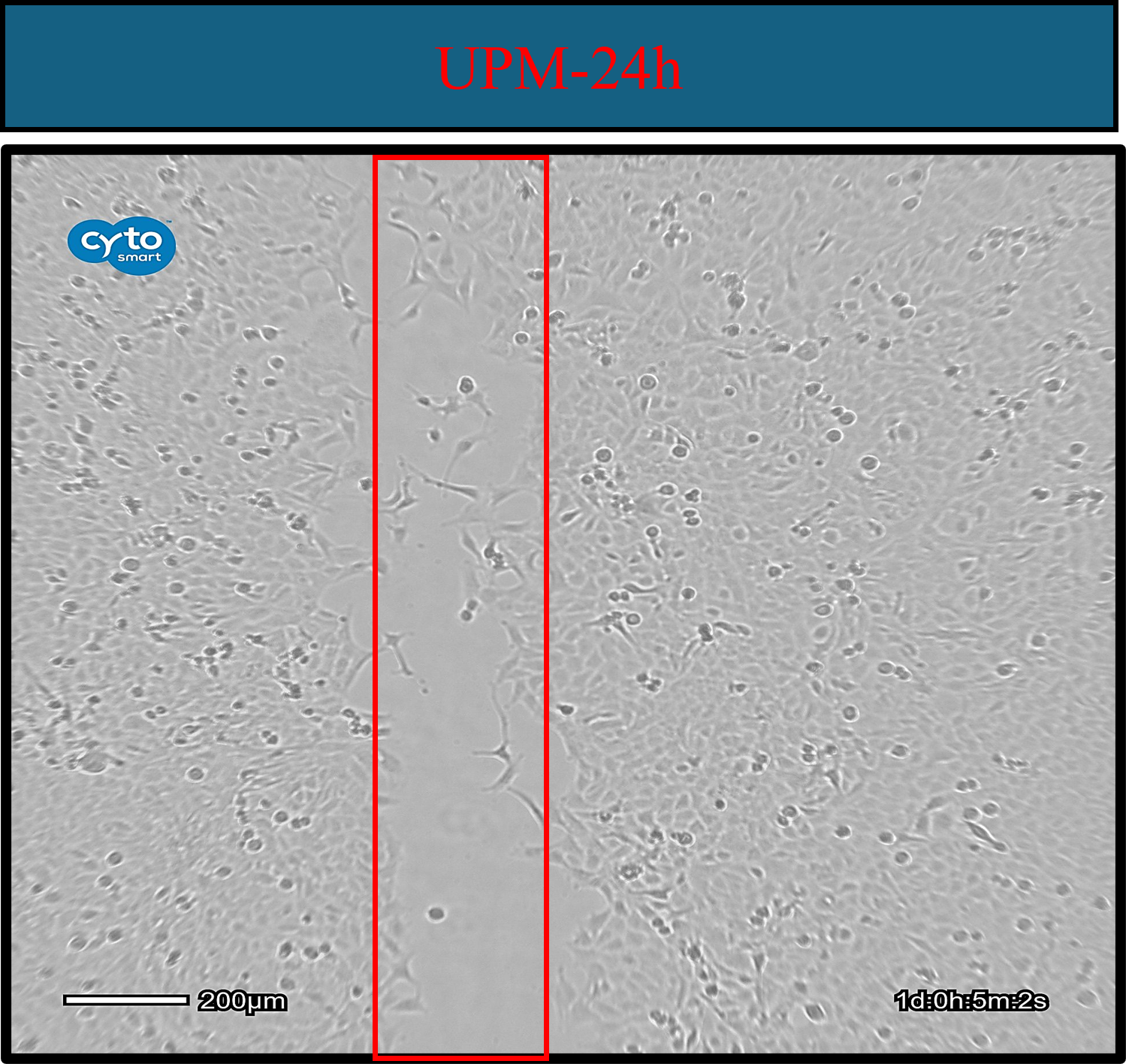

Supplement: Supplementary file 3 — Source data Fig. 1 [file 44321_2026_403_MOESM3_ESM.zip › B/UPM-24h.tif]

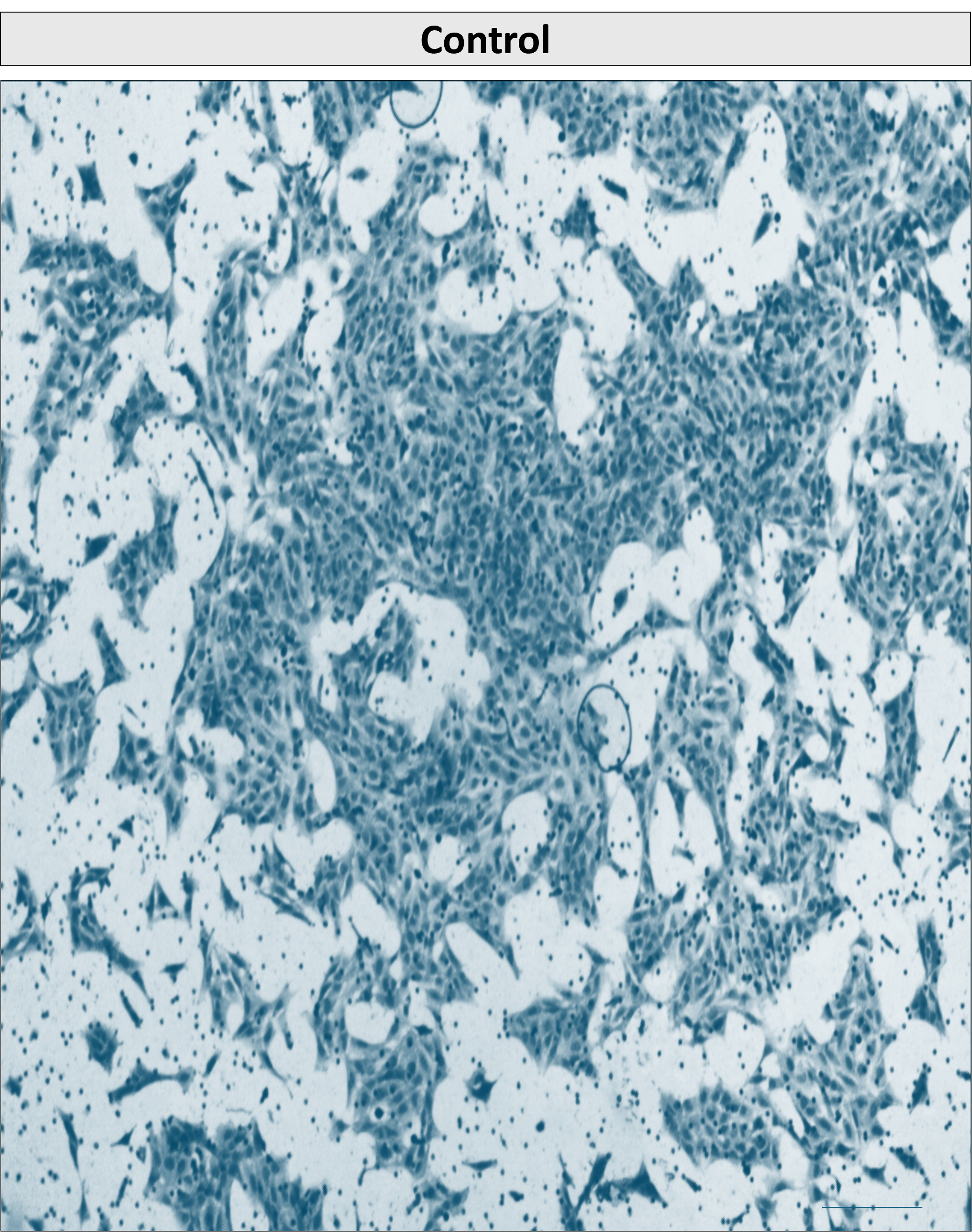

Supplement: Supplementary file 3 — Source data Fig. 1 [file 44321_2026_403_MOESM3_ESM.zip › C/Control.tif]

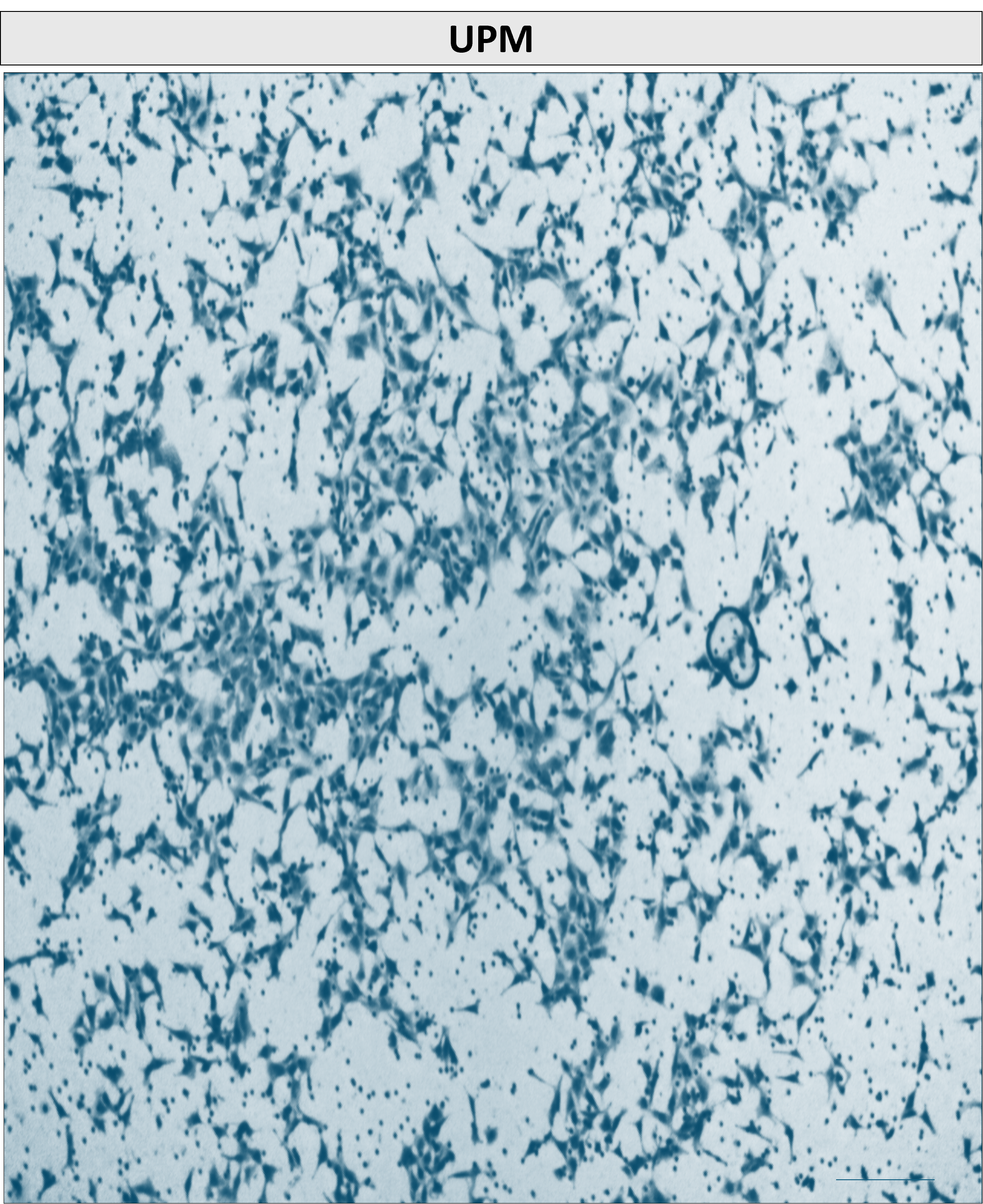

Supplement: Supplementary file 3 — Source data Fig. 1 [file 44321_2026_403_MOESM3_ESM.zip › C/UPM.tif]

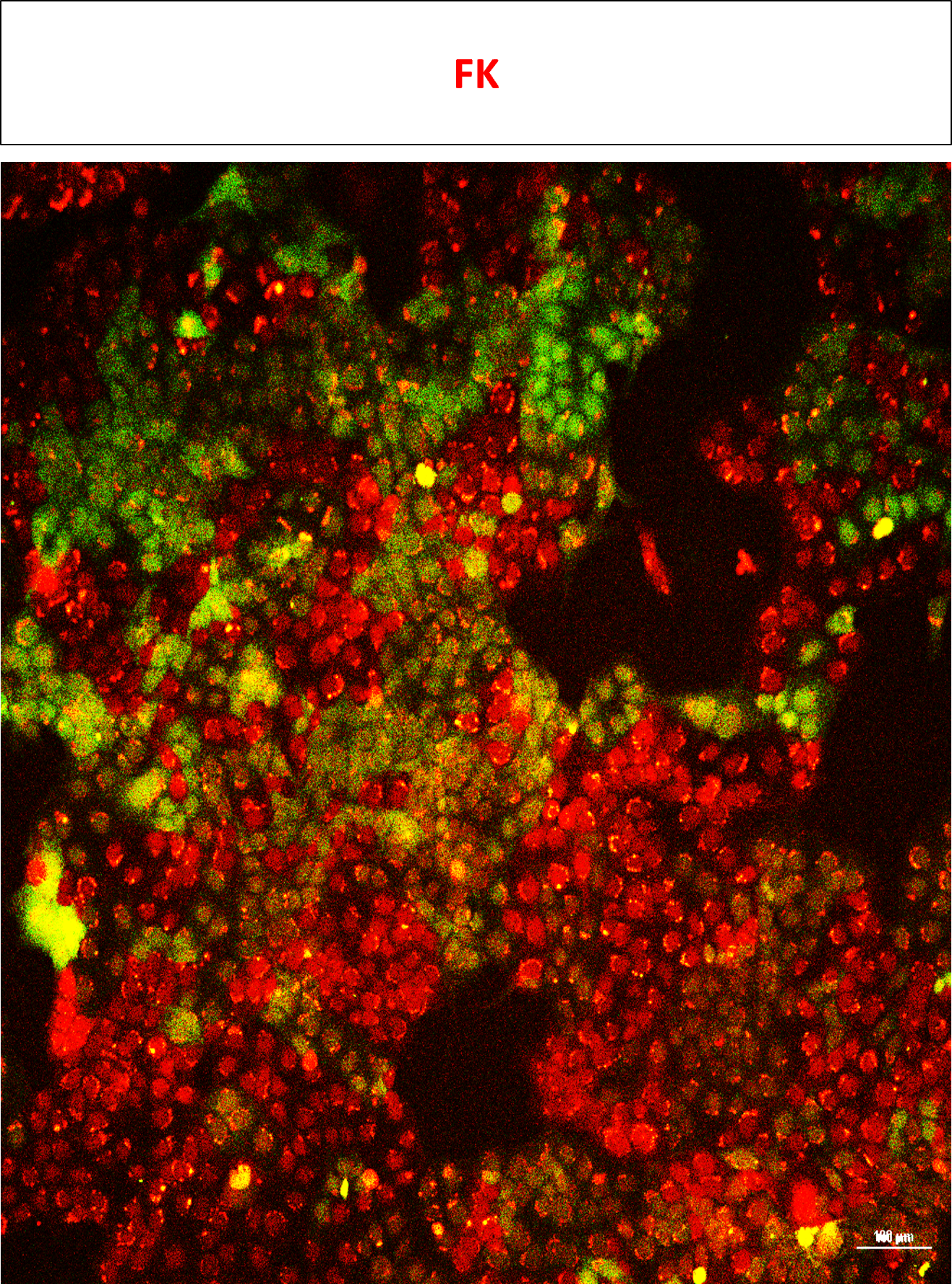

Supplement: Supplementary file 3 — Source data Fig. 1 [file 44321_2026_403_MOESM3_ESM.zip › D/FK.tif]

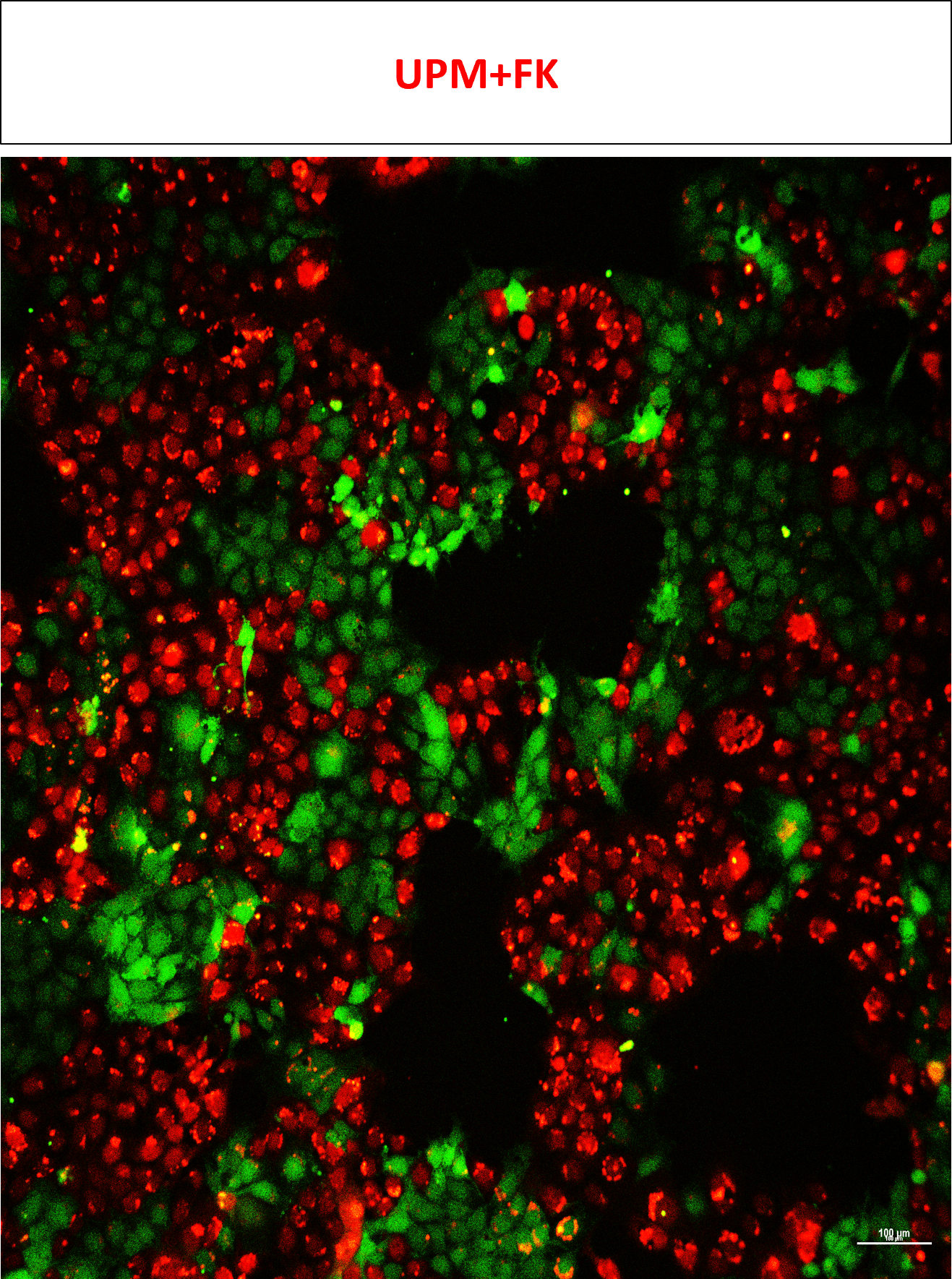

Supplement: Supplementary file 3 — Source data Fig. 1 [file 44321_2026_403_MOESM3_ESM.zip › D/UPM+FK.tif]

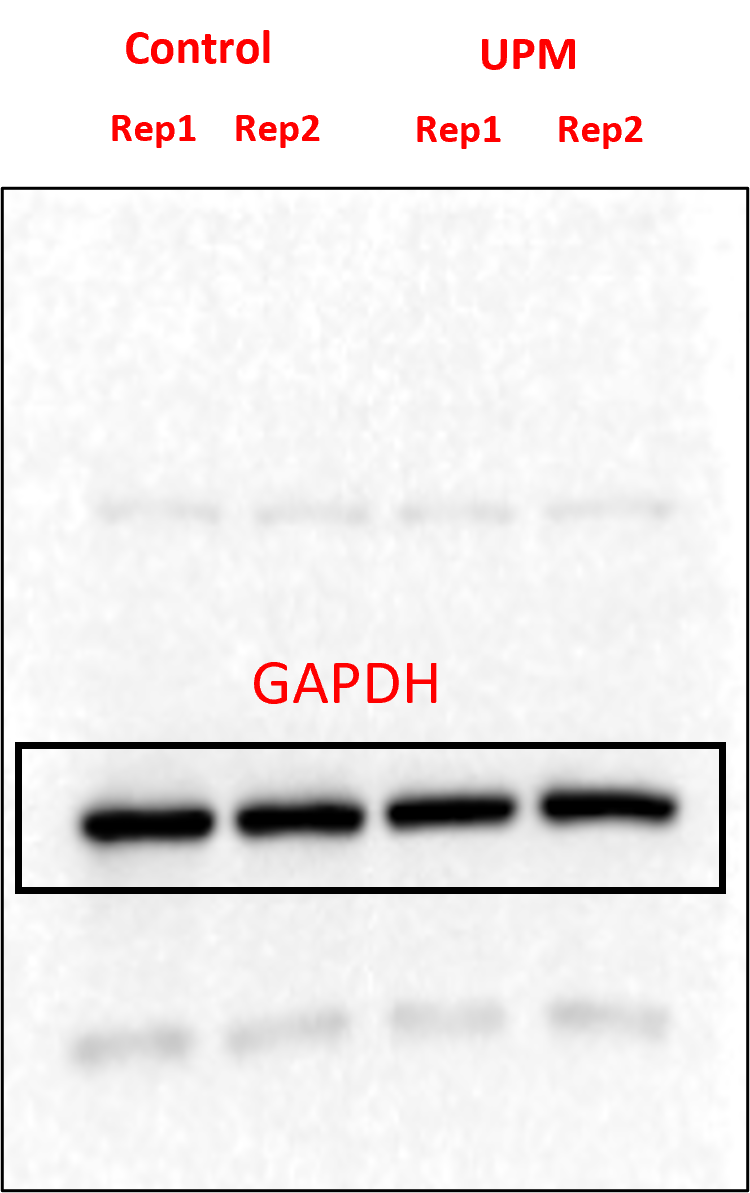

Supplement: Supplementary file 3 — Source data Fig. 1 [file 44321_2026_403_MOESM3_ESM.zip › E/GAPDH.tif]

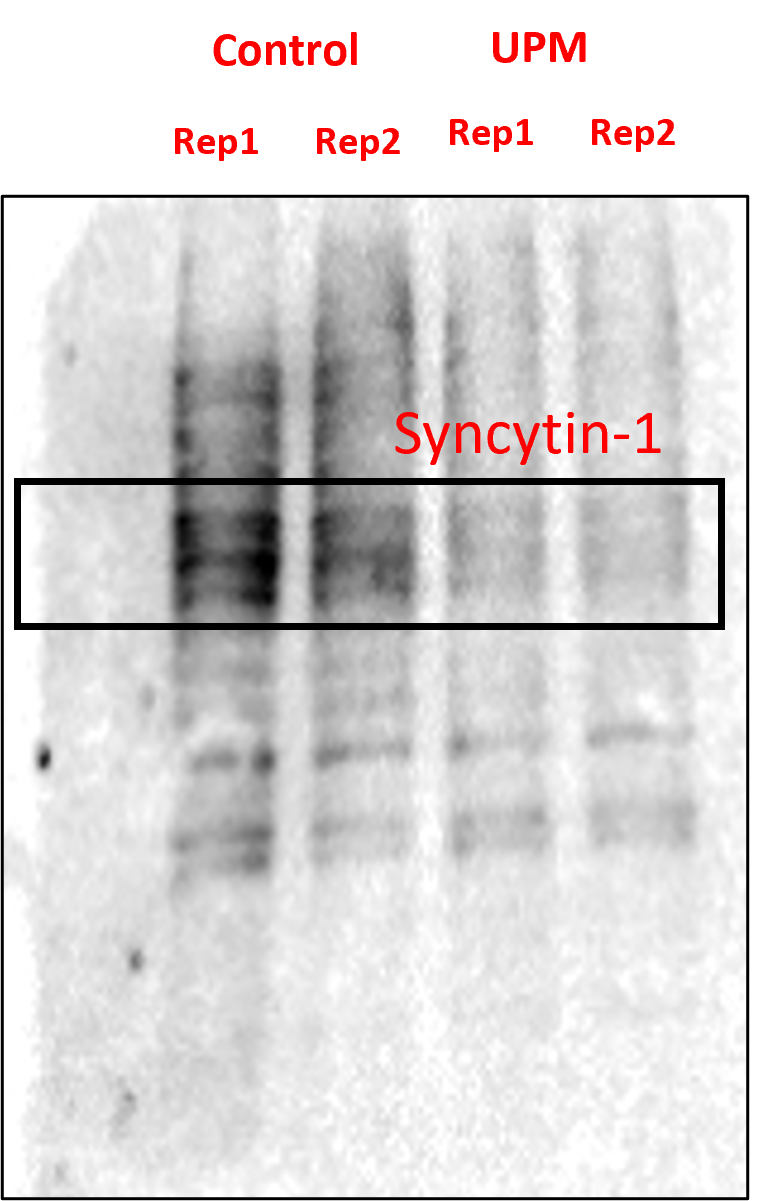

Supplement: Supplementary file 3 — Source data Fig. 1 [file 44321_2026_403_MOESM3_ESM.zip › E/Syncytin-1.tif]

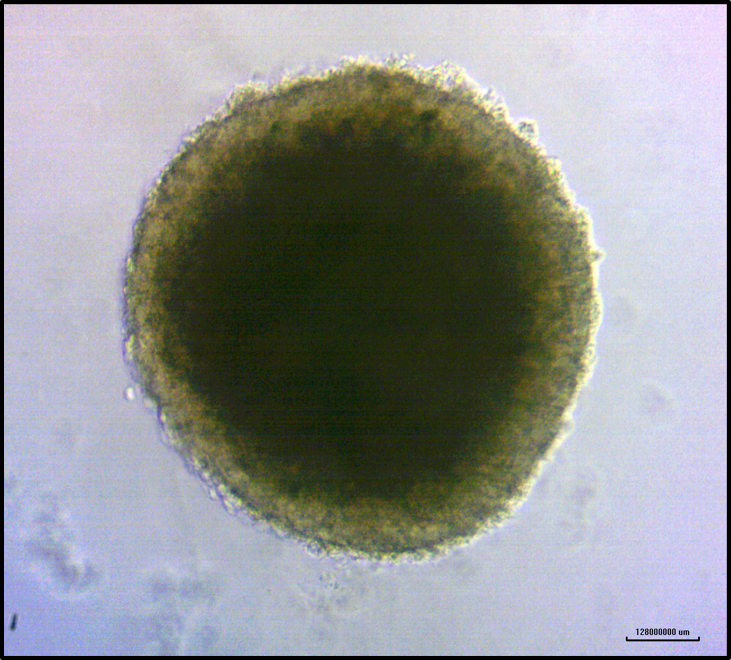

Supplement: Supplementary file 3 — Source data Fig. 1 [file 44321_2026_403_MOESM3_ESM.zip › F/Control.tif]

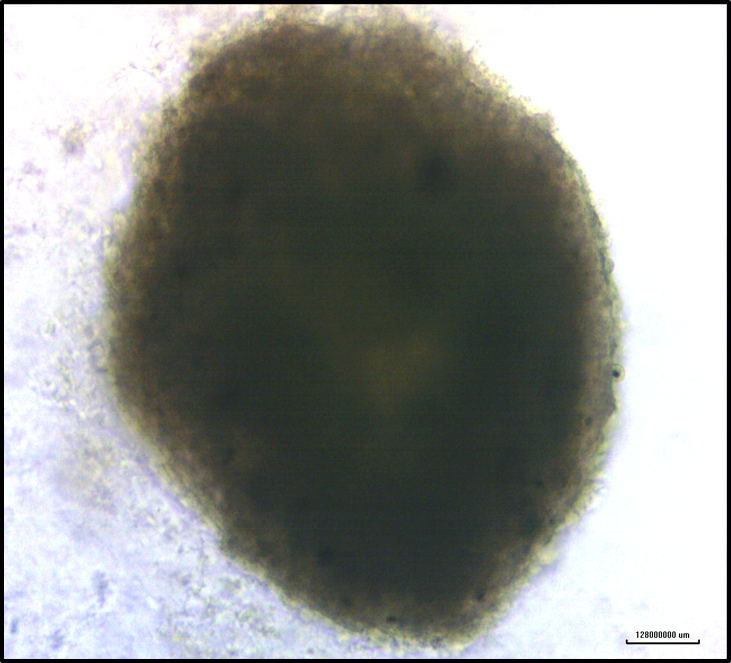

Supplement: Supplementary file 3 — Source data Fig. 1 [file 44321_2026_403_MOESM3_ESM.zip › F/UPM.tif]

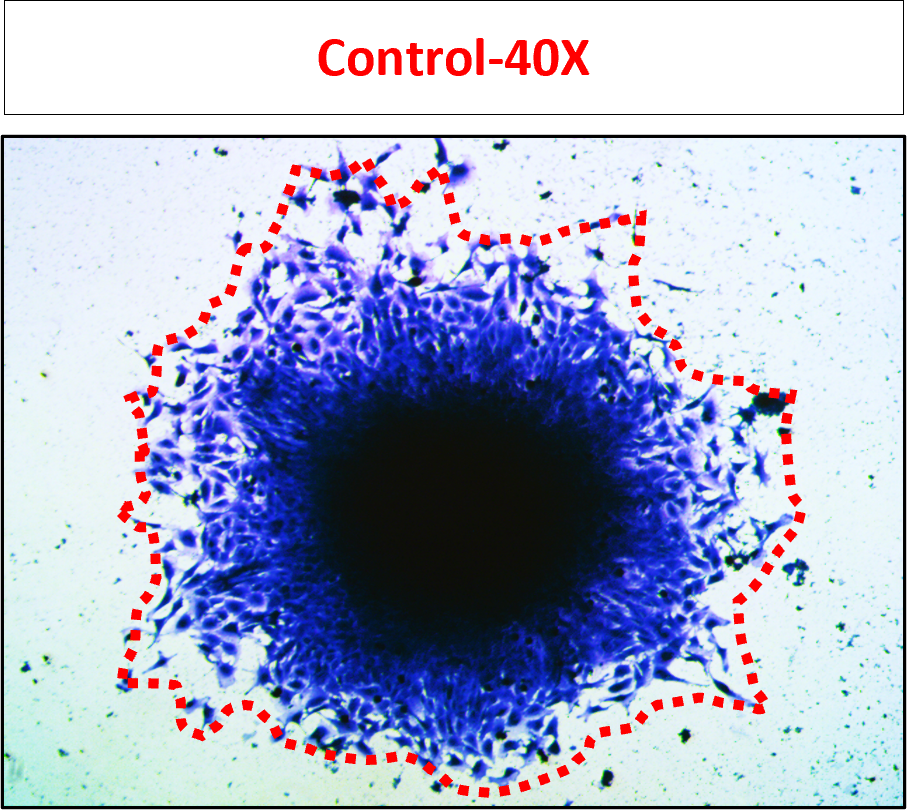

Supplement: Supplementary file 3 — Source data Fig. 1 [file 44321_2026_403_MOESM3_ESM.zip › G/Control-1.tif]

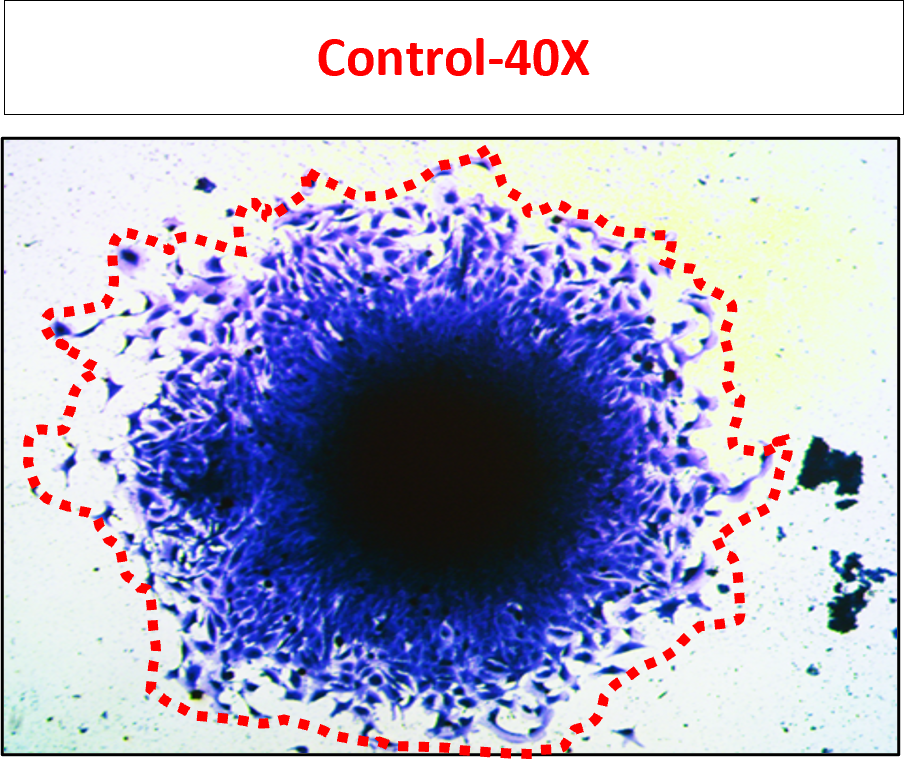

Supplement: Supplementary file 3 — Source data Fig. 1 [file 44321_2026_403_MOESM3_ESM.zip › G/Control-2.tif]

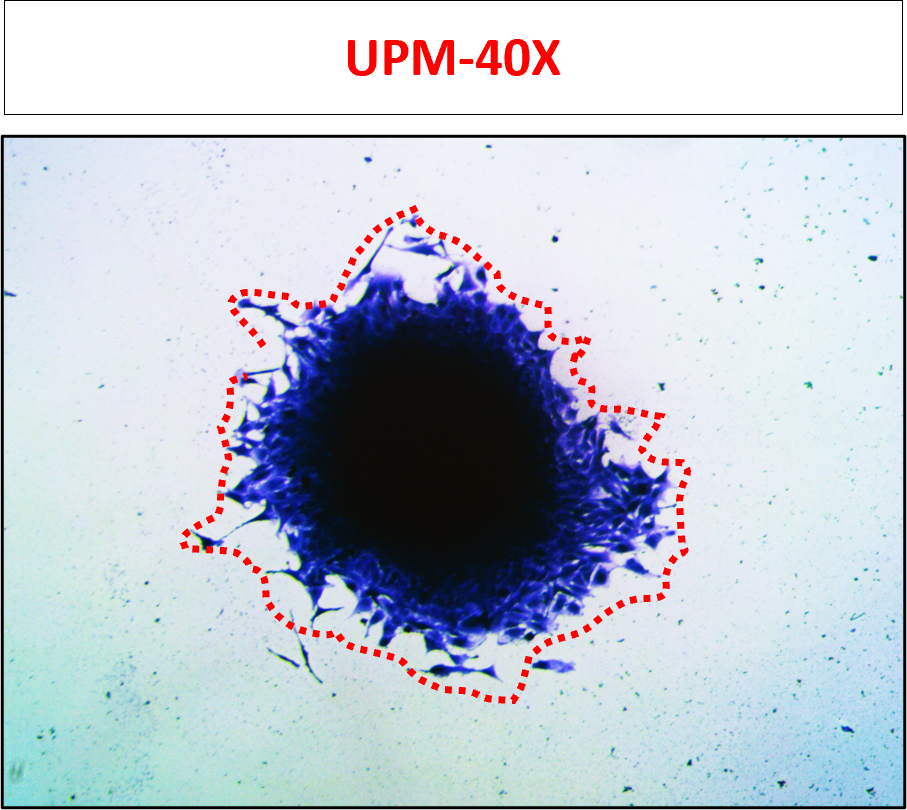

Supplement: Supplementary file 3 — Source data Fig. 1 [file 44321_2026_403_MOESM3_ESM.zip › G/UPM-1.tif]

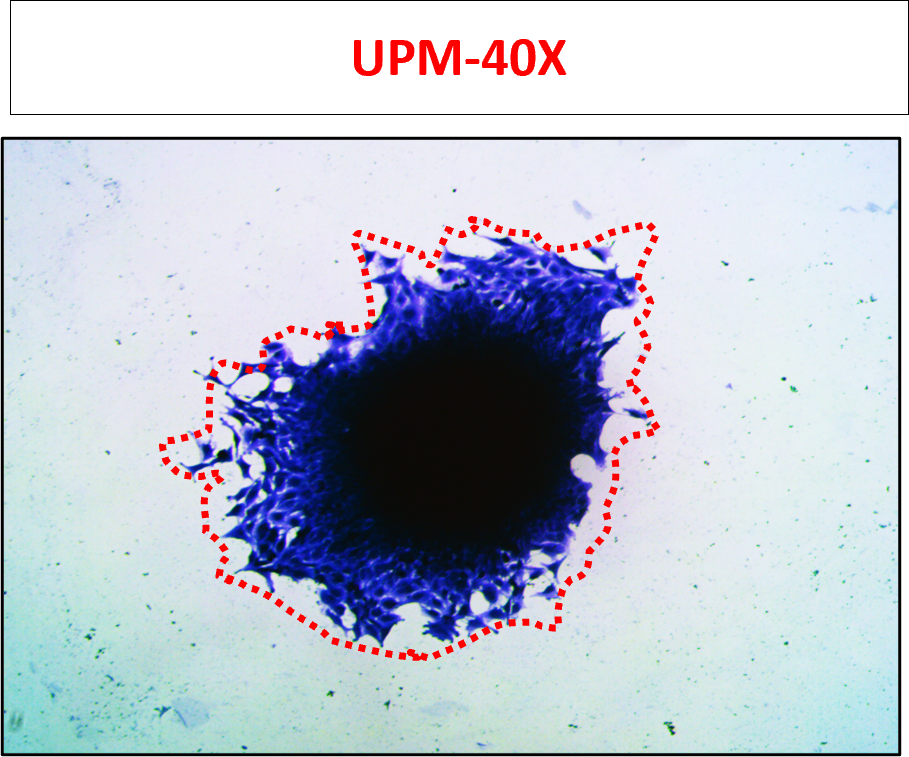

Supplement: Supplementary file 3 — Source data Fig. 1 [file 44321_2026_403_MOESM3_ESM.zip › G/UPM-2.tif]

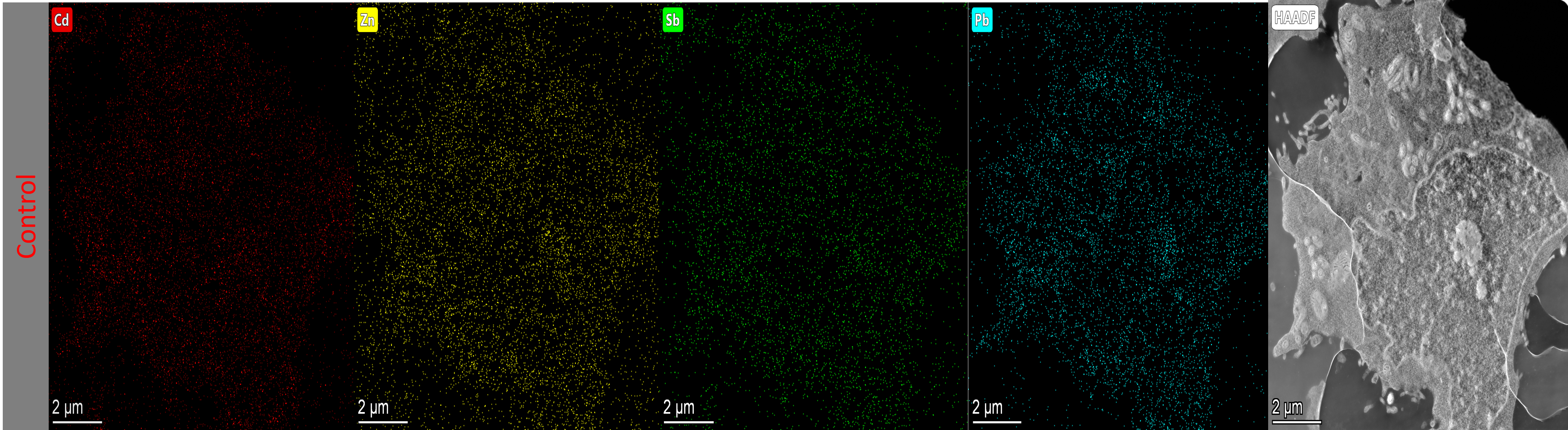

Supplement: Supplementary file 3 — Source data Fig. 1 [file 44321_2026_403_MOESM3_ESM.zip › J/Control.tif]

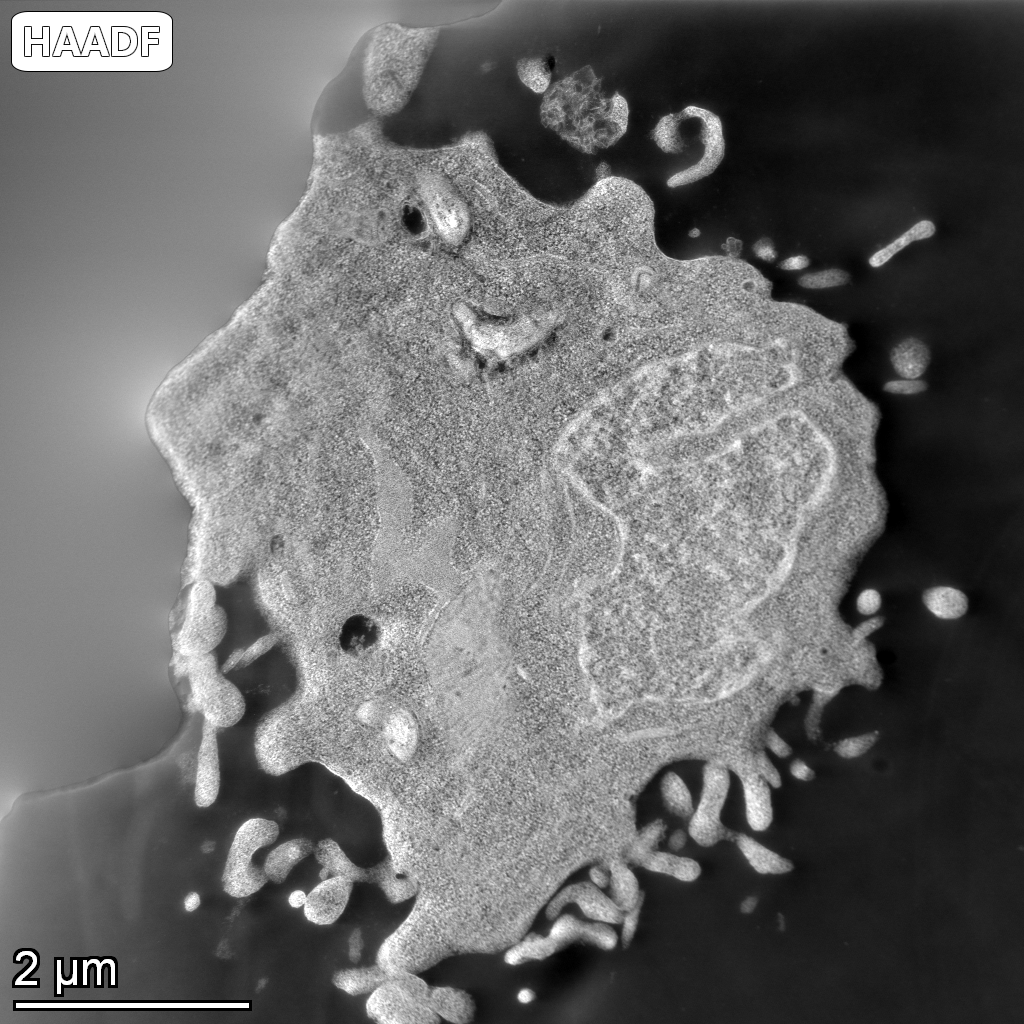

Supplement: Supplementary file 3 — Source data Fig. 1 [file 44321_2026_403_MOESM3_ESM.zip › J/SI 1121 11500 x-HAADF.tif]

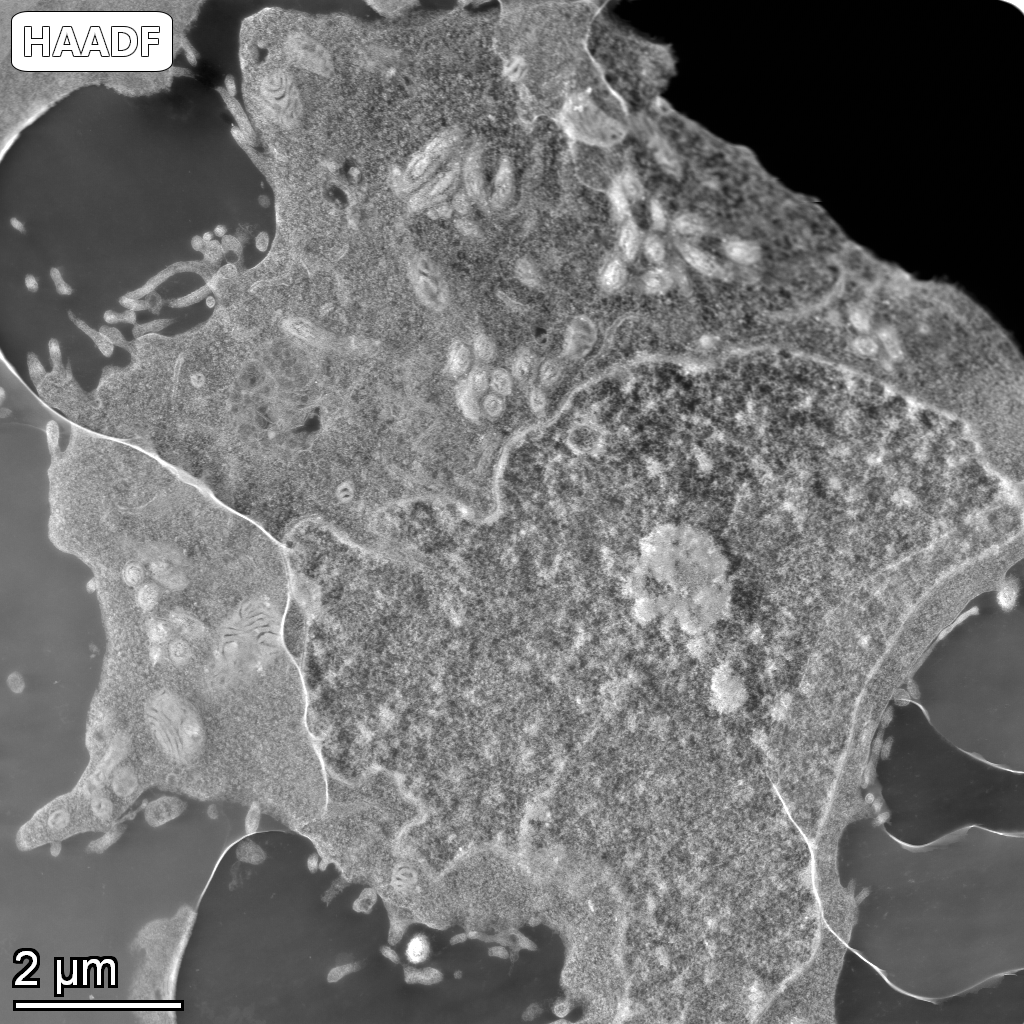

Supplement: Supplementary file 3 — Source data Fig. 1 [file 44321_2026_403_MOESM3_ESM.zip › J/SI 1205 8000 x-HAADF.tif]

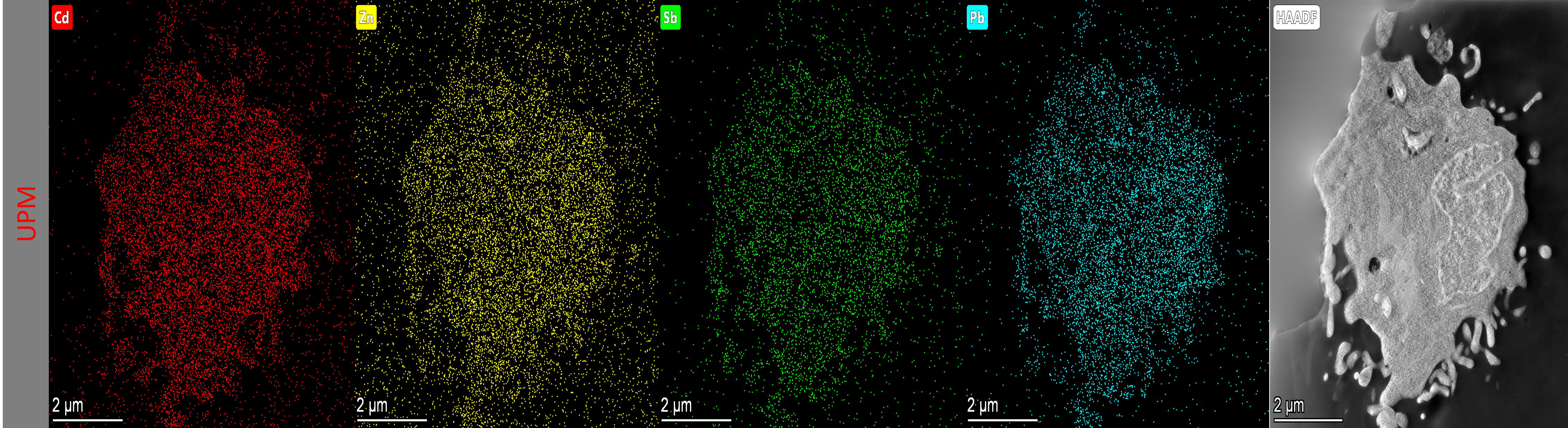

Supplement: Supplementary file 3 — Source data Fig. 1 [file 44321_2026_403_MOESM3_ESM.zip › J/UPM.tif]

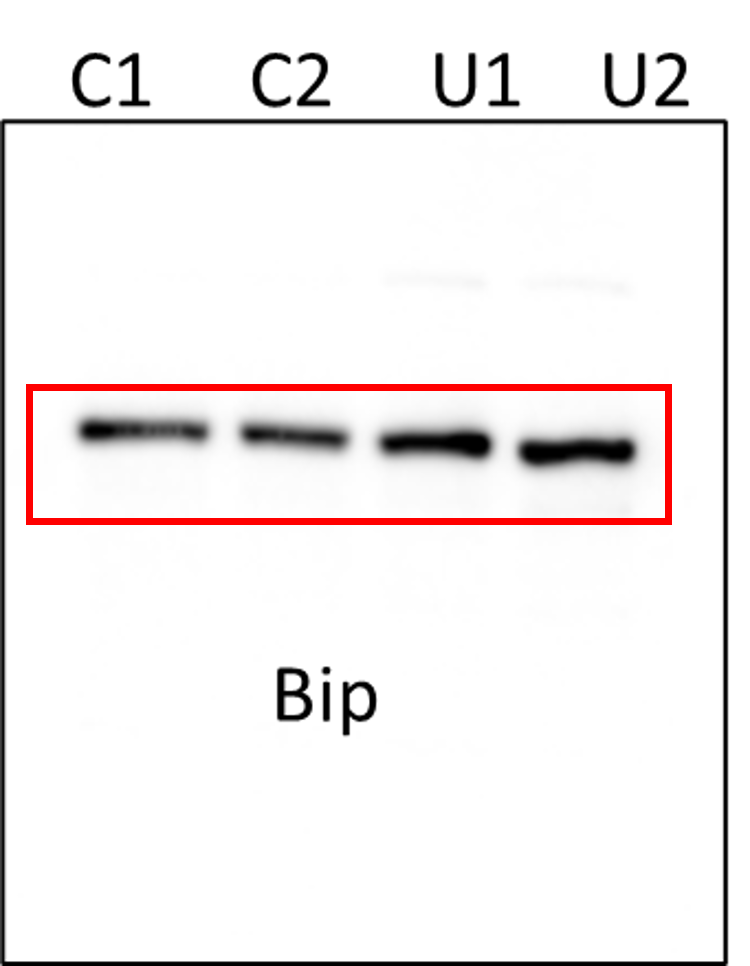

Supplement: Supplementary file 4 — Source data Fig. 3 [file 44321_2026_403_MOESM4_ESM.zip › A/BiP.tif]

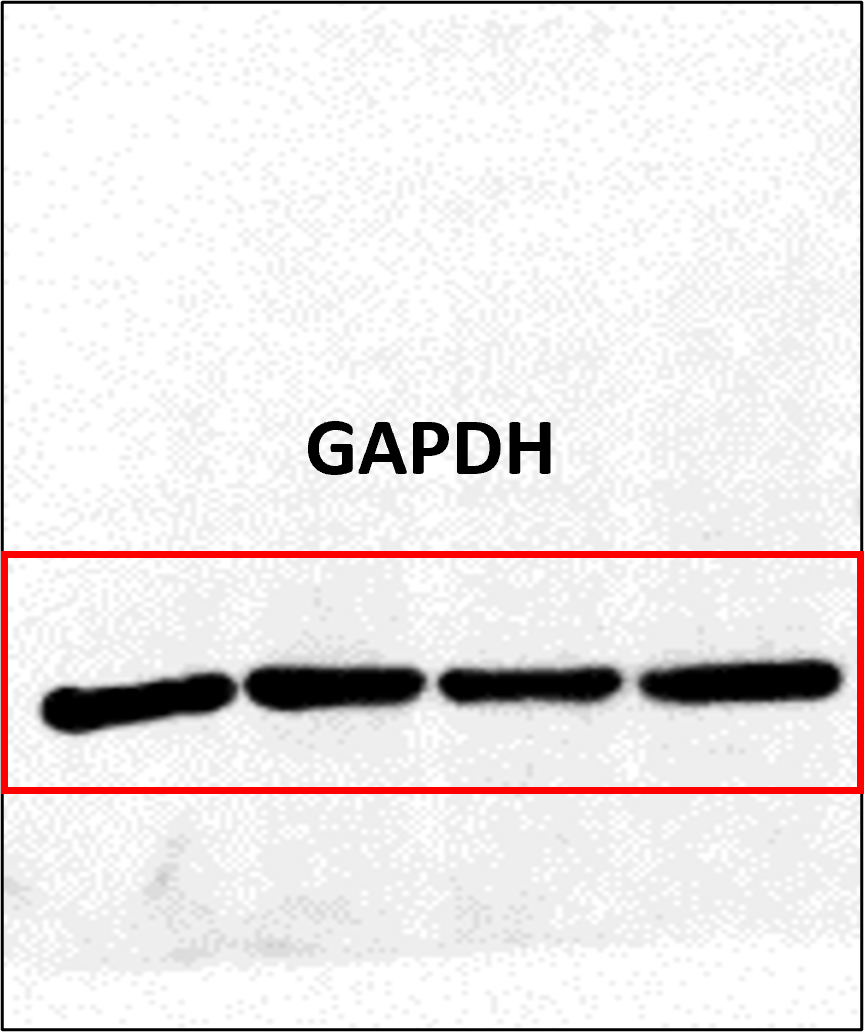

Supplement: Supplementary file 4 — Source data Fig. 3 [file 44321_2026_403_MOESM4_ESM.zip › A/GAPDH.tif]

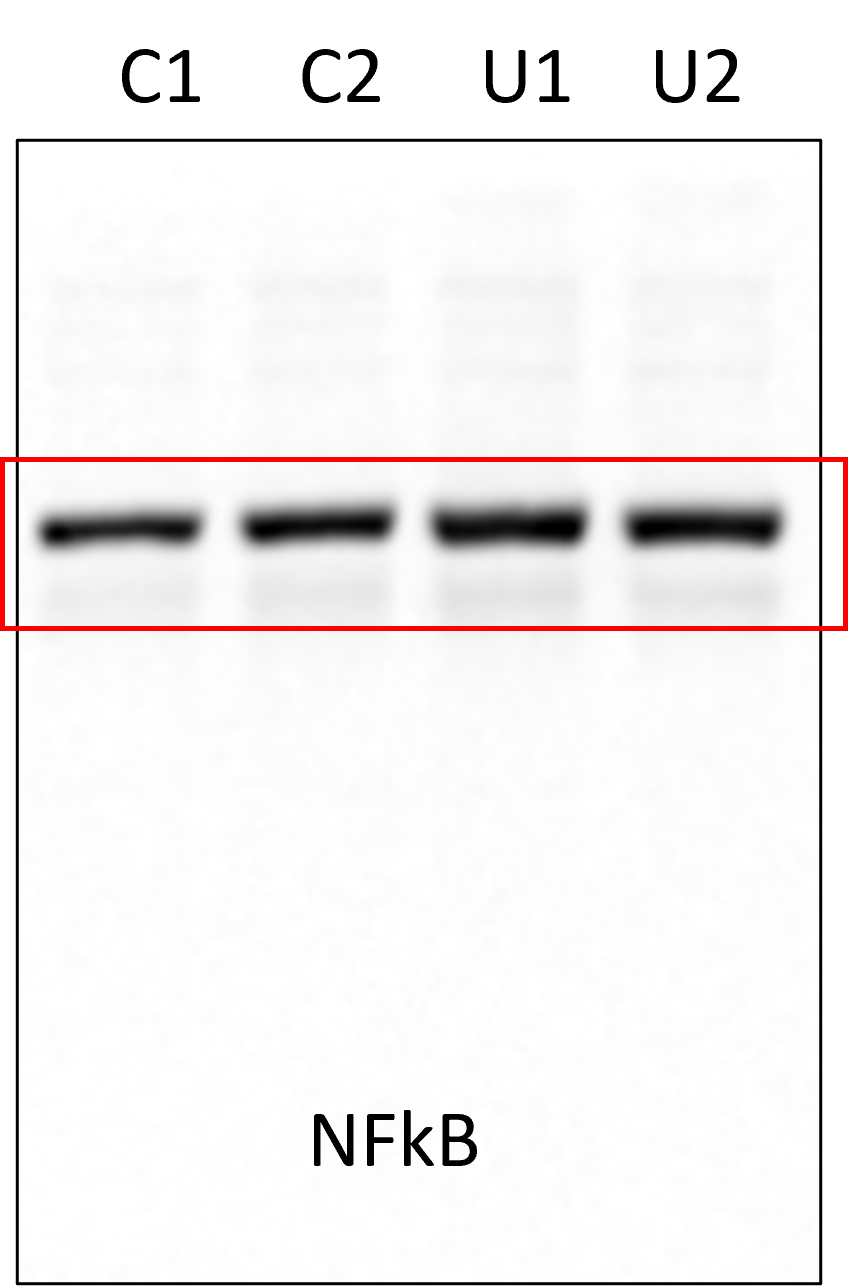

Supplement: Supplementary file 4 — Source data Fig. 3 [file 44321_2026_403_MOESM4_ESM.zip › A/NFKB.tif]

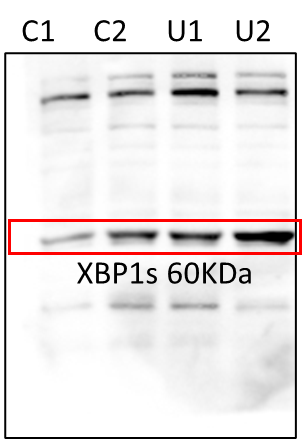

Supplement: Supplementary file 4 — Source data Fig. 3 [file 44321_2026_403_MOESM4_ESM.zip › A/XBP1s.tif]

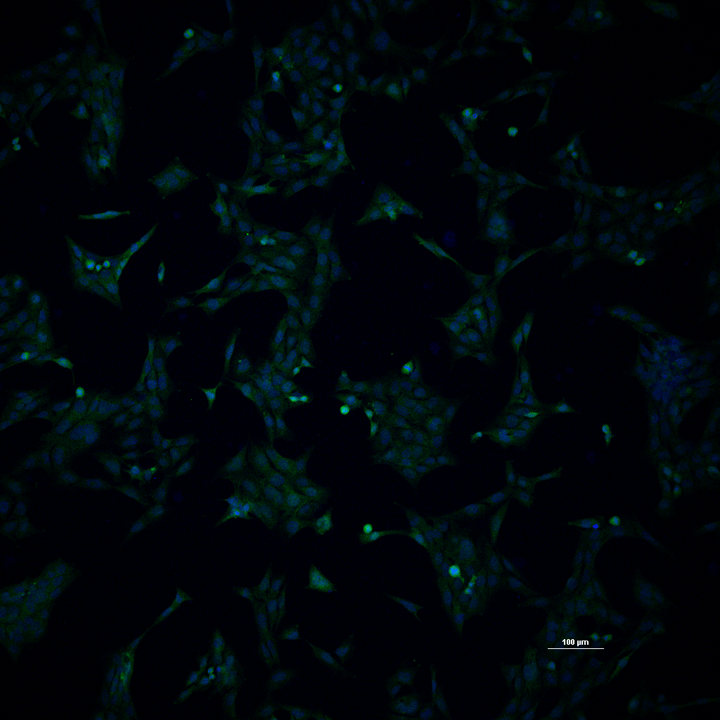

Supplement: Supplementary file 4 — Source data Fig. 3 [file 44321_2026_403_MOESM4_ESM.zip › B/Control 4X merged.tif]

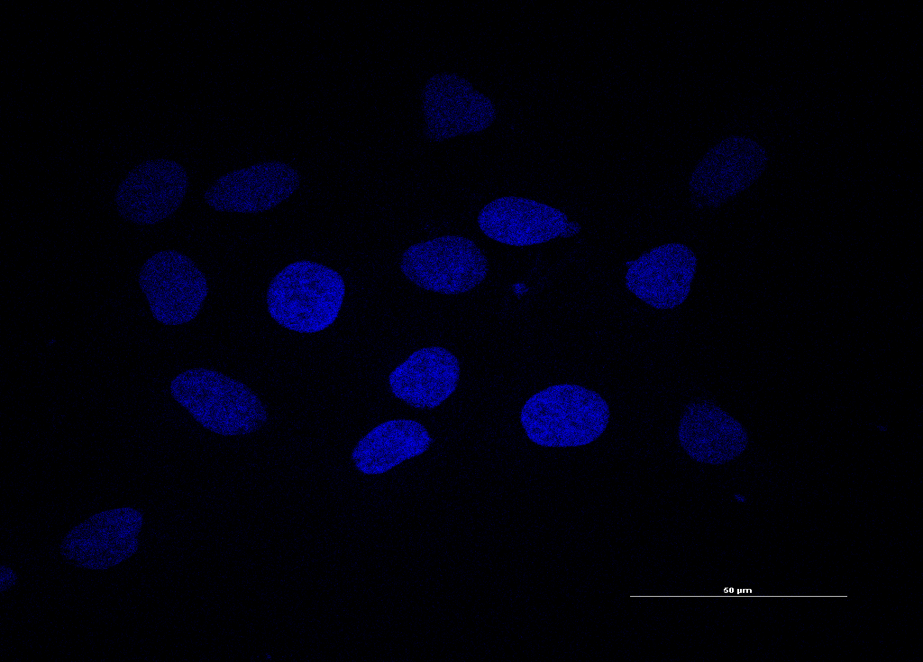

Supplement: Supplementary file 4 — Source data Fig. 3 [file 44321_2026_403_MOESM4_ESM.zip › B/Control-60X-DAPI.tif]

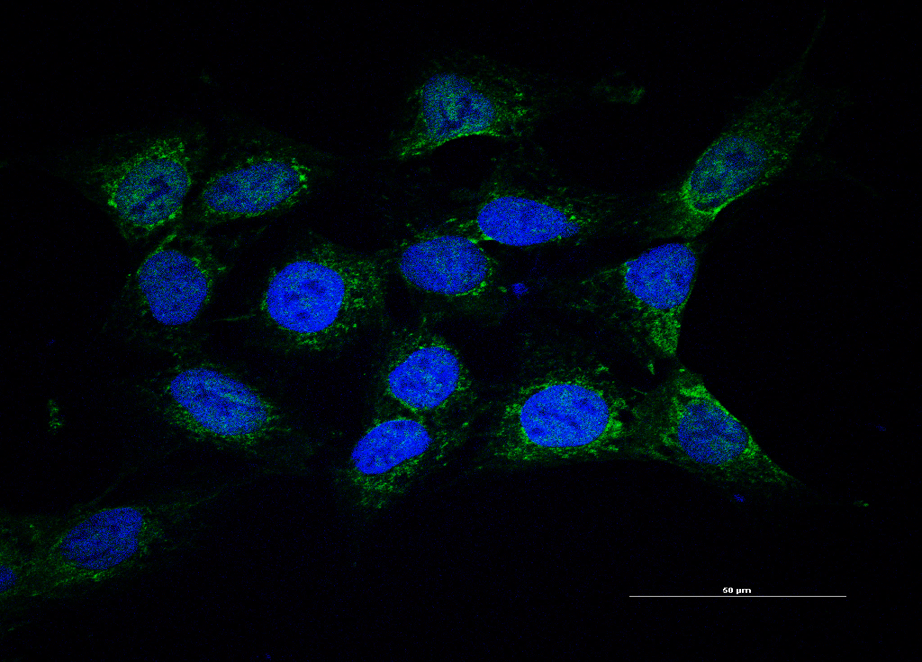

Supplement: Supplementary file 4 — Source data Fig. 3 [file 44321_2026_403_MOESM4_ESM.zip › B/Control-60X-Merged.tif]

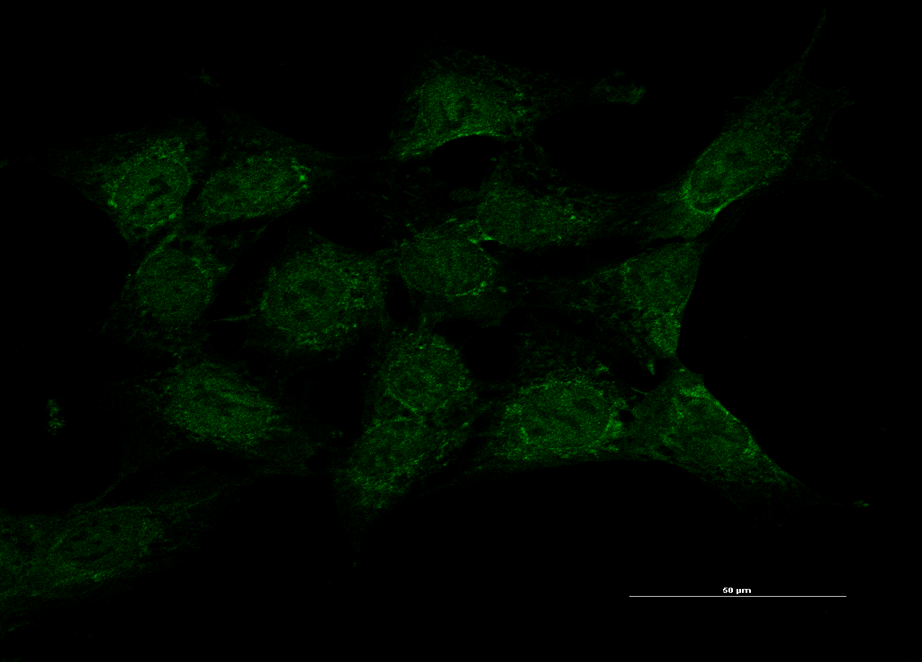

Supplement: Supplementary file 4 — Source data Fig. 3 [file 44321_2026_403_MOESM4_ESM.zip › B/Control-60X-XBP1s.tif]

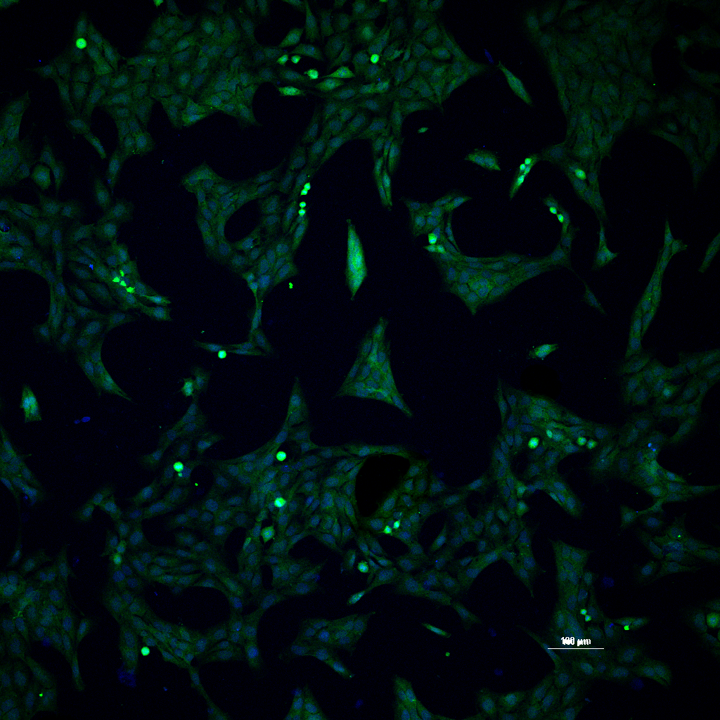

Supplement: Supplementary file 4 — Source data Fig. 3 [file 44321_2026_403_MOESM4_ESM.zip › B/UPM 4X merged.tif]

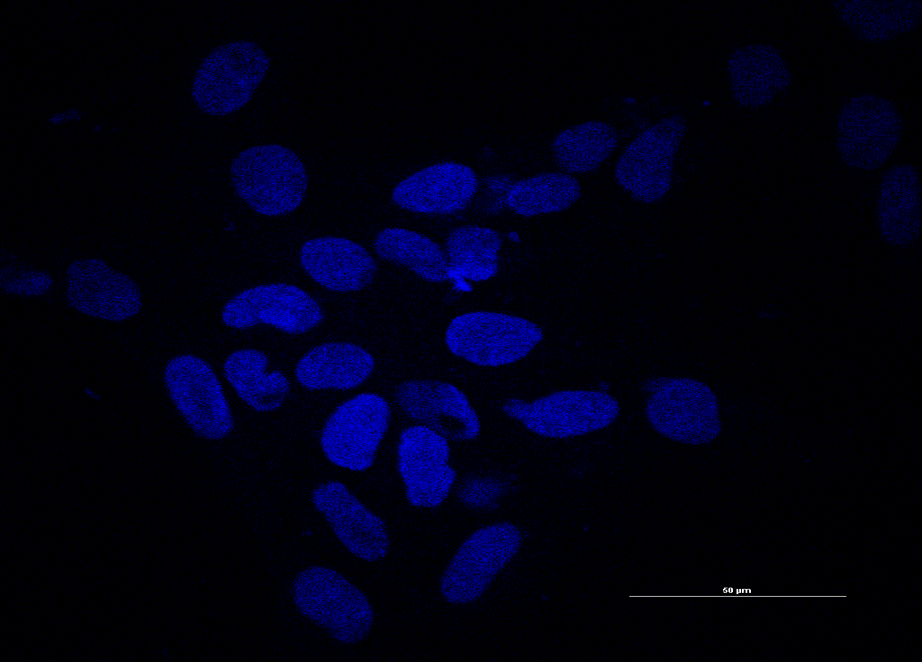

Supplement: Supplementary file 4 — Source data Fig. 3 [file 44321_2026_403_MOESM4_ESM.zip › B/UPM-60X-DAPI.tif]

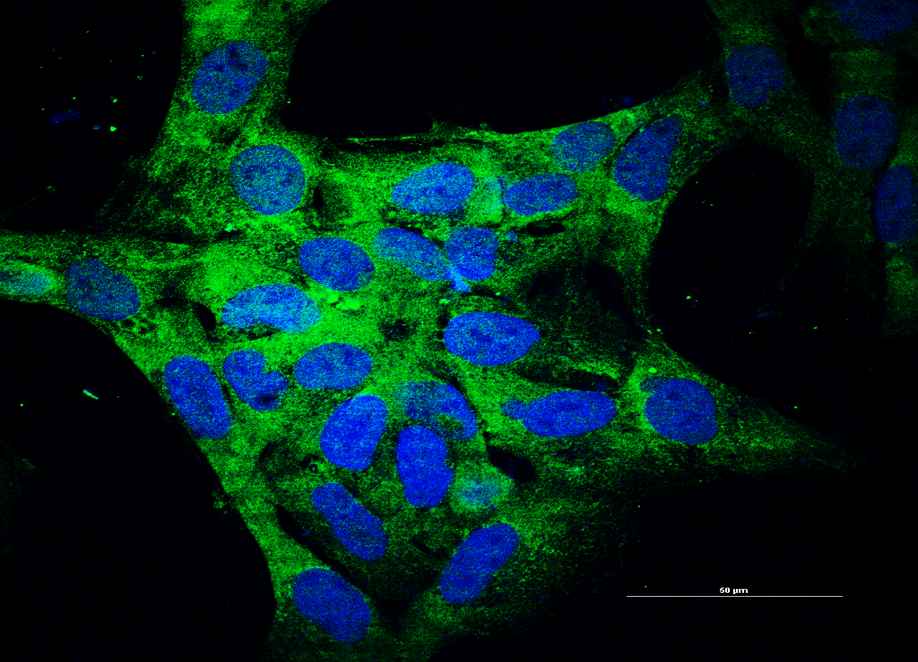

Supplement: Supplementary file 4 — Source data Fig. 3 [file 44321_2026_403_MOESM4_ESM.zip › B/UPM-60X-Merged.tif]

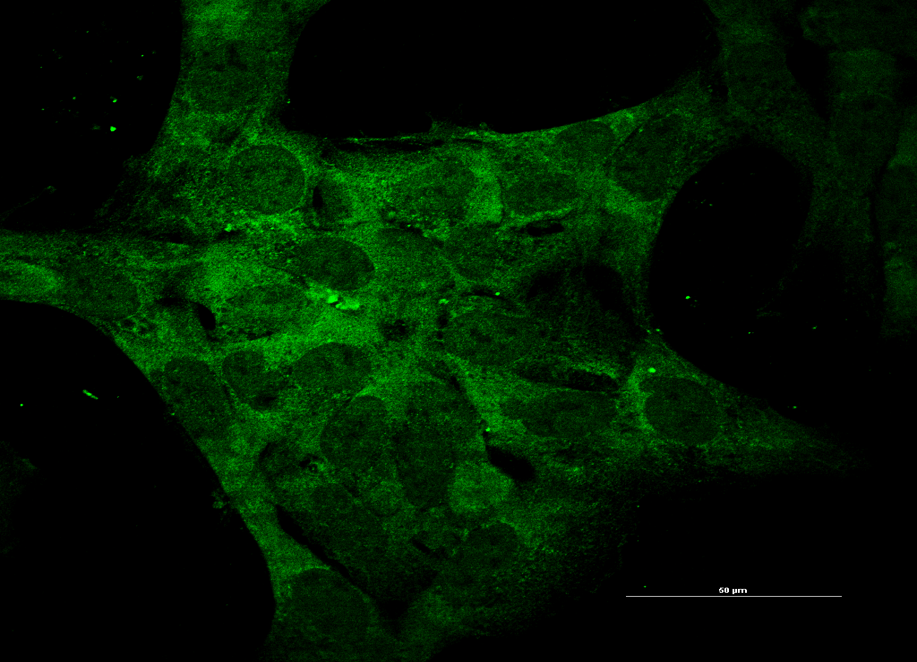

Supplement: Supplementary file 4 — Source data Fig. 3 [file 44321_2026_403_MOESM4_ESM.zip › B/UPM-60X-XBP1s.tif]

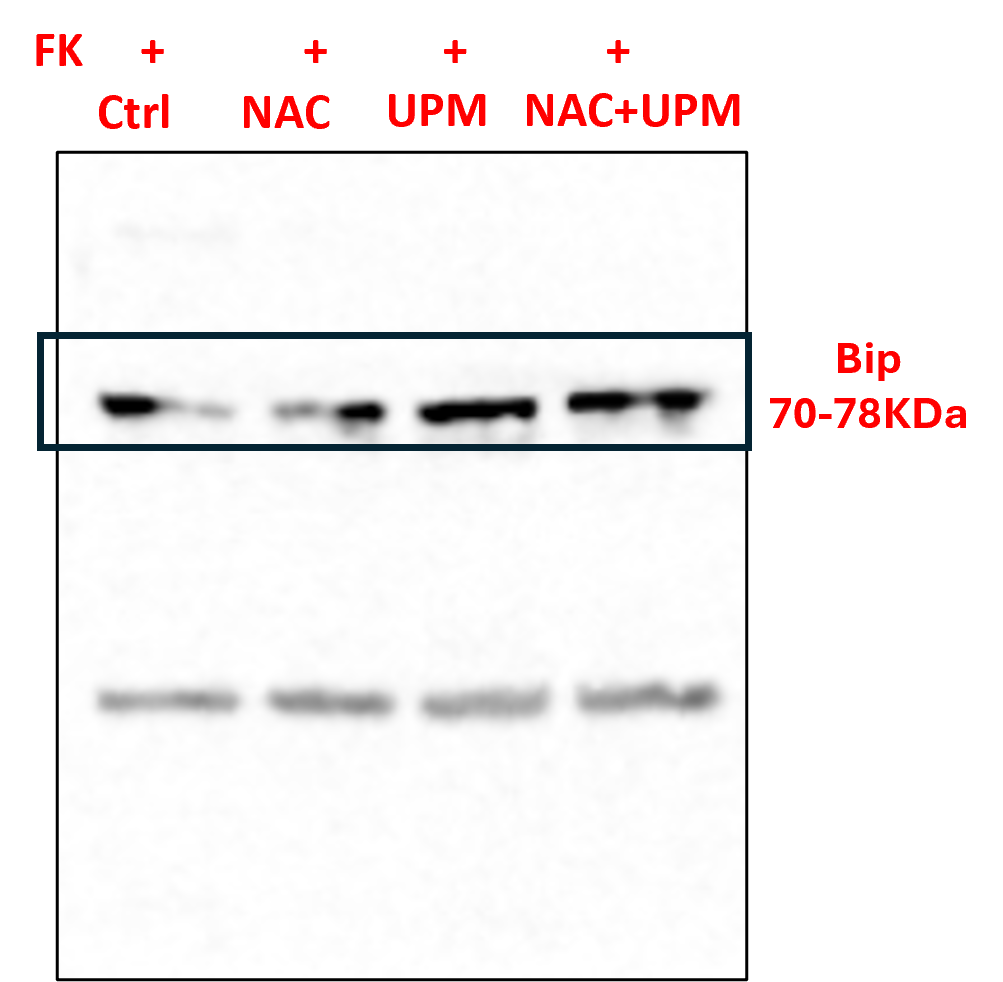

Supplement: Supplementary file 4 — Source data Fig. 3 [file 44321_2026_403_MOESM4_ESM.zip › C/Bip(NAC).tif]

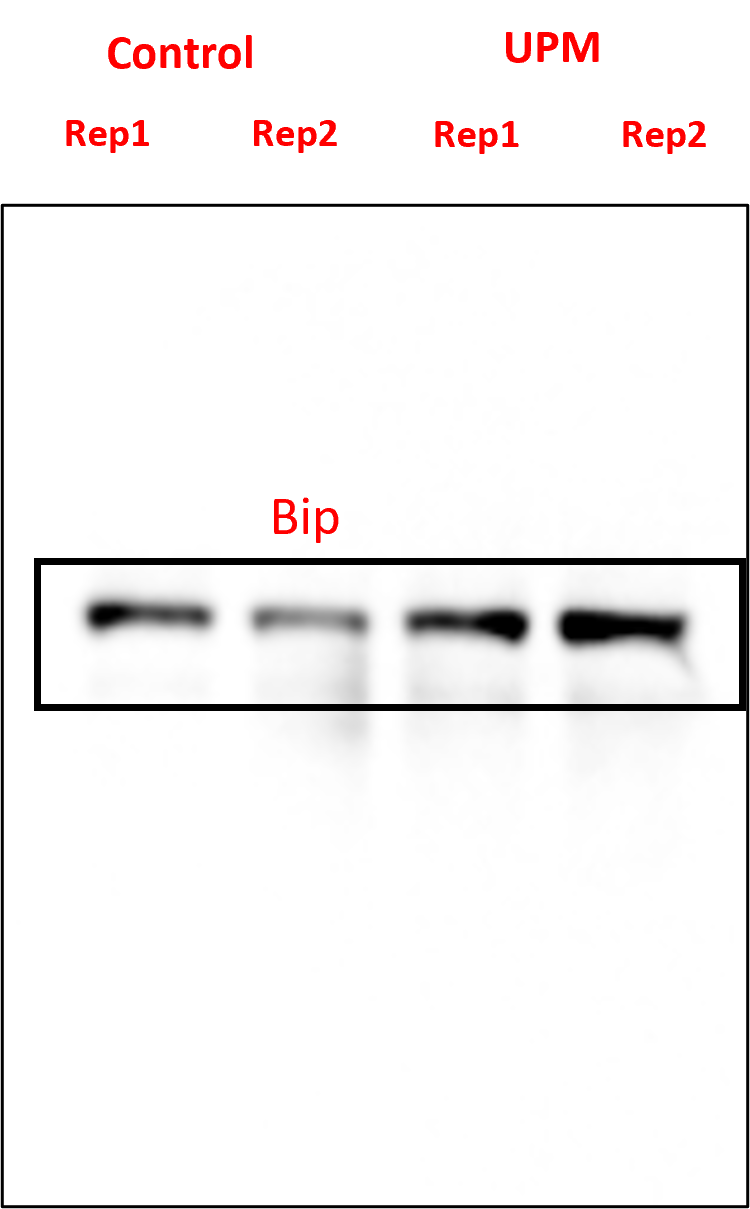

Supplement: Supplementary file 4 — Source data Fig. 3 [file 44321_2026_403_MOESM4_ESM.zip › C/Bip.tif]

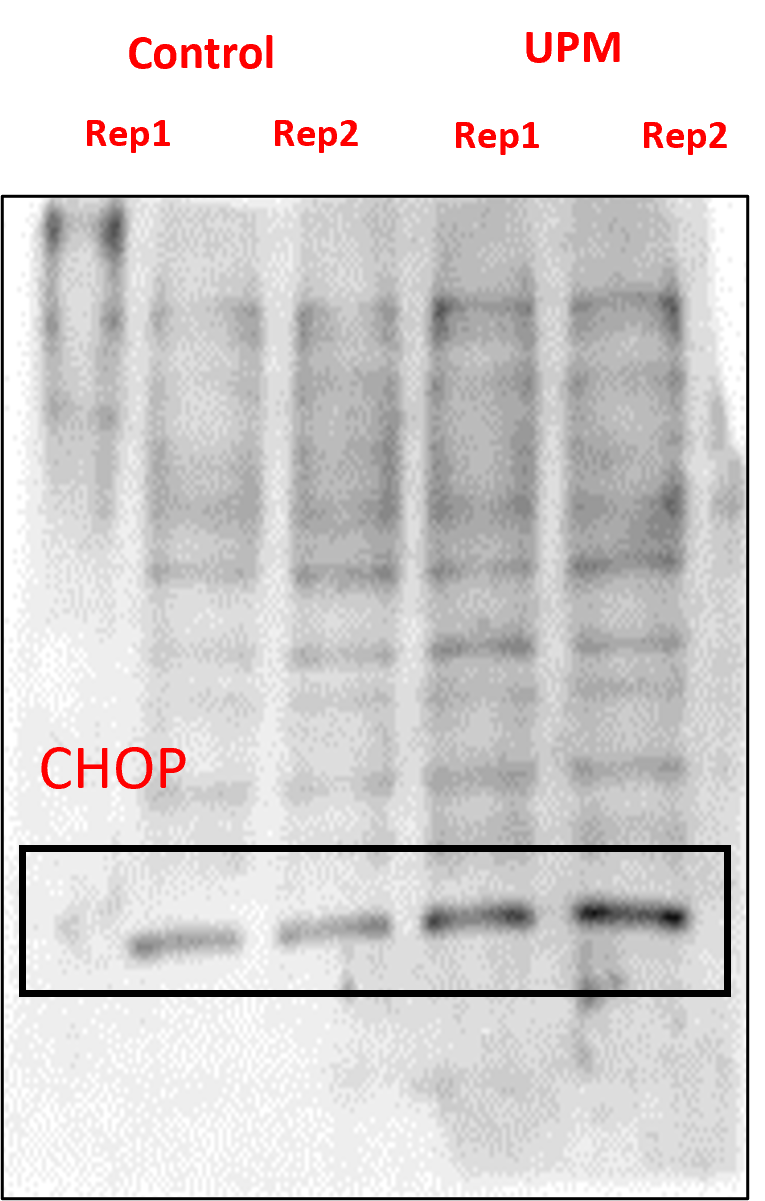

Supplement: Supplementary file 4 — Source data Fig. 3 [file 44321_2026_403_MOESM4_ESM.zip › C/CHOP.tif]

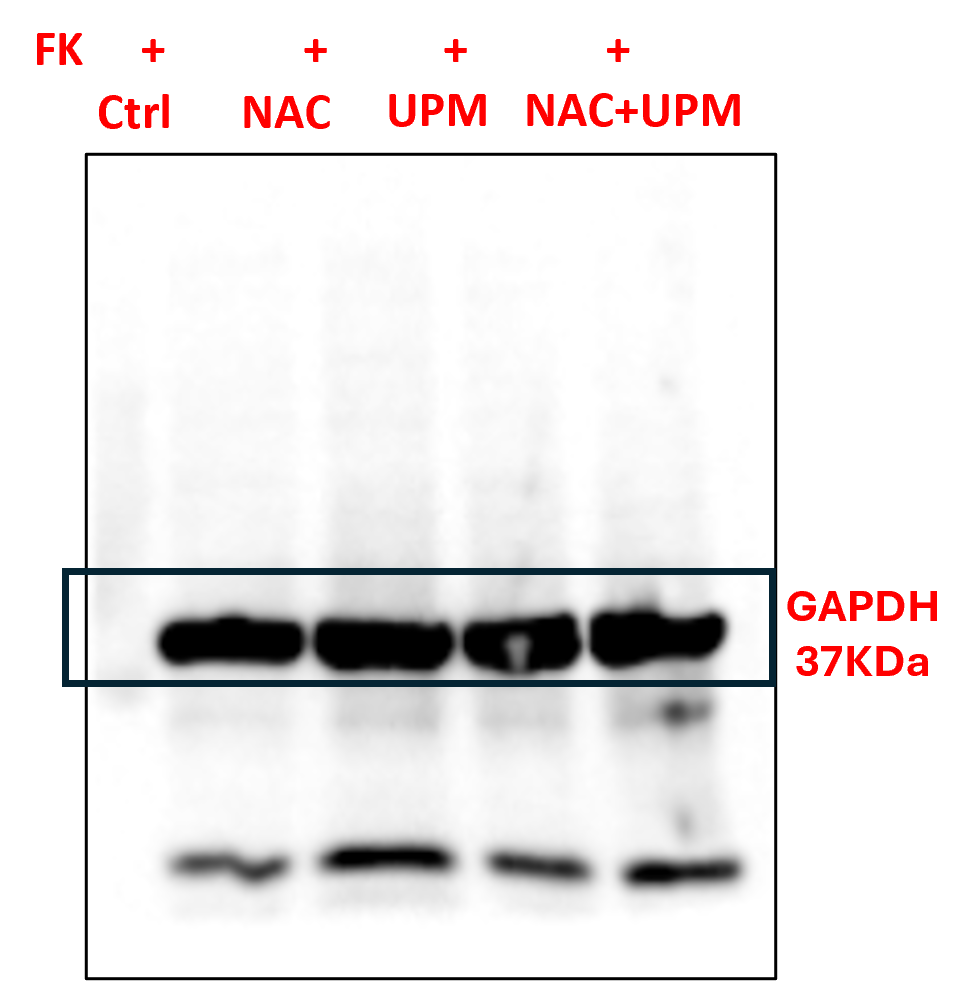

Supplement: Supplementary file 4 — Source data Fig. 3 [file 44321_2026_403_MOESM4_ESM.zip › C/GAPDH (NAC).tif]

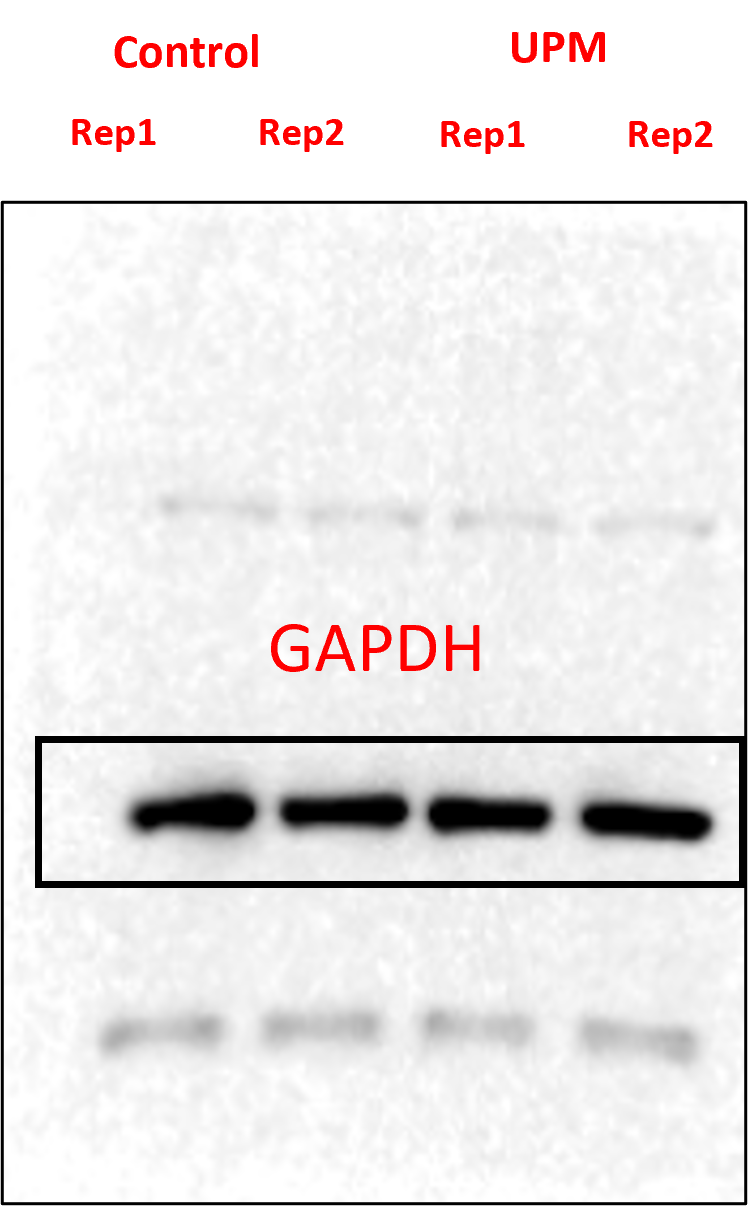

Supplement: Supplementary file 4 — Source data Fig. 3 [file 44321_2026_403_MOESM4_ESM.zip › C/GAPDH.tif]

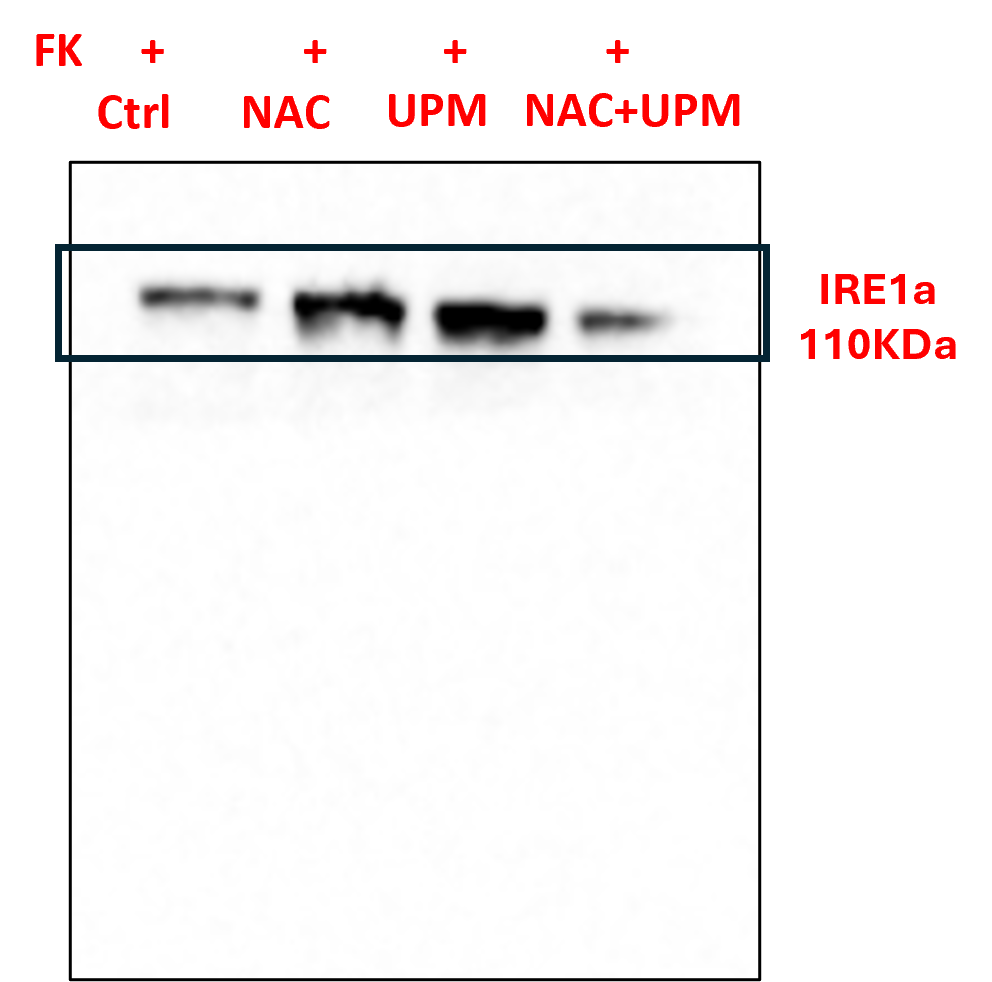

Supplement: Supplementary file 4 — Source data Fig. 3 [file 44321_2026_403_MOESM4_ESM.zip › C/IRE1a(NAC).tif]

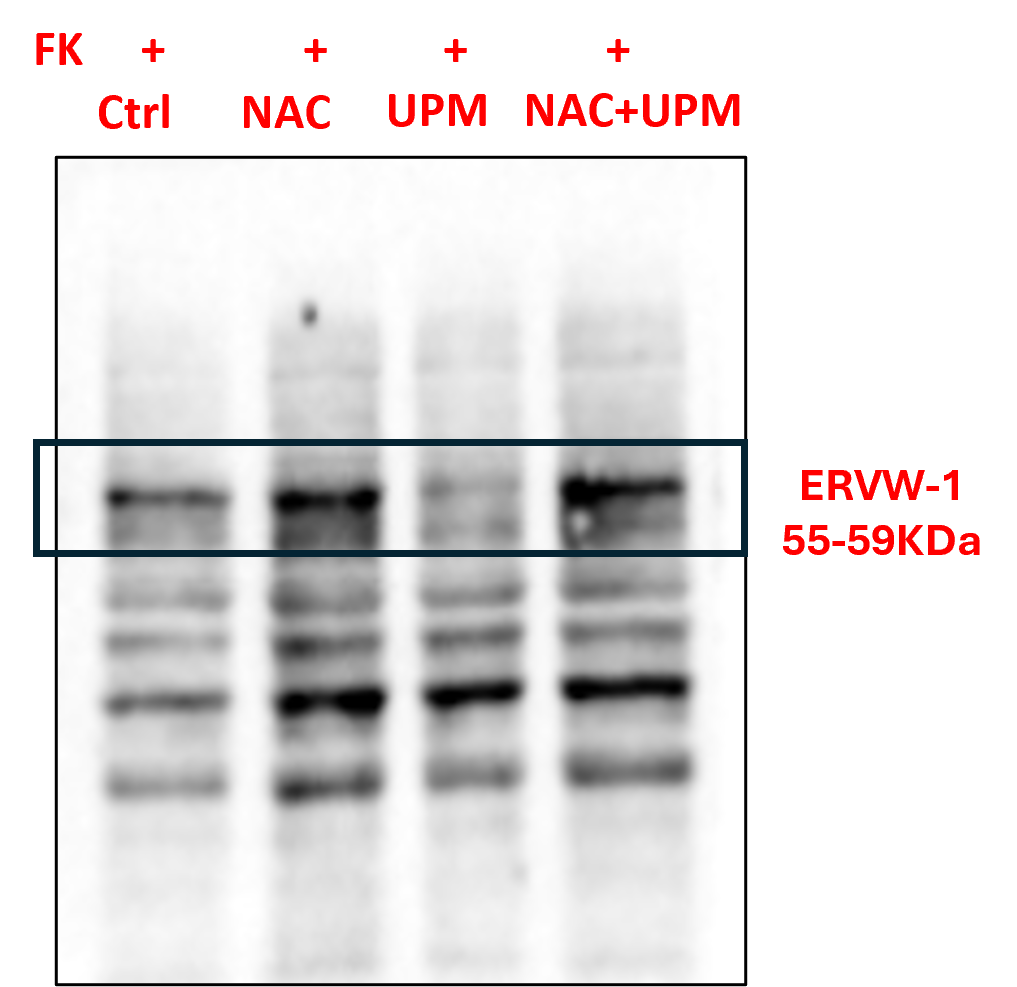

Supplement: Supplementary file 4 — Source data Fig. 3 [file 44321_2026_403_MOESM4_ESM.zip › C/Syncytin-1 (NAC).tif]

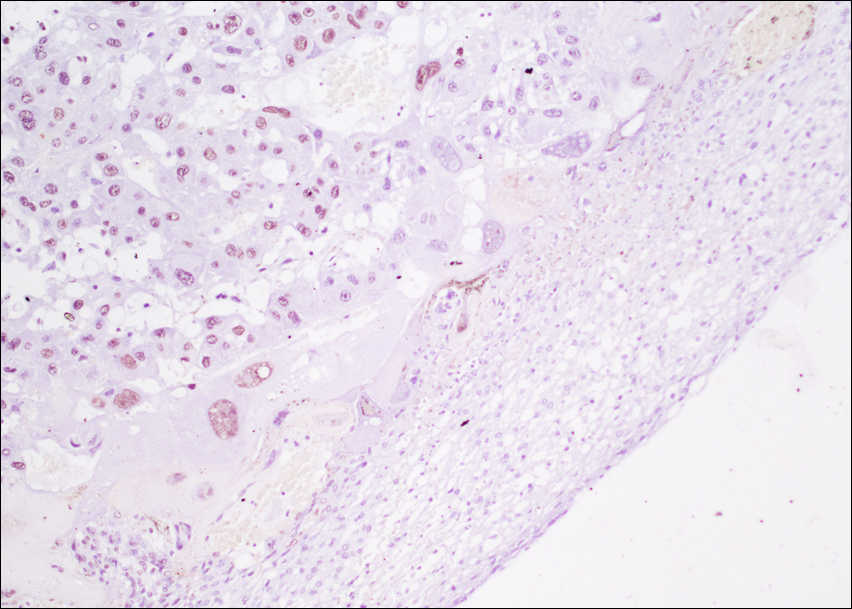

Supplement: Supplementary file 6 — Source data Fig. 5 [file 44321_2026_403_MOESM6_ESM.zip › C/Control1 10x.tif]

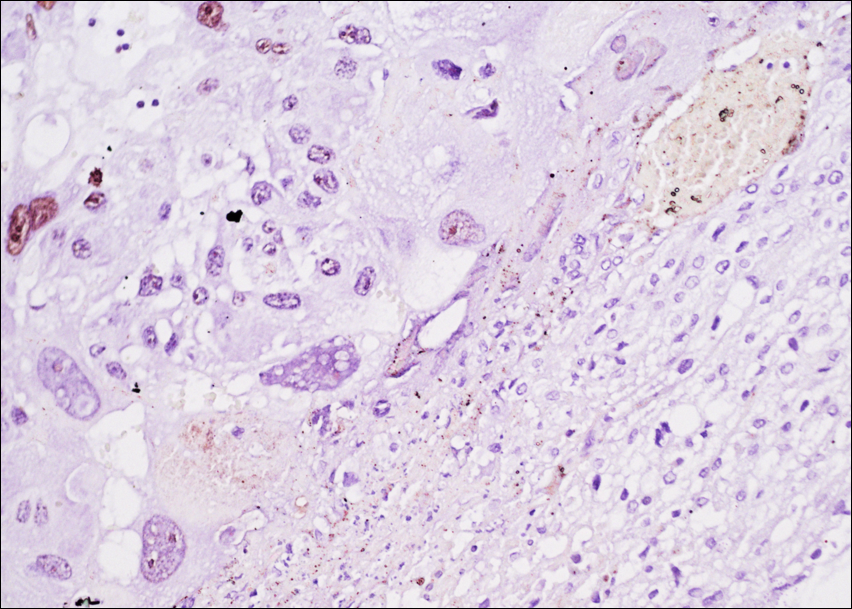

Supplement: Supplementary file 6 — Source data Fig. 5 [file 44321_2026_403_MOESM6_ESM.zip › C/Control1 20x.tif]

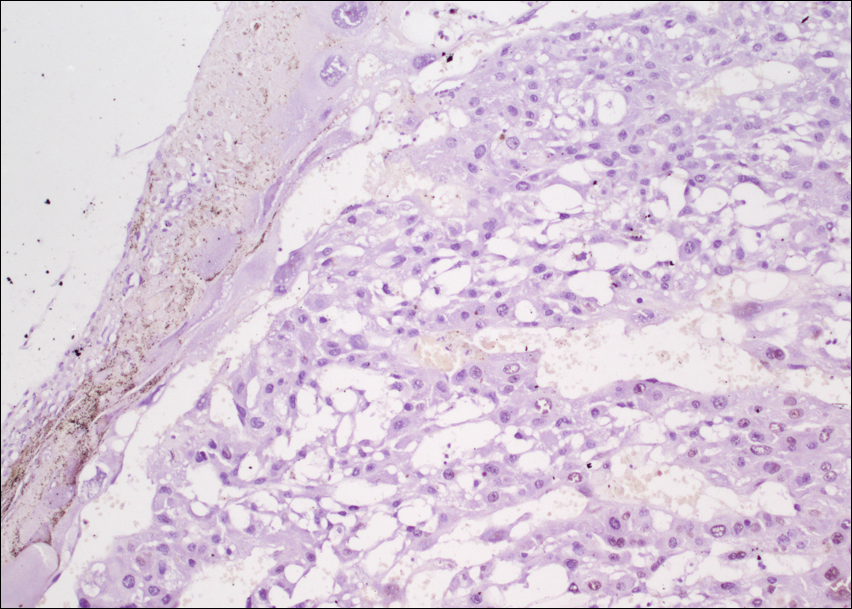

Supplement: Supplementary file 6 — Source data Fig. 5 [file 44321_2026_403_MOESM6_ESM.zip › C/Control2 10x.tif]

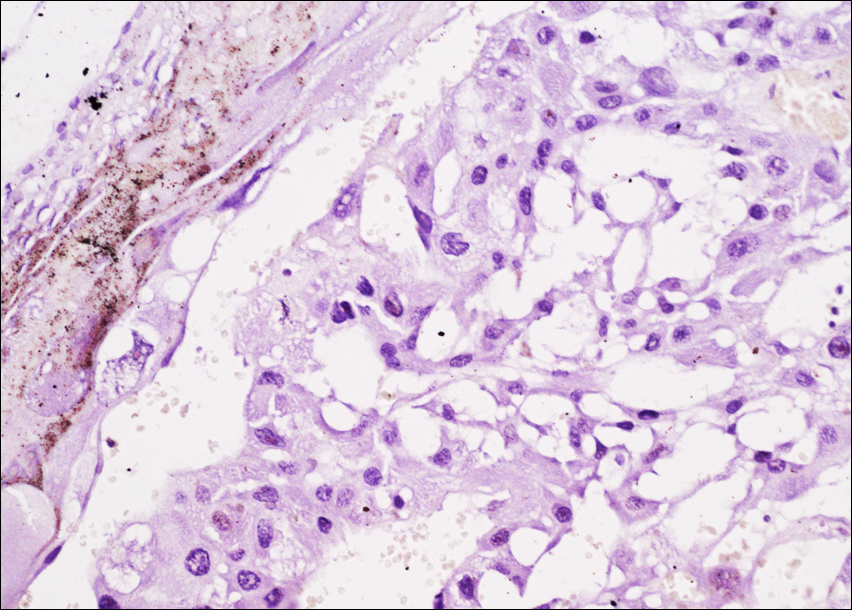

Supplement: Supplementary file 6 — Source data Fig. 5 [file 44321_2026_403_MOESM6_ESM.zip › C/Control2 20x.tif]

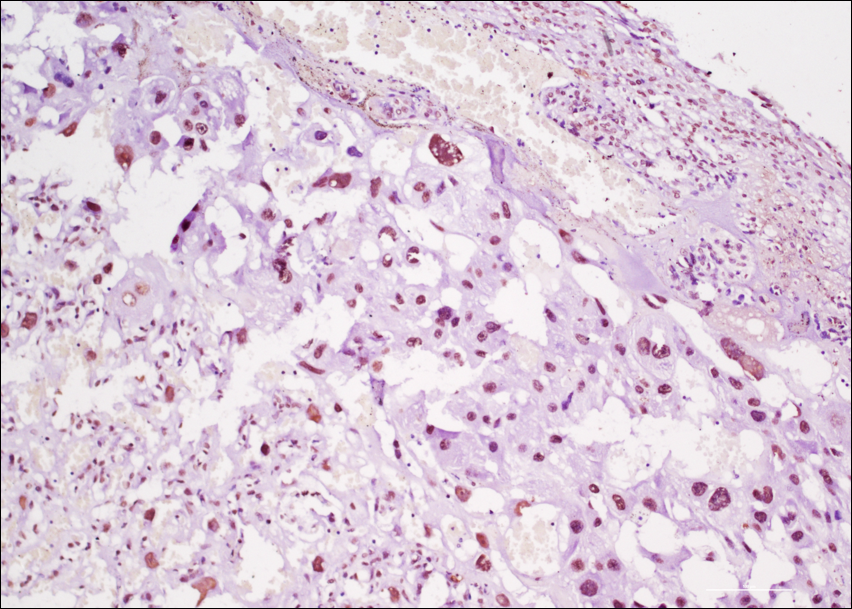

Supplement: Supplementary file 6 — Source data Fig. 5 [file 44321_2026_403_MOESM6_ESM.zip › C/UPM1 10x.tif]

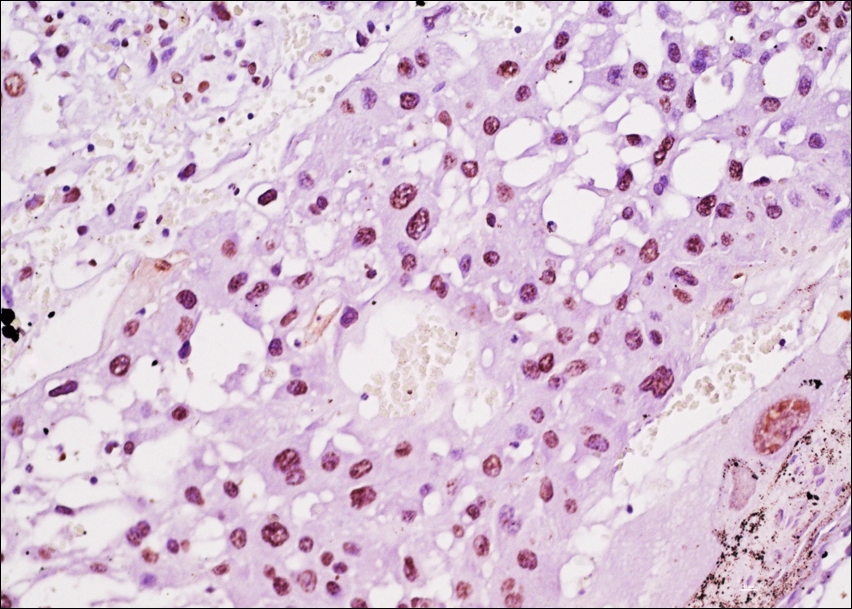

Supplement: Supplementary file 6 — Source data Fig. 5 [file 44321_2026_403_MOESM6_ESM.zip › C/UPM1 20x.tif]

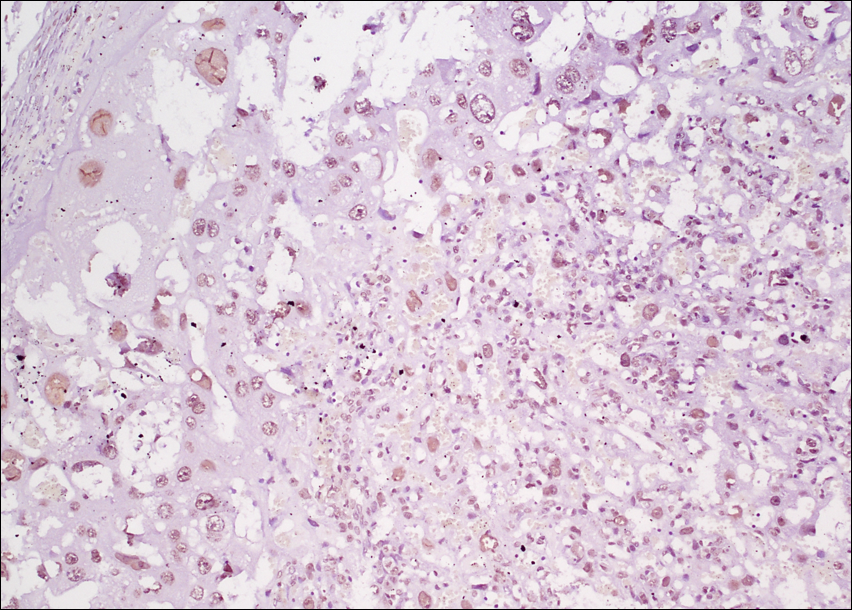

Supplement: Supplementary file 6 — Source data Fig. 5 [file 44321_2026_403_MOESM6_ESM.zip › C/UPM2 10X.tif]

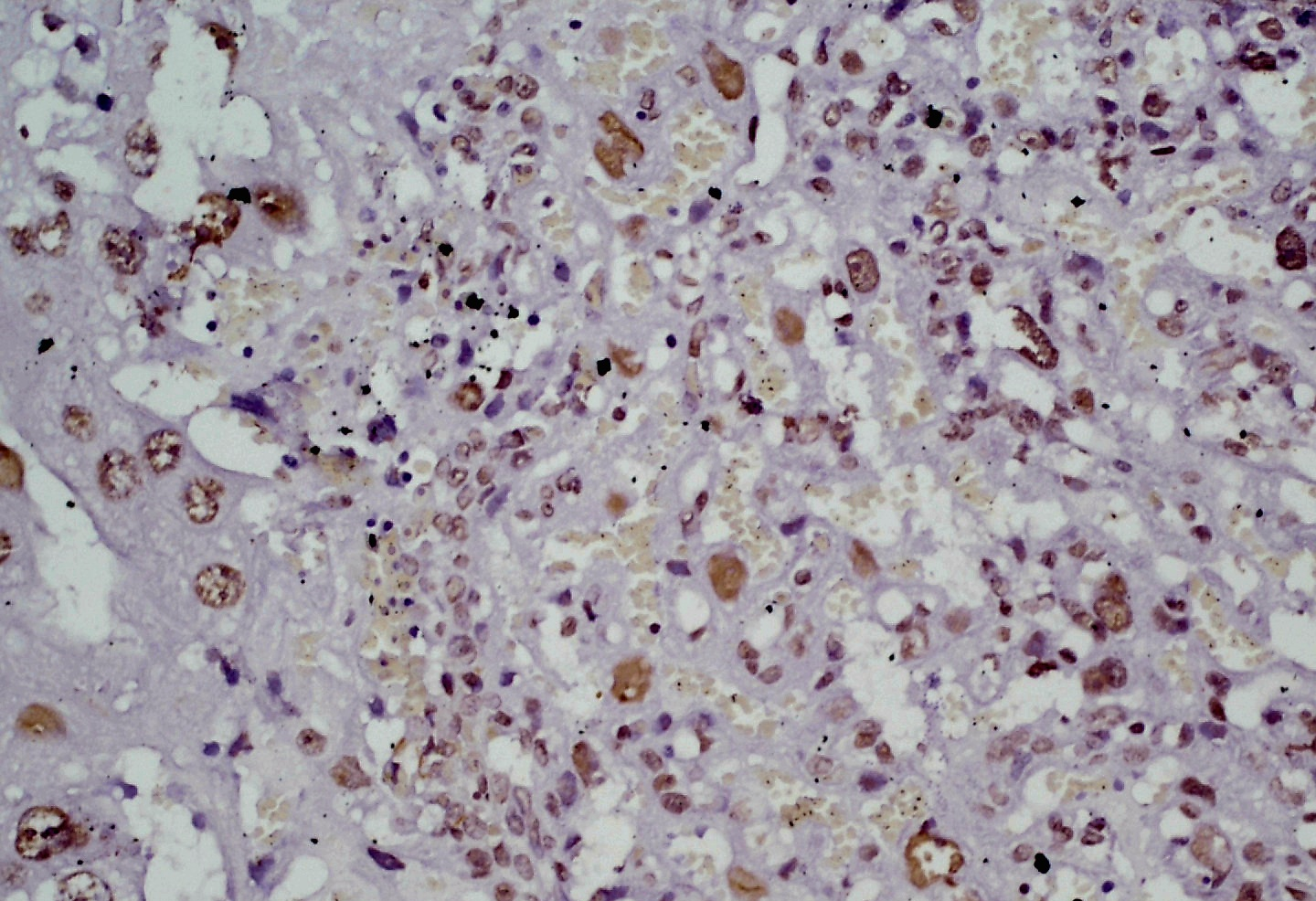

Supplement: Supplementary file 6 — Source data Fig. 5 [file 44321_2026_403_MOESM6_ESM.zip › C/UPM2 20x.tiff]

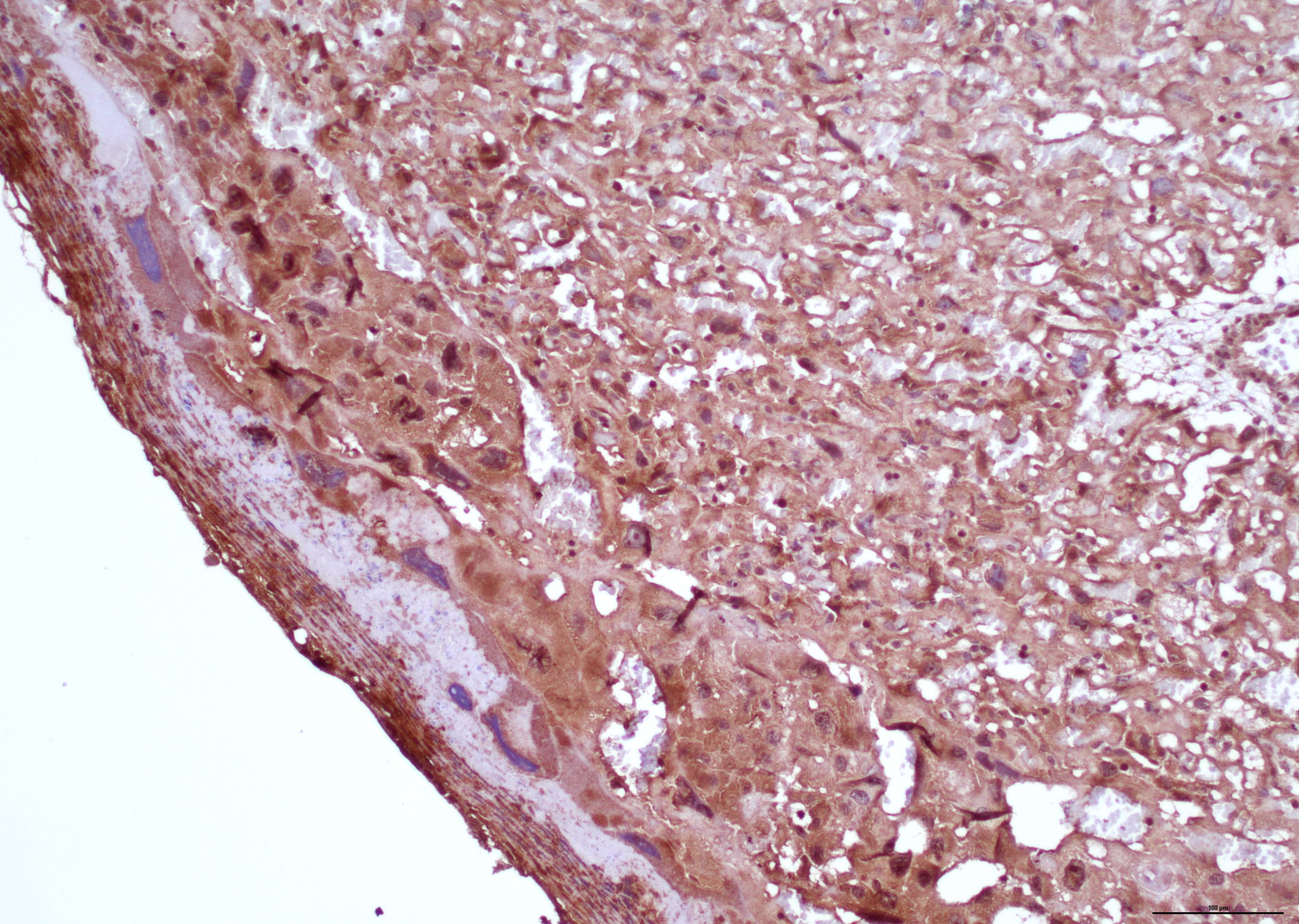

Supplement: Supplementary file 8 — Source data Fig. 7 [file 44321_2026_403_MOESM8_ESM.zip › C/Control BR1 10X.tif]

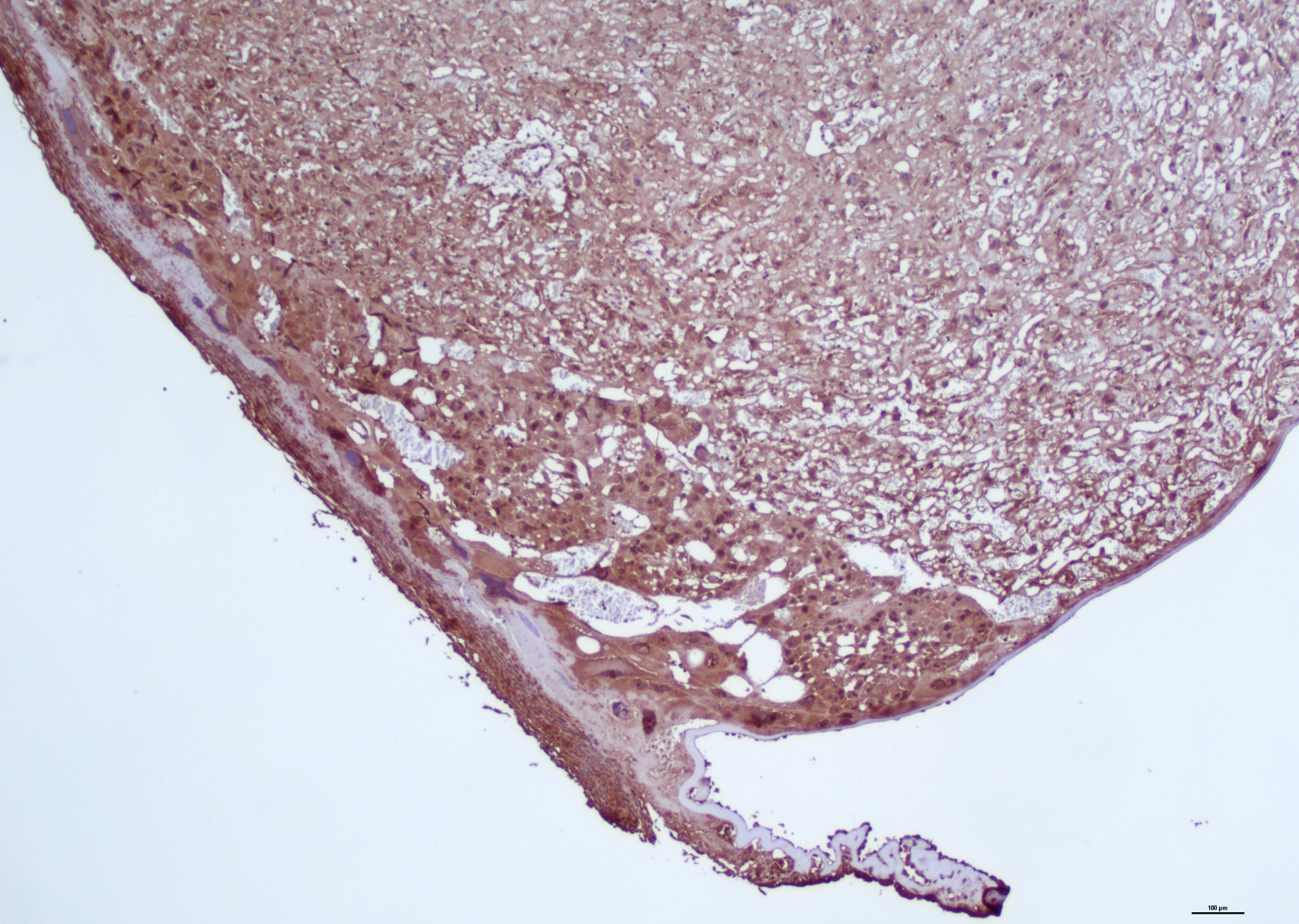

Supplement: Supplementary file 8 — Source data Fig. 7 [file 44321_2026_403_MOESM8_ESM.zip › C/Control BR1 4X.tif]

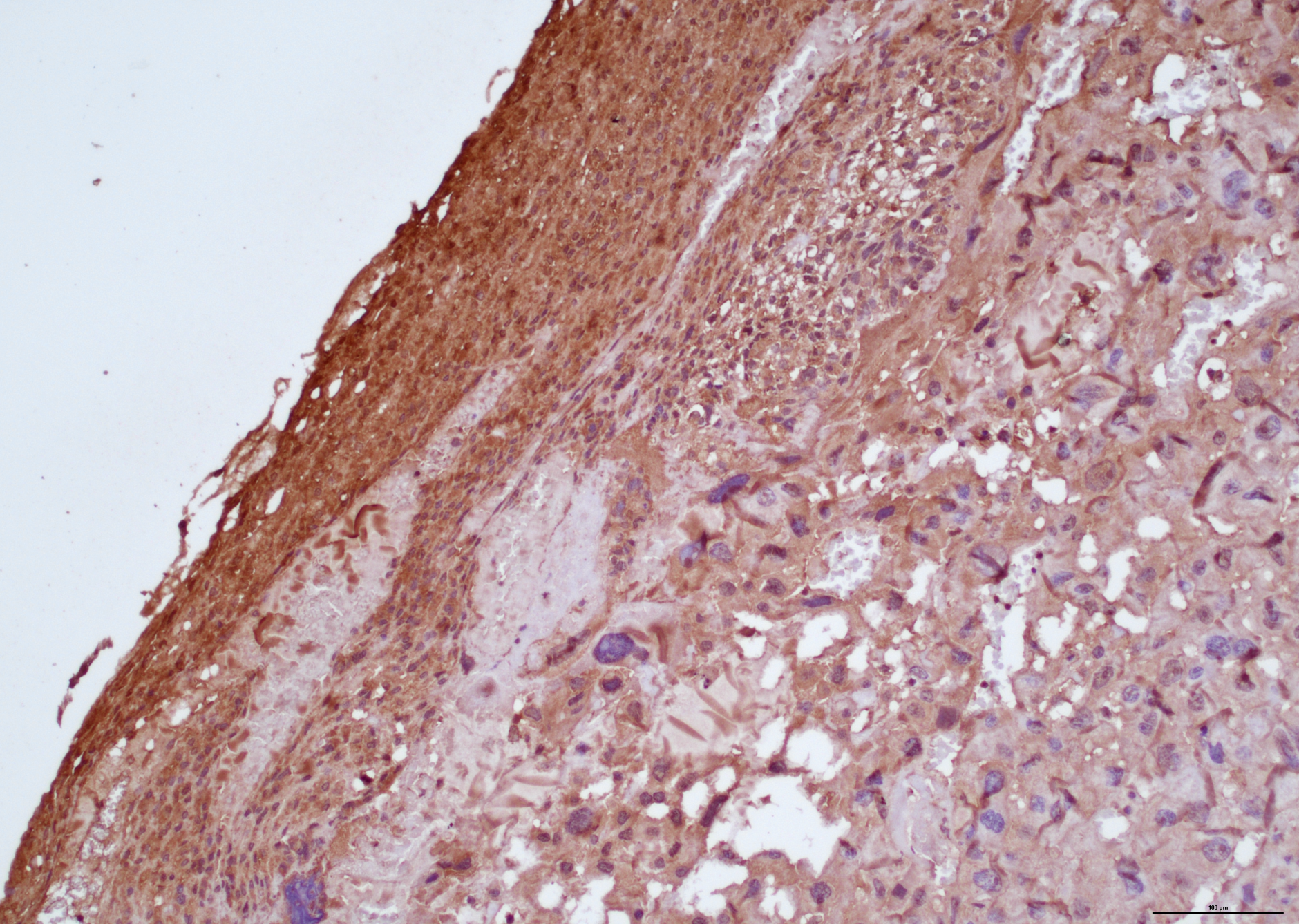

Supplement: Supplementary file 8 — Source data Fig. 7 [file 44321_2026_403_MOESM8_ESM.zip › C/Control BR2 10X.tif]

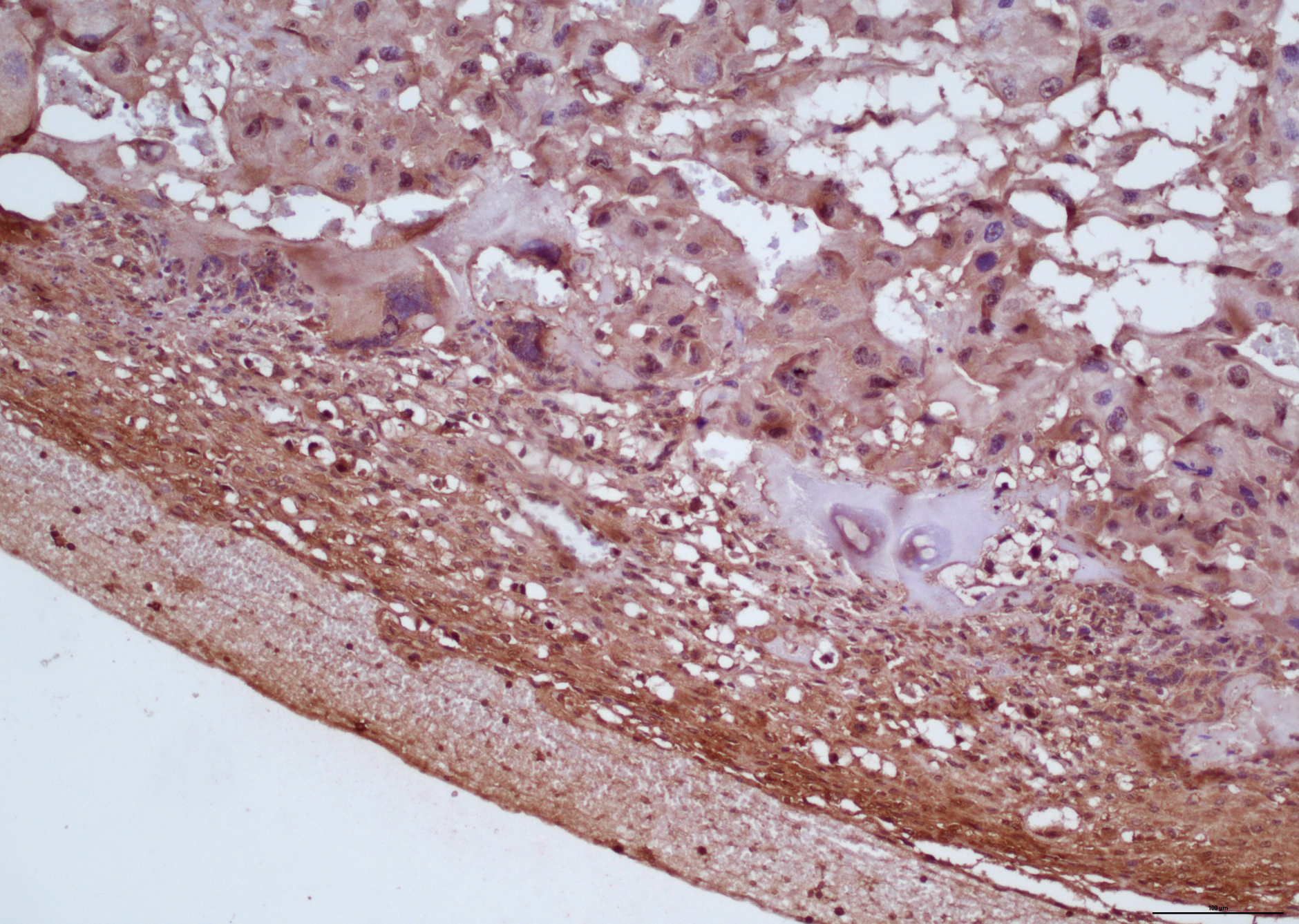

Supplement: Supplementary file 8 — Source data Fig. 7 [file 44321_2026_403_MOESM8_ESM.zip › C/Control BR3 10X.tif]

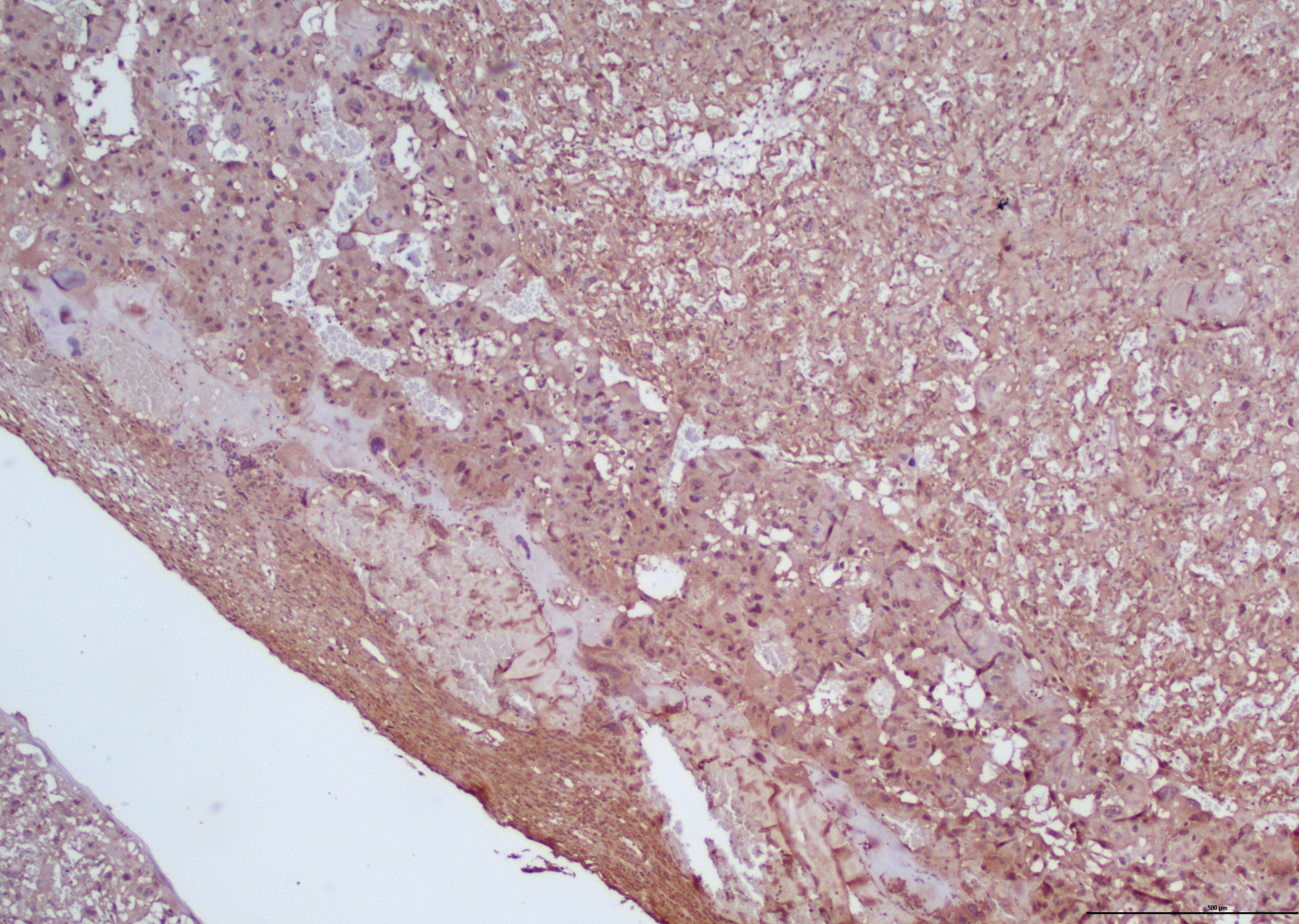

Supplement: Supplementary file 8 — Source data Fig. 7 [file 44321_2026_403_MOESM8_ESM.zip › C/Control BR3 4X.tif]

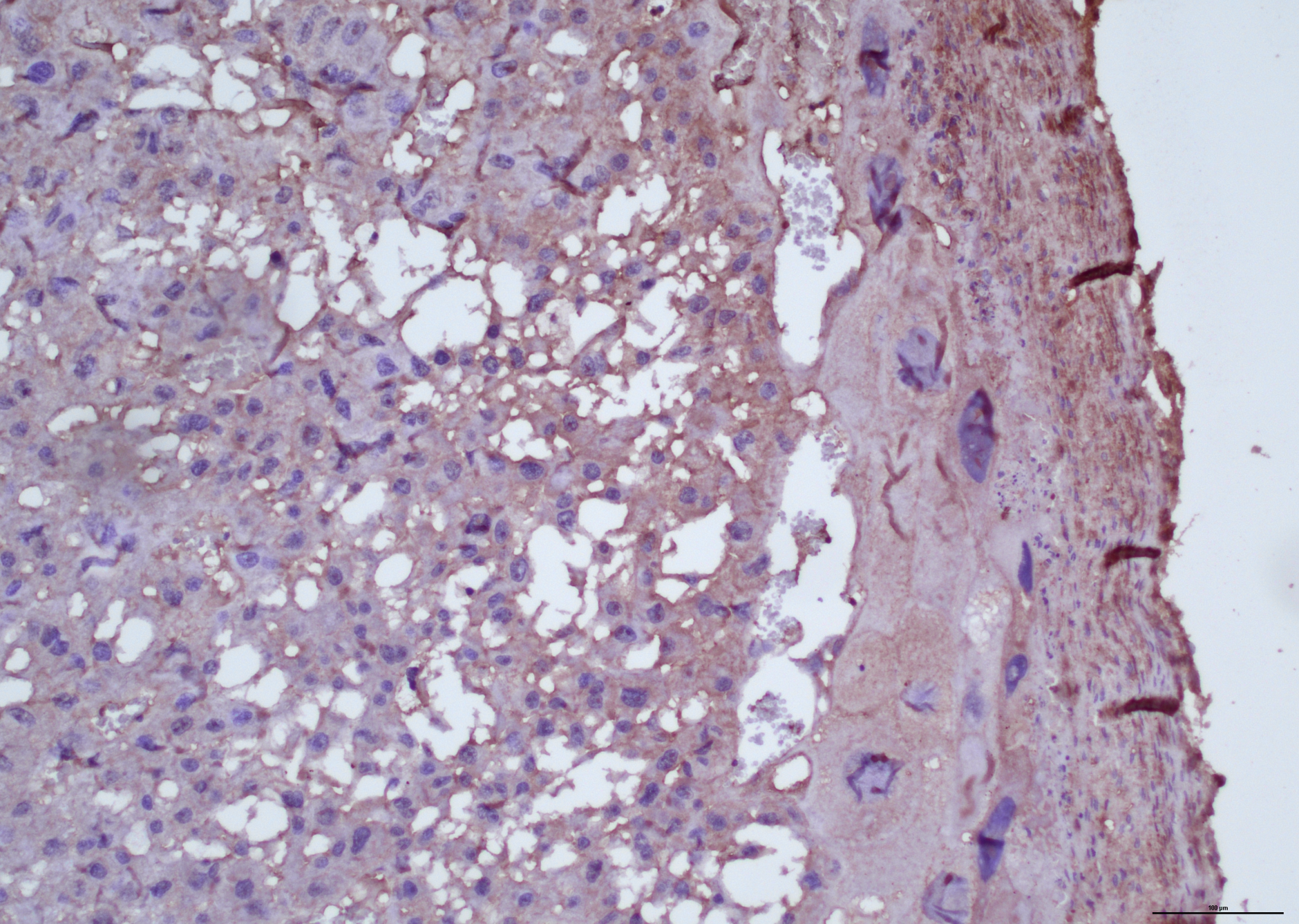

Supplement: Supplementary file 8 — Source data Fig. 7 [file 44321_2026_403_MOESM8_ESM.zip › C/UPM BR1 10X.tif]

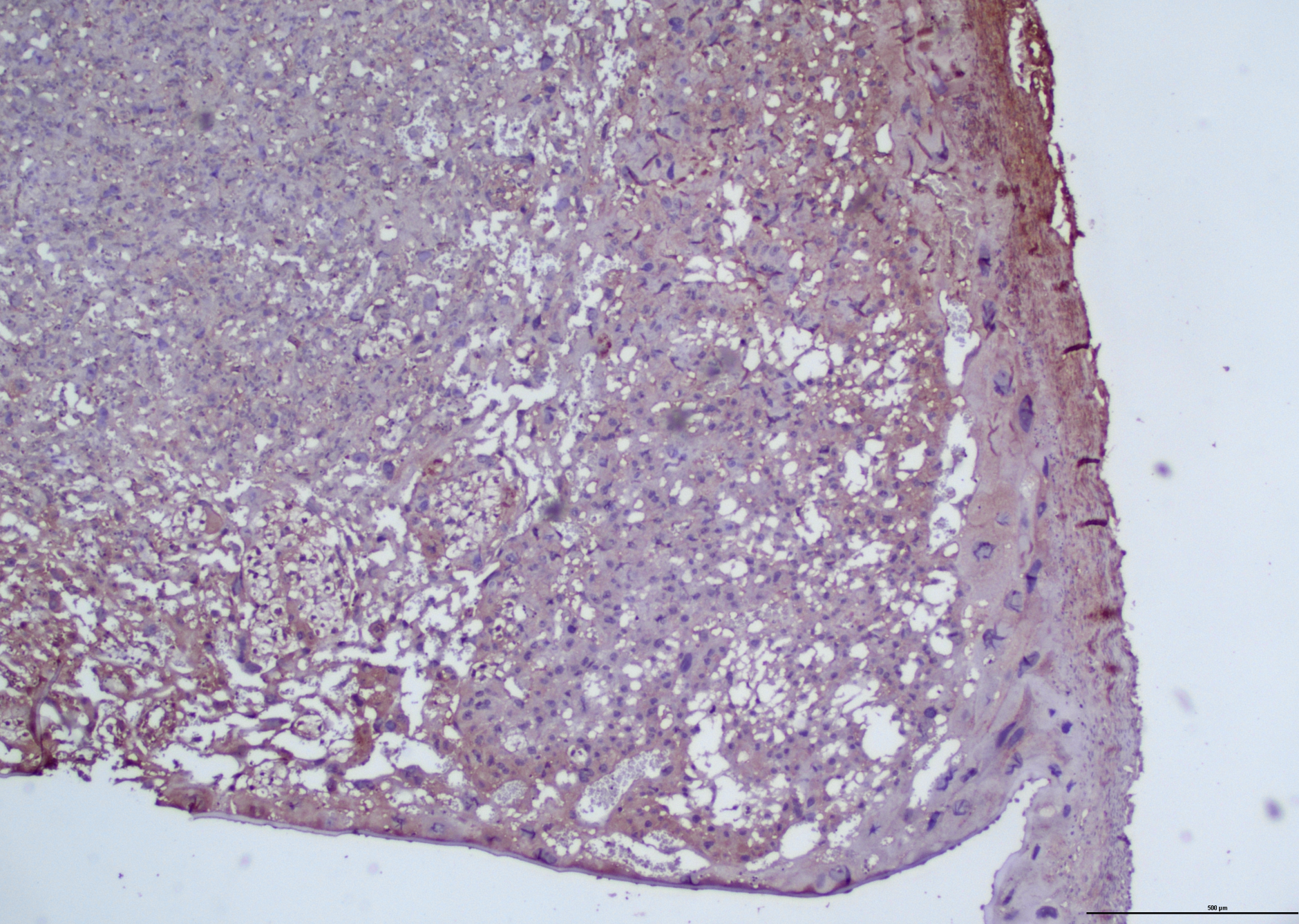

Supplement: Supplementary file 8 — Source data Fig. 7 [file 44321_2026_403_MOESM8_ESM.zip › C/UPM BR1 4X.tif]

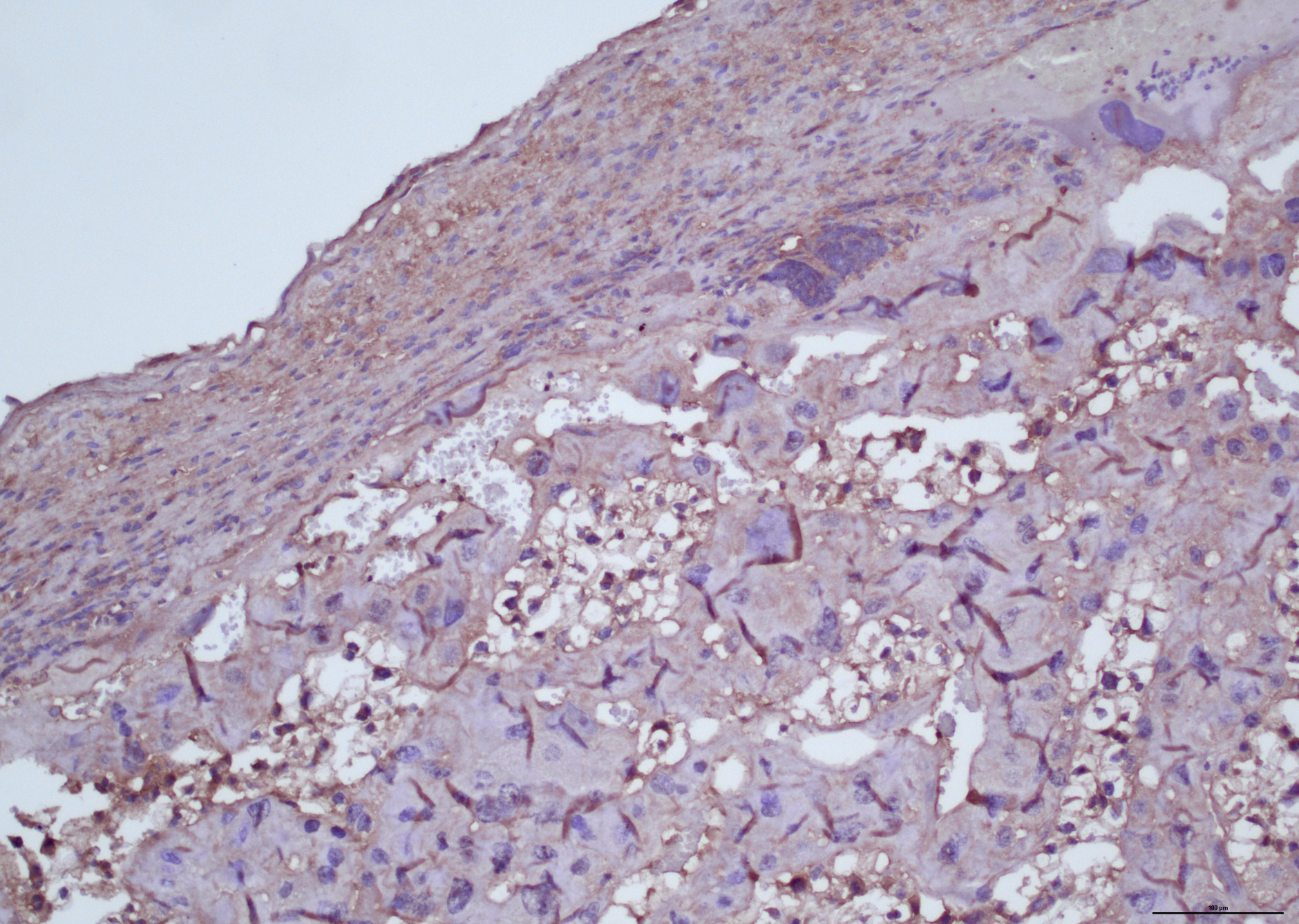

Supplement: Supplementary file 8 — Source data Fig. 7 [file 44321_2026_403_MOESM8_ESM.zip › C/UPM BR2 10X.tif]

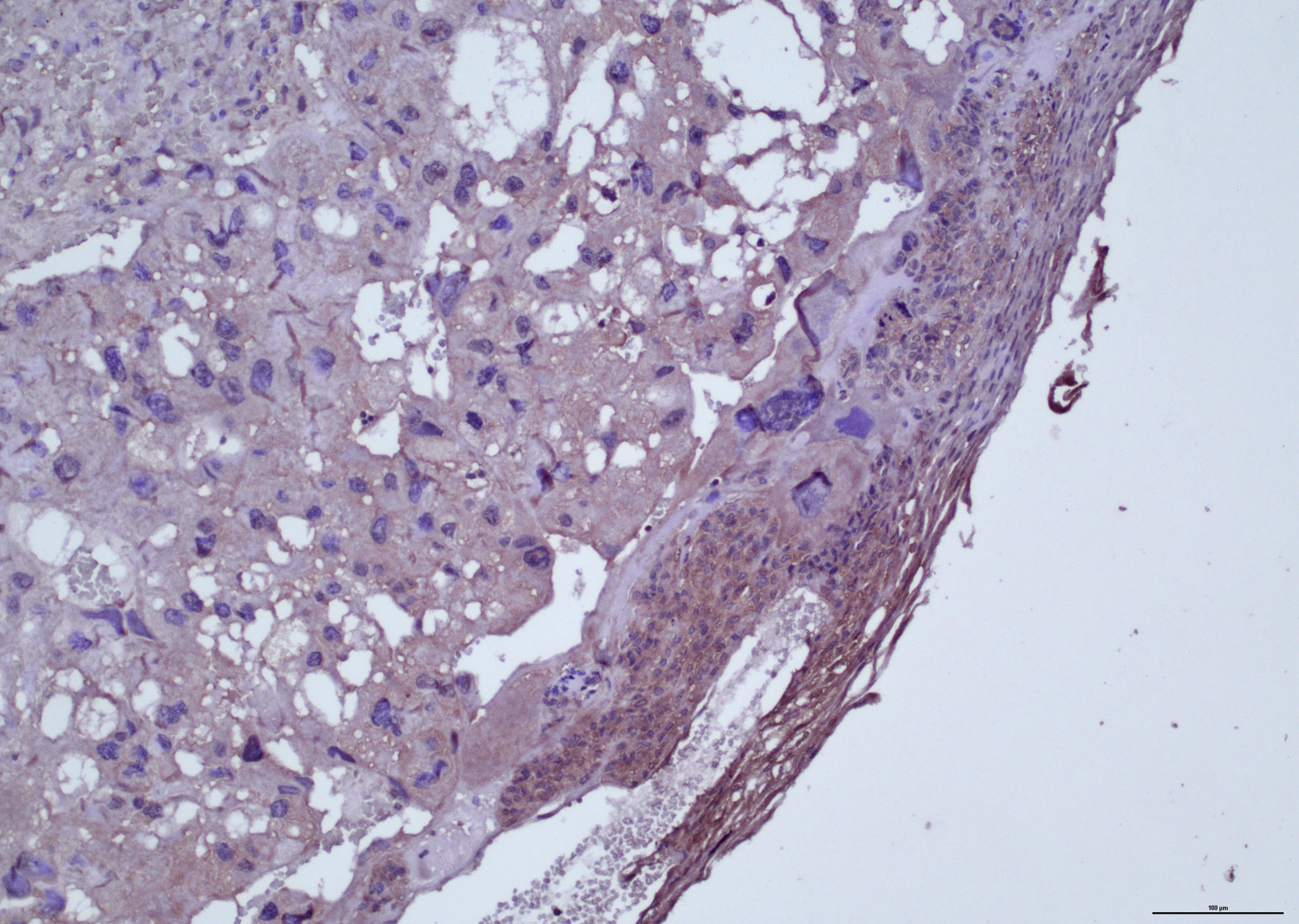

Supplement: Supplementary file 8 — Source data Fig. 7 [file 44321_2026_403_MOESM8_ESM.zip › C/UPM BR3 10X.tif]

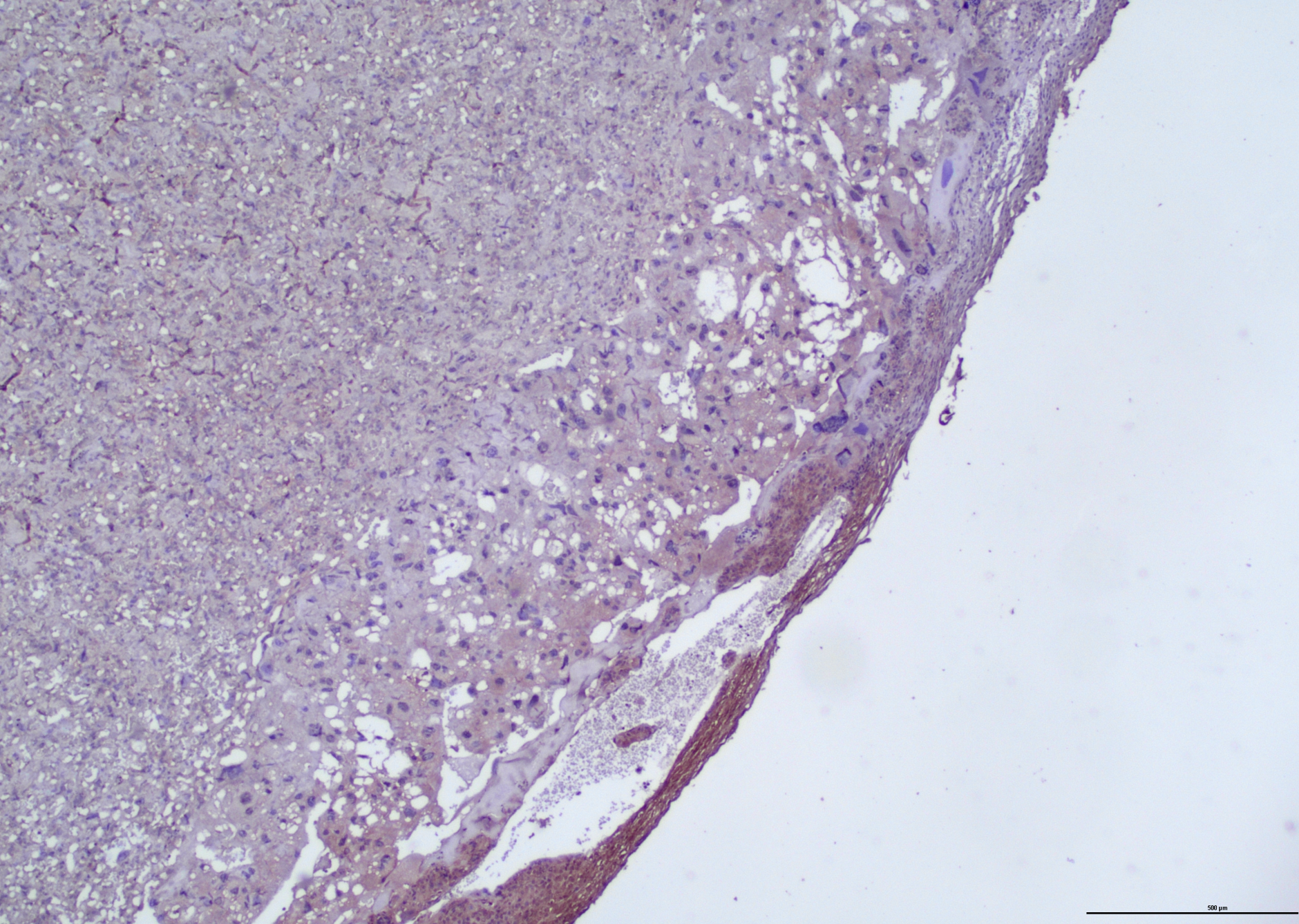

Supplement: Supplementary file 8 — Source data Fig. 7 [file 44321_2026_403_MOESM8_ESM.zip › C/UPM BR3 4X.tif]

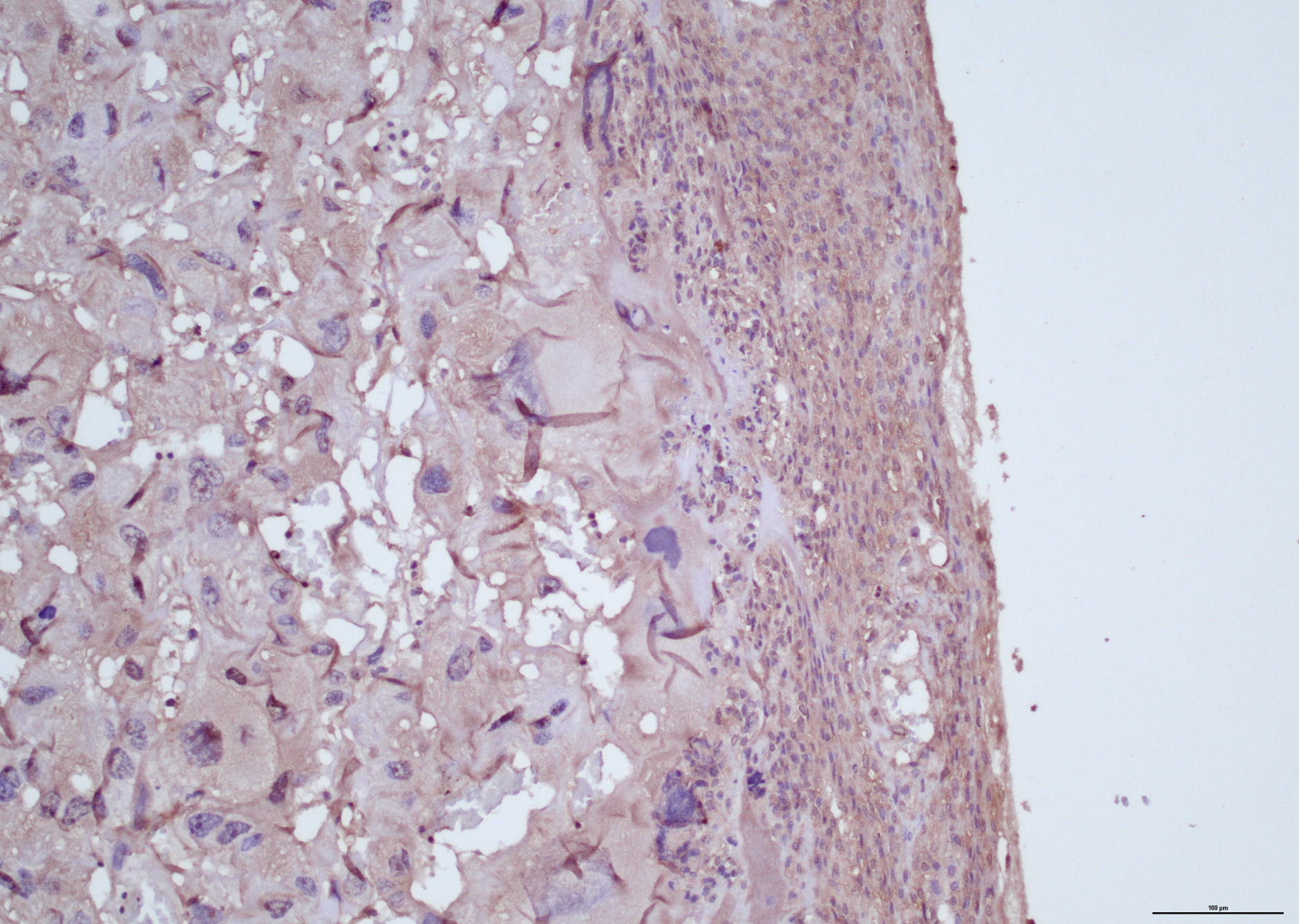

Supplement: Supplementary file 8 — Source data Fig. 7 [file 44321_2026_403_MOESM8_ESM.zip › C/UPM BR4 10X.tif]

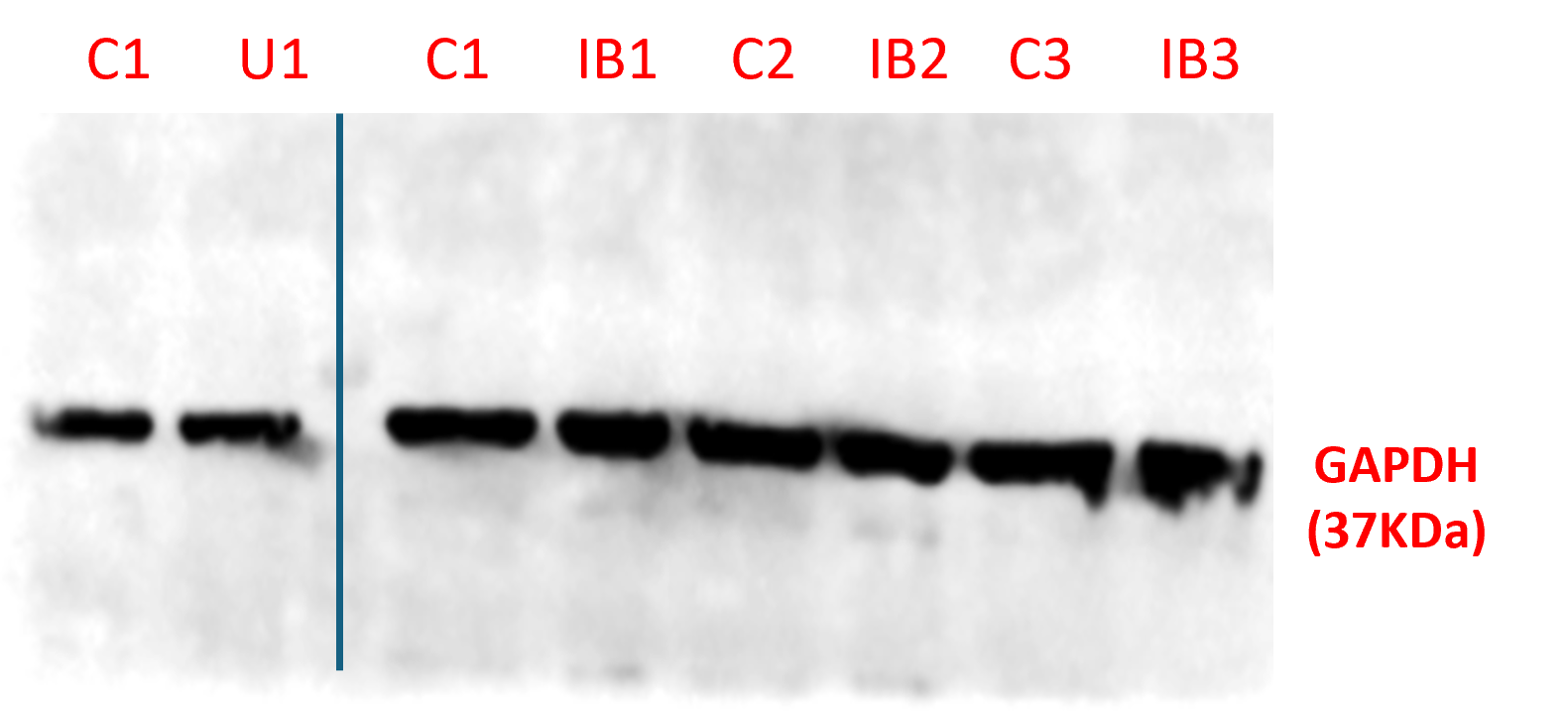

Supplement: Supplementary file 8 — Source data Fig. 7 [file 44321_2026_403_MOESM8_ESM.zip › D/GAPDH.tif]

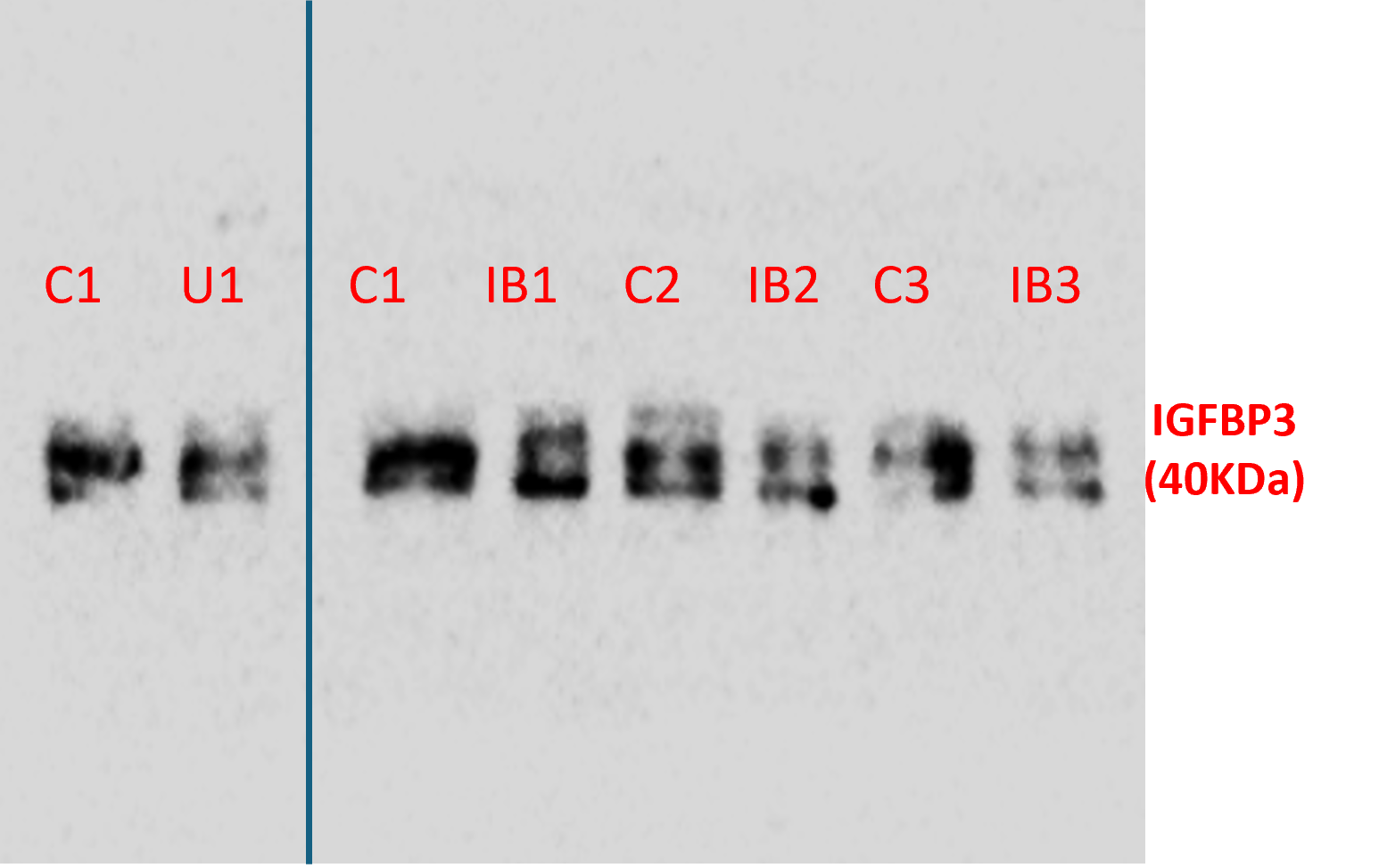

Supplement: Supplementary file 8 — Source data Fig. 7 [file 44321_2026_403_MOESM8_ESM.zip › D/IGFBP3.tif]

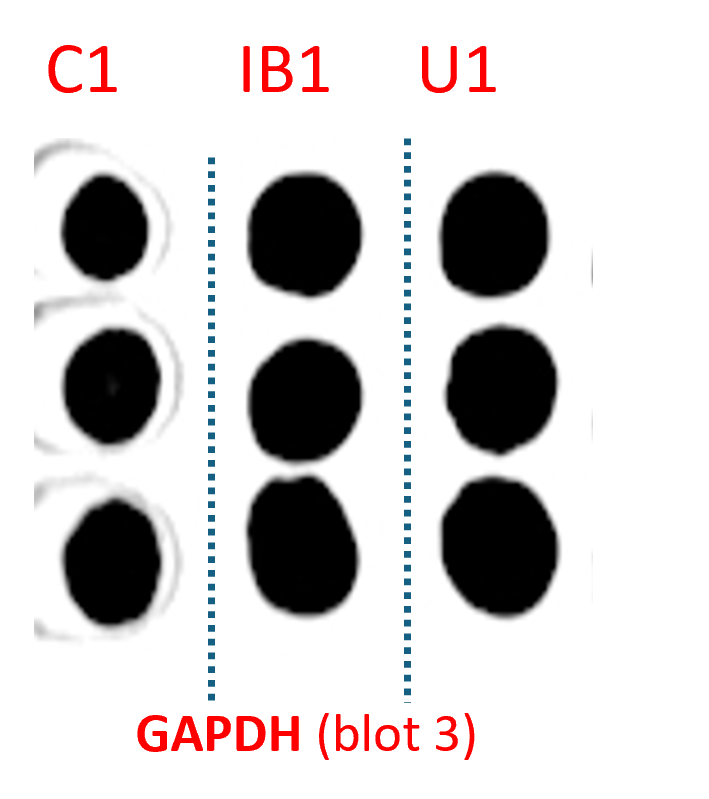

Supplement: Supplementary file 8 — Source data Fig. 7 [file 44321_2026_403_MOESM8_ESM.zip › E/GAPDH blot 1.tif]

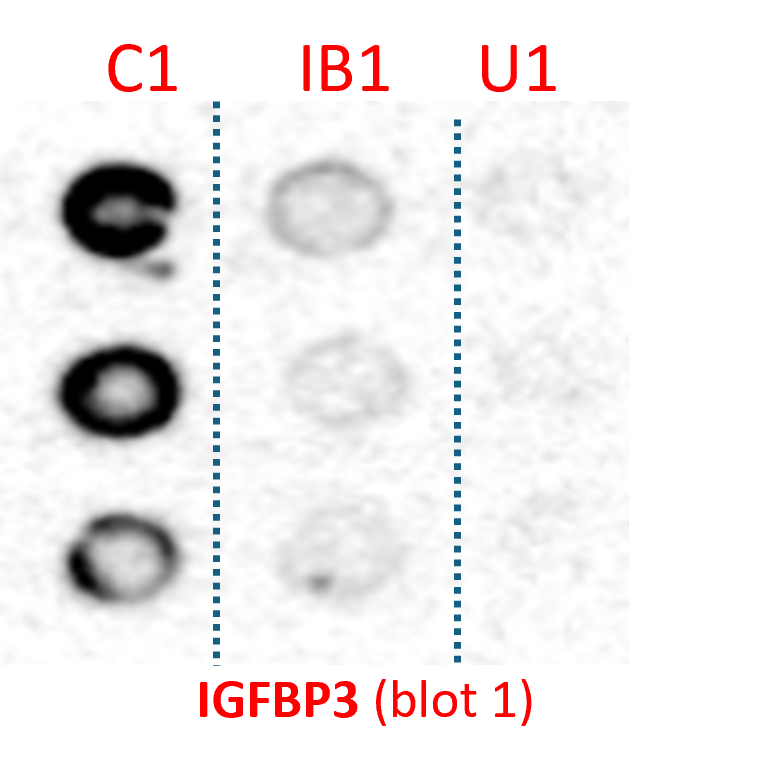

Supplement: Supplementary file 8 — Source data Fig. 7 [file 44321_2026_403_MOESM8_ESM.zip › E/IGFBP3 blot 1.tif]

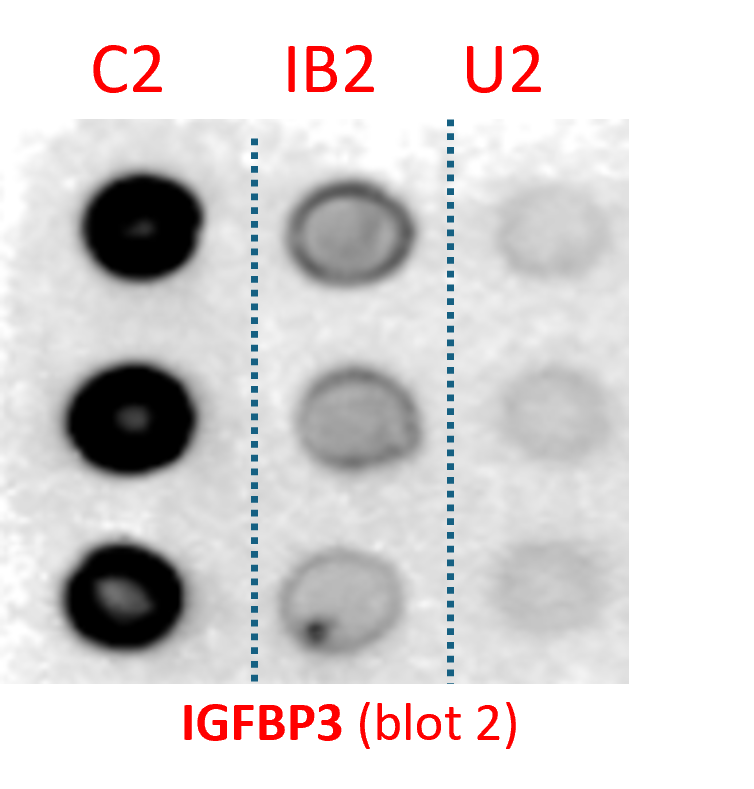

Supplement: Supplementary file 8 — Source data Fig. 7 [file 44321_2026_403_MOESM8_ESM.zip › E/IGFBP3 blot 2.tif]

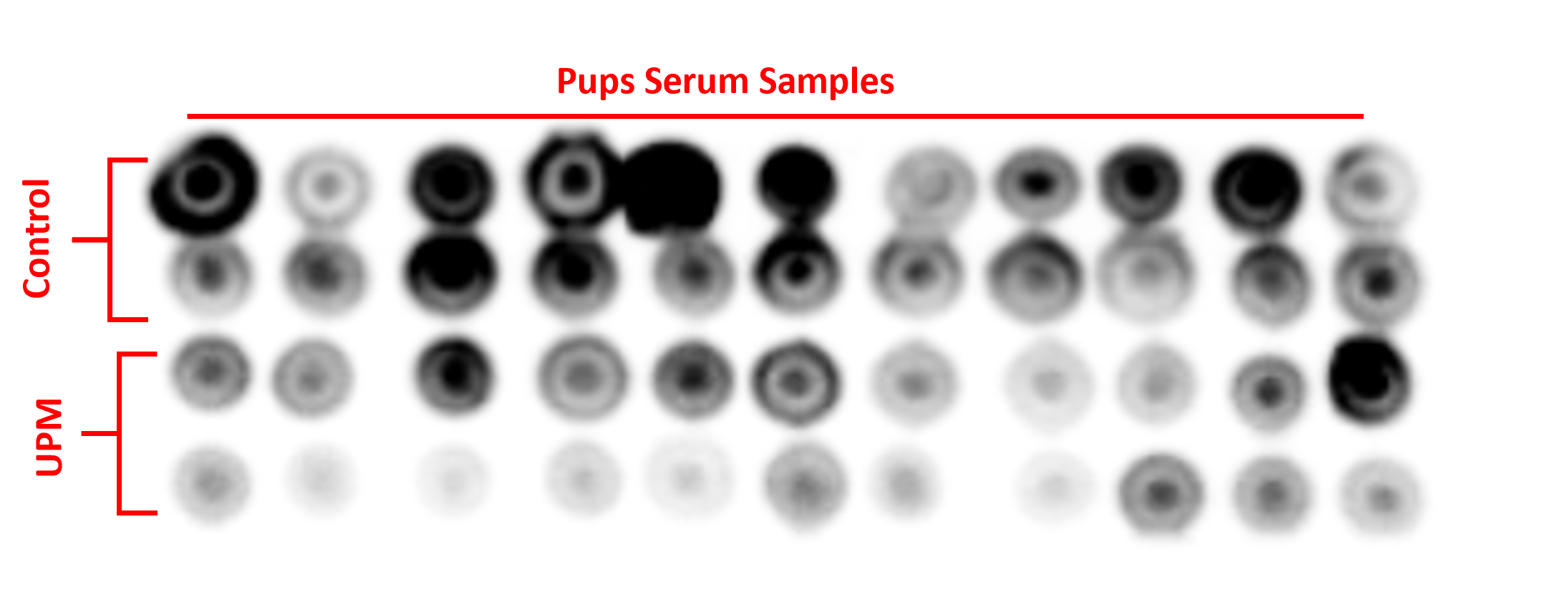

Supplement: Supplementary file 8 — Source data Fig. 7 [file 44321_2026_403_MOESM8_ESM.zip › G/IGFBP3 dot-blot Pups Serum.tif]
