# Supplementary material for: IGFBP3 repression driven by inflammation links air pollution to placental and developmental defects
Source: EMBO Mol Med. 2026 Mar 24;18(5):1648–78. doi: 10.1038/s44321-026-00403-x (PMC13179339; doi:10.1038/s44321-026-00403-x)
Supplement: Supplementary file 9 — Expanded View Figures [file 44321_2026_403_MOESM9_ESM.pdf]

Expanded View Figures

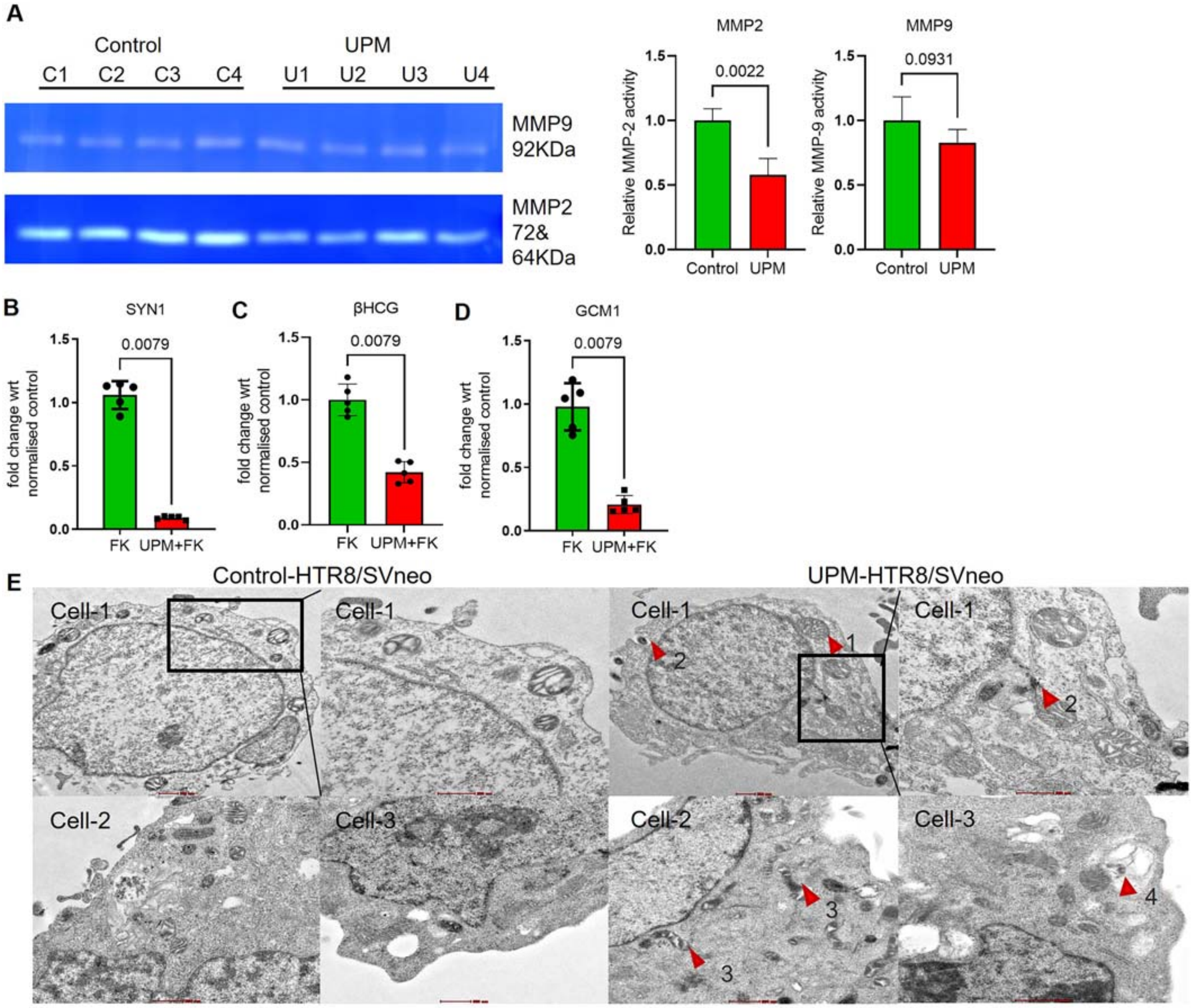

Feature	Control	UPM-treated
**Mitochondrial morphology**	Mostly intact with dense cristae and electron-dense matrix	Many swollen, with loss or disruption of cristae; some show ruptured outer membranes;
**Vacuolation**	Minimal or absent	Extensive vacuolation, suggestive of autophagic or lysosomal structures
**Signs of autophagy**	Absent or minimal	Frequent autophagic vesicles
**Overall cell integrity**	Maintained, with preserved organelle ultrastructure	Compromised, with advanced organelle degeneration and possible late-stage cell injury

Arrow indicating

1. Enlarged Mitochondria
2. Foreign particles (could be particulate matter)
3. Disoriented mitochondria
4. Vacuolar structure (double membrane autophagosome)

Figure EV1. UPM exposure disrupts trophoblast invasive enzymes, syncytialization markers, and cellular ultrastructure.

(**A**) Gelatin zymography analysis showing a marked reduction in MMP2 and MMP9 gelatinolytic activity in UPM-treated HTR8/SVneo cells compared with untreated controls ($n = 4$). L: ladder; C1–C4: Control replicates; U1–U4: UPM-exposed replicates. Band intensities were quantified by densitometry and normalized to total protein load. Data are presented as mean ± SD and analyzed using two-tailed Mann-Whitney test. (**B–D**) RT–qPCR analysis demonstrating significant downregulation of *Syncytin-1*, *β-hCG*, and *GCM1* mRNA levels in BeWo cells following UPM stimulation in the presence of forskolin ($n = 5$). Fold change values ($2^{-\Delta\Delta Ct}$) were normalized to GAPDH expression. Data are presented as mean ± SD and analyzed using two-tailed Mann-Whitney test. (**E**) Transmission electron microscopy (TEM) of HTR8/SVneo cells ($n = 3$) showing pronounced ultrastructural alterations following UPM exposure. Control cells displayed intact mitochondrial cristae, minimal vacuolation, and preserved organelle integrity. In contrast, UPM-treated cells exhibited swollen mitochondria, disrupted cristae, extensive cytoplasmic vacuolation, and frequent autophagic vesicles (red arrowheads). A comparative summary of morphological features between control and UPM-treated cells is provided. Scale bar equal 500 nm.

© The Author(s)

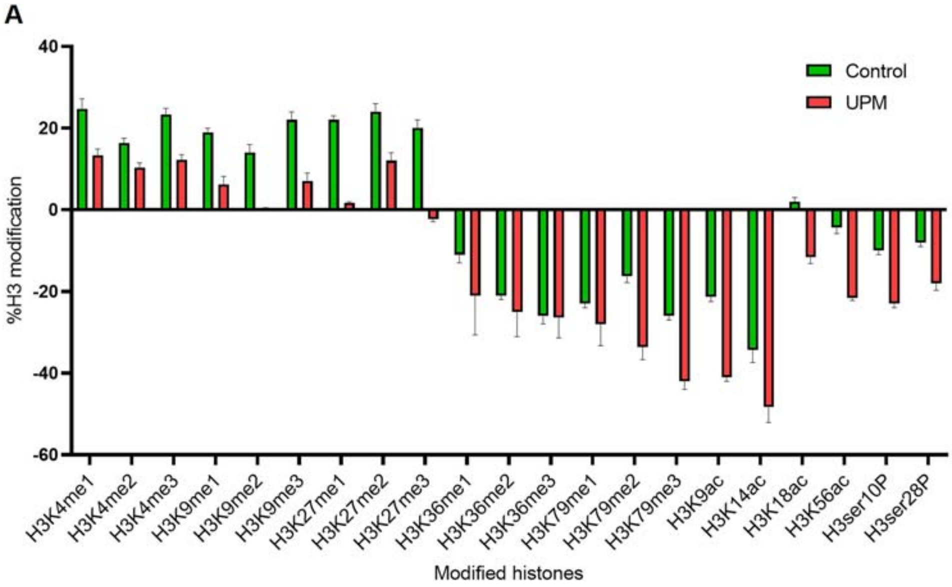

Modified H3	P- value
H3K4me1	0.0286
H3K4me2	0.0286
H3K4me3	0.0286
H3K9me1	0.0286
H3K9me2	0.0159
H3K9me3	0.0286
H3K27me1	0.0286

Modified H3	P- value
H3K27me2	0.0159
H3K27me3	0.0159
H3K36me1	0.066
H3K36me2	0.3143
H3K36me3	0.3143
H3K79me1	0.1
H3K79me2	0.0286

Modified H3	P- value
H3K79me3	0.0286
H3K9ac	0.0286
H3K14ac	0.1
H3K18ac	0.0286
H3K56ac	0.0286
H3ser10P	0.0286
H3ser28P	0.0286

Figure EV2. Global Histone H3 modification profiling.

(**A**) UPM exposure induced substantial alterations in the global histone H3 modification landscape within HTR8/SVneo cells. Comprehensive analysis utilizing a Histone H3 Epigenetic Modification ELISA Panel quantified 21 distinct H3 modifications in nuclear extracts from control and UPM-treated trophoblast cells ($n = 4$). Comparative bar graph visualization displayed relative changes, with green bars representing control conditions and red bars indicating UPM treatment effects. Multiple histone modifications, encompassing both methylation and acetylation marks at critical lysine residues, exhibited significant upregulation or downregulation following UPM exposure. These findings demonstrate that urban particulate matter fundamentally disrupts chromatin modification patterns and potentially mediates epigenetic reprogramming of trophoblast cellular function. Data are presented as mean ± SD. Statistical significance was determined using an unpaired two-tailed Student's t-test.

 © The Author(s)

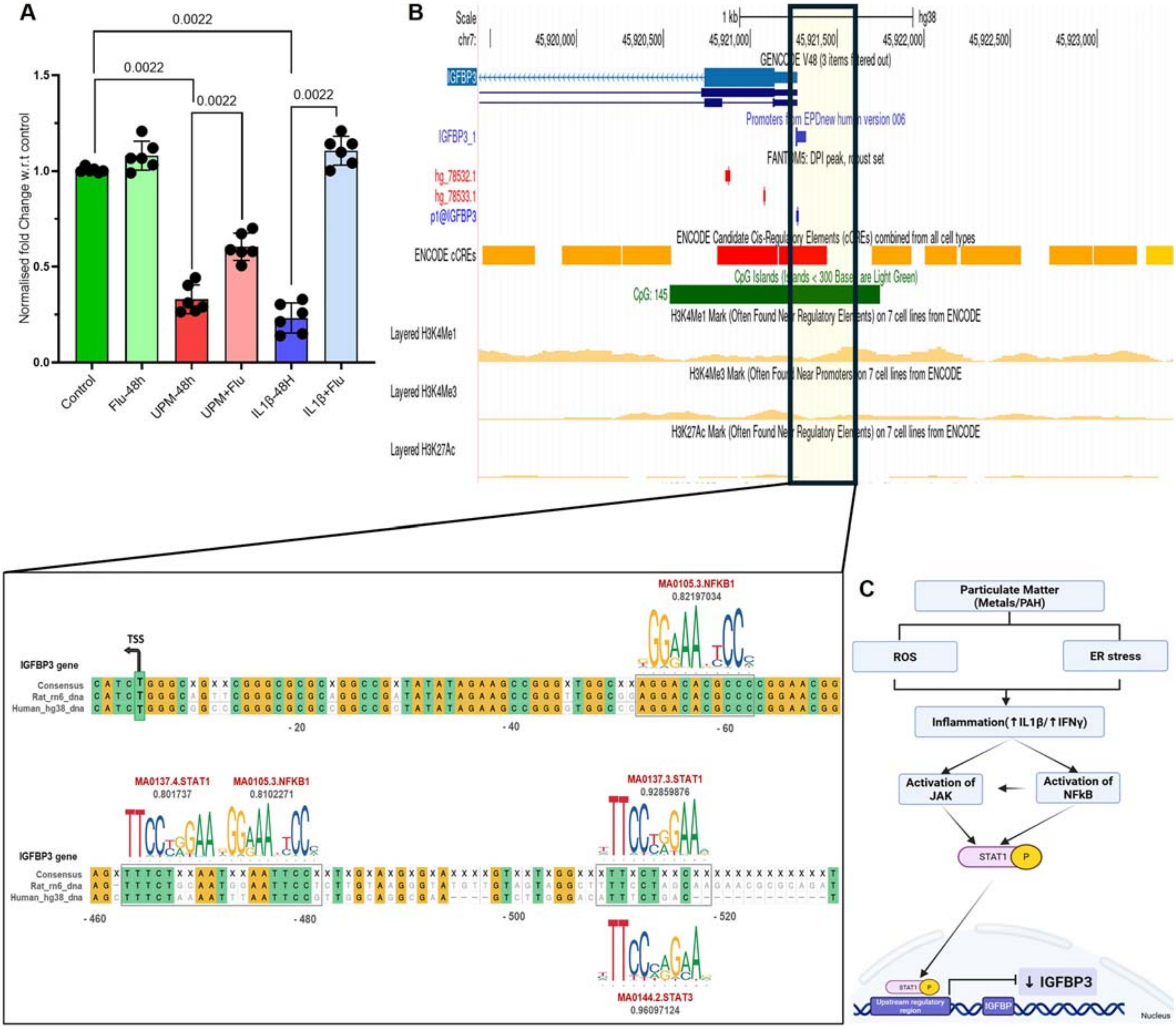

Figure EV3. Fludarabine prevents IGFBP3 repression and an in silico promoter analysis reveals conserved STAT1/NF-κB regulatory motifs.

(**A**) Effect of 48 h Fludarabine (Flu) (STAT1 inhibitor) on IGFBP3 expression under UPM and IL1β exposure: HTR8/SVneo cells were treated with Fludarabine (10 µM, 48 h) in the presence or absence of urban particulate matter (UPM; 50 µg/mL) or recombinant IL1β (10 ng/mL). QPCR analysis demonstrated that UPM and IL1β exposure significantly downregulated IGFBP3 mRNA levels, whereas Fludarabine treatment prevented this repression, restoring IGFBP3 expression close to basal levels. Normalized fold change in IGFBP3 expression was calculated using the $2^{-\Delta\Delta Ct}$ method, with GAPDH serving as the internal housekeeping control. Data represents mean ± SD from six independent experiments ($n = 6$). Statistical analysis was performed using one-way ANOVA. (**B**) Predicted transcription factor binding motifs in IGFBP3 promoter architecture: Inflammatory-responsive IGFBP3 transcriptional control appears to be orchestrated via STAT1 and NF-κB regulatory pathways, given the presence of computationally identified cognate binding sequences within the proximal 1 kb promoter region. This upstream regulatory domain encompasses CpG methylation sites and additional cis-acting elements. Cross-species sequence alignment reveals high conservation of STAT1 and NF-κB recognition motifs between human and rodent genomes, with corresponding binding affinity scores provided for each predicted regulatory site. (**C**) Schematic illustrating how particulate matter (containing metals and PAHs) induces ROS generation, membrane disruption, and ER stress, leading to heightened inflammatory signaling (↑ IL-1β, ↑IFN-γ). This inflammatory milieu activates JAK/STAT and NF-κB pathways, resulting in STAT1 phosphorylation and transcriptional repression of IGFBP3. The integrated pathway highlights the mechanistic link between PM exposure and impaired IGF signaling.

© The Author(s)
